# Prior Adaptive Semi-supervised Learning with Application to EHR Phenotyping

**Published:** 2022

**Authors:** Yichi Zhang, Molei Liu, Matey Neykov, Tianxi Cai

**Affiliations:** Department of Computer Science and Statistics, University of Rhode Island; Department of Biostatistics, Harvard T.H. Chan School of Public Health; Department of Statistics and Data Science, Carnegie Mellon University; Department of Biostatistics, Harvard T.H. Chan School of Public Health

**Keywords:** High dimensional sparse regression, regularization, single index model, semi-supervised learning, electronic health records

## Abstract

Electronic Health Record (EHR) data, a rich source for biomedical research, have been successfully used to gain novel insight into a wide range of diseases. Despite its potential, EHR is currently underutilized for discovery research due to its major limitation in the lack of precise phenotype information. To overcome such difficulties, recent efforts have been devoted to developing supervised algorithms to accurately predict phenotypes based on relatively small training datasets with gold standard labels extracted via chart review. However, supervised methods typically require a sizable training set to yield generalizable algorithms, especially when the number of candidate features, p, is large. In this paper, we propose a semi-supervised (SS) EHR phenotyping method that borrows information from both a small, labeled dataset (where both the label Y and the feature set X are observed) and a much larger, weakly-labeled dataset in which the feature set X is accompanied only by a surrogate label S that is available to all patients. Under a *working* prior assumption that S is related to X only through Y and allowing it to hold *approximately*, we propose a prior adaptive semi-supervised (PASS) estimator that incorporates the prior knowledge by shrinking the estimator towards a direction derived under the prior. We derive asymptotic theory for the proposed estimator and justify its efficiency and robustness to prior information of poor quality. We also demonstrate its superiority over existing estimators under various scenarios via simulation studies and on three real-world EHR phenotyping studies at a large tertiary hospital.

## 1. Introduction

Electronic Health Records (EHRs) provide a large and rich data source for biomedical research aiming to further our understanding of disease progression and treatment response. EHR data has been successfully used to gain novel insights into a wide range of diseases, with examples including diabetes (Brownstein et al., 2010), rheumatoid arthritis (Liao et al., 2014), inflammatory bowl disease (Ananthakrishnan et al., 2014), and autism (Doshi-Velez et al., 2014). EHR is also a powerful discovery tool for identifying novel associations between genomic markers and multiple phenotypes through analyses such as phenome-wide association studies (Denny et al., 2010; Kohane, 2011; Wilke et al., 2011; Cai et al., 2018).

Despite its potential, ensuring unbiased and powerful biomedical studies using EHR is challenging because EHR was primarily designed for patient care, billing, and record keeping. Extracting precise phenotype information for an individual patient requires manual medical chart reviews, an expensive process that is not scalable for research studies. To overcome such difficulties, recent efforts including those from Informatics for Integrating Biology and the Bedside (i2b2) (Liao et al., 2015; Yu et al., 2015, e.g.) and the Electronic Medical Records and Genomics (eMERGE) network (Newton et al., 2013; Gottesman et al., 2013) have been devoted to developing phenotyping algorithms to predict disease status using relatively small training datasets with gold standard labels extracted via chart review.

Various approaches to EHR phenotyping have been proposed. Supervised machine learning methods have been shown to achieve robust performance across disease phenotypes and EHR systems (Carroll et al., 2012; Liao et al., 2015). However, supervised methods typically require a sizable training set to yield generalizable algorithms especially when the candidate features, denoted by X, is of high dimensionality p. One approach to overcome the high dimensionality is to consider unsupervised methods. Unfortunately, standard unsupervised methods such as clustering are likely to fail when the dimension of X is large, but a majority of the features are unrelated to the phenotype of interest and predictive of some other underlying subgroups. Recently, unsupervised methods based on “silver standard labels” have been proposed. These methods leverage a surrogate outcome S that is highly predictive of the true phenotype status Y, such as the count of International Classification of Diseases (ICD) billing codes of the disease, to train the phenotyping algorithm against the features X. Specifically, Halpern et al. (2016) and Zhang et al. (2020) utilized anchor variables with high positive predictive value as the surrogate S to estimate Y∣X under the conditional independence assumption S⊥X∣Y. Agarwal et al. (2016) trained penalized logistic regression on S∼X for phenotyping of Y against X. Chakrabortty et al. (2017) provided theoretical justification for this strategy. They showed that a regularized estimator constructed from an unlabeled subset consisting of those with extreme values of S can be used to infer the direction of β under single index models $S∼f\left(\alpha^{⊤}X,\epsilon \right)$) and $Y∼g\left(\beta^{⊤}X\right)$. Their method relies on the similarity between the directions of α and β to make efficient estimation. However, it is not robust to poor surrogacy resulted from violation of such assumptions. Furthermore, their method cannot be directly used to predict Y using both S and X or accurately recover the scale of Pr(Y=1∣S,X).

A number of semi-supervised or weakly supervised deep learning procedures have also been proposed recently and shown to attain better performance than the supervised counterparts. For example, Ratner et al. (2017) proposed a weakly supervised approach that trains a deep model with imperfect labels generated from user–specified label functions from sources such as patterns, heuristics, and external knowledge bases. Wang and Poon (2018) developed a framework for weak supervision from multiple sources by composing probabilistic logic with deep learning. McDermott et al. (2018) designed a semi-supervised cycle Wasserstein regression generative adversarial networks (CWR-GAN) approach using adversarial signals to learn from unlabelled samples and improve prediction performance in scarcity of gold-standard labels. However, it remains unclear when and how the surrogate features, along with the unlabeled dataset can improve the prediction performance of these deep models, due to their complex architectures.

In this paper, we propose an semi-supervised (SS) method for estimating $Y|W={\left(S,X^{⊤}\right)}^{⊤}$ that borrows information from both a small labeled dataset with n realizations of ${\left(Y,W^{⊤}\right)}^{⊤}$ and a much larger unlabeled dataset with N observations on W, under a high dimensional setting with N≫p≫n. We consider a logistic phenotype model for Y∣S,X, a single index model (SIM) for S∣X, as well as a *working* prior assumption that S is independent of X given Y. We obtain the estimator through regularization with penalty functions reflecting the prior knowledge. When the prior assumption holds exactly, we show that the unlabeled dataset can naturally be used to assist in the estimation of the phenotype model. Allowing the prior assumption to hold approximately or to be highly violated, our prior adaptive semi-supervised (PASS) estimator adaptively incorporate the prior knowledge by shrinking the estimator towards a direction derived under the prior.

The proposed PASS estimator is similar to the prior LASSO (pLASSO) procedure of Jiang et al. (2016) in that both approaches aim to incorporate prior information into the $\ell_{1}$ penalized estimator in a high-dimensional setting. Nevertheless, the differences are substantial and clear. Jiang et al. (2016) assumed that the prior information was summarized into prediction values and contributed to the likelihood term. In contrast, we use prior information to guide the shrinkage and put them into the penalty term. In this sense, PASS and pLASSO complement each other to some extent. However, as shown in both theory and simulations, putting prior information into the likelihood term tends to lead to the “take it or leave it” phenomenon: the usefulness of the prior information is determined based on the overall effect of all predictors. On the other hand, by putting prior information into the penalty term, the PASS approach provides more flexible control: it is able to scrutinize the individual effect of each predictor. This gained flexibility can result in improved theoretical and numerical performances.

The rest of this paper is organized as follows. We discuss the motivation, an important special scenario and the general methodology in Section 2. We analyze the theoretical properties of the proposed approach in Section 3, and access its finite sample performance via simulation studies in Section 4. Furthermore, we illustrate the practical value of the proposed approach on three real EHR datasets in Section 5. Finally, we conclude this paper with some discussions and extensions in Section 6. All technical proofs and additional numerical results are given in the Supplementary Materials.

## 2. Methodology

### 2.1 Setup

We assume that the underlying data consists of N independent and identically distributed (i.i.d.) observations $\left\{{\left(Y_{i},S_{i},X_{i}^{⊤}\right)}^{⊤}={\left(Y_{i},W_{i}^{⊤}\right)}^{⊤},i=1,\ldots ,N\right\}$, where $Y_{i}$ is a binary indicator of the disease status of the ith patient, $S_{i}$ is a scalar surrogate variable that is reasonably predictive of $Y_{i}$ chosen via domain knowledge, and $X_{i}$ is a p-dimensional feature vector. Examples of $S_{i}$ includes the total count of ICD codes or mentions for the disease of interest in clinical notes extracted via natural language processing (NLP). Candidate features X may include the ICD9 code counts for competing diagnosis, lab results, as well as NLP mentions of relevant signs/symptoms, medications and procedures. We may also include various transformations of original features in X to account for non-linear effects. While $\left\{W_{i},i=1,\ldots ,N\right\}$ is fully observed, $Y_{i}$ is only observed for a random subset of n patients. Hence the observed data are 𝓛∪𝓤, where without loss of generality, the first n observations are assumed fully observed as $𝓛=\left\{{\left(Y_{i},W_{i}^{⊤}\right)}^{⊤},i=1,\ldots ,n\right\}$, and the rest constitute the unlabeled dataset as $𝓤=\left\{W_{i},i=n+1,\ldots ,N\right\}$.

Throughout, for a d-dimensional vector u, the $\ell_{q}$-norm of v is $∥v{∥}_{q}={\left(\sum_{j=1}^{d}{\left|v_{j}\right|}^{q}\right)}^{1/q}$. The $\ell_{\infty }$-norm of v is $∥v{∥}_{\infty }=\max_{1\leq j\leq d}\left|v_{j}\right|$. The support of v is $\text{supp}(v)=\left\{j:v_{j}\neq 0\right\}$. If 𝓘 is a subset of {1,…,p}, then $v_{𝓘}$ denotes a d-dimensional vector whose jth element is $v_{j}1_{j\in 𝓘}$, and $1_{B}$ is the indicator function for set B. The independence between random variables/vectors U and V is written as U⫫V. We also denote the negative log-likelihood function associated with the logistic model with $\ell (y,\eta )=-y\eta +\log\left(1+e^{\eta }\right)$.

### 2.2. Model Assumptions

To predict Y using $W={\left(S,X^{⊤}\right)}^{⊤}$, we assume

(*𝓜_Y_*) $$\mathrm{Pr}(Y=1|W)=\sigma \left(\zeta_{0}+S\gamma_{0}+X^{⊤}\beta_{0}\right)=\sigma \left(\vartheta_{0}^{⊤}\vec{W}\right)\;\text{with}\;\vartheta_{0}={\left(\zeta_{0},\gamma_{0},\beta_{0}^{⊤}\right)}^{⊤},$$

where for any vector w, $\vec{w}={\left(1,w^{⊤}\right)}^{⊤}$, and $\sigma (t)=e^{t}/\left(1+e^{t}\right)$. To leverage the data in 𝓤, we further assume a single index model (SIM) for S∣X, i.e. there exists $\alpha_{0}\in {\mathbb{R}}^{p}$ such that

(*𝓜_S_*) $$S=f\left(X^{⊤}\alpha_{0},\epsilon \right),\;\text{with some}\;\epsilon ⫫X\;\text{and}\;f\;\text{satisfying}\;E\left\{f^{2}\left(X^{⊤}\alpha_{0},\epsilon \right)\right\}<\infty ,$$

where $X^{⊤}\alpha_{0}$ is a single linear combination of the features X and f is an unknown link function. Here $\zeta_{0}$, $\gamma_{0}$, $\beta_{0}$ and $\alpha_{0}$ are parameters to be estimated where only the direction of $\alpha_{0}$ is identifiable and its norm does not affect our construction introduced below. If $\alpha_{0}$ and $\beta_{0}$ are similar in certain ways, one would expect that the unlabeled dataset 𝓤 may be used to improve upon the standard supervised estimator for $\beta_{0}$ using 𝓛 alone. For example, if S is a noisy representation of Y with random measurement error, then it is reasonable and common in the EHR literature (Hong et al., 2019; Zhang et al., 2020, e.g.) to assume

(*𝓒*^prior^) X⫫S∣Y.

Note that a similar conditional independence assumption to (${𝓒}^{\text{prior}}$) was imposed between the input and the pretext target given the label, in the context of self-supervised learning to demonstrate its advantage (Lee et al., 2020). Under (${𝓒}^{\text{prior}}$), we have Proposition 1 with proof given in Supplementary Materials.

### Proposition 1.

*Under* $\left({𝓜}_{Y}\right)$, $\left({𝓜}_{S}\right)$, $\left({𝓒}^{\text{prior}}\right)$, *and assuming* $E\left(XX^{⊤}\right)$ *is positive–definite, and it holds that:* (C1) *for any two vectors* $a_{1}$, $a_{2}$, $E\left(X^{⊤}a_{2}|X^{⊤}a_{1}\right)$ *is linear in* $X^{⊤}a_{1}$, *there exist scalars* $k_{1}$, $k_{2}\in \mathbb{R}$ *such that* $\alpha_{0}=k_{1}\beta_{0}$ *and* $\alpha^{*}=k_{2}\beta_{0}$ *where*

$$\left(\tau^{*},\alpha^{*}\right)=\underset{\tau ,\alpha }{\text{arg min}}\;E{\left(S-\tau -X^{⊤}\alpha \right)}^{2}.$$

### Remark 1.

Condition (C1) holds for elliptical distributions including multivariate normal. By Diaconis and Freedman (1984) and Hall and Li (1993), this assumption tends to hold for non-elliptical design when the dimensionality is high. Specifically, one can show that under mild regularity conditions, for two projection vectors $a_{1}$ and $a_{2}$ uniformly randomly drawn from $S^{p-1}=\left\{v\in {\mathbb{R}}^{p-1}:∥v{∥}_{2}=1\right\}$, the pair $\left(X^{⊤}a_{2},X^{⊤}a_{1}\right)$ weakly converges to a bivariate normal distribution with high probability, and thus $E\left(X^{⊤}a_{2}|X^{⊤}a_{1}\right)$ is at least approximately linear in $X^{⊤}a_{1}$; see Theorem 1.1 of Diaconis and Freedman (1984) and equation (1.9) of Hall and Li (1993).

Proposition 1 hinges on the main result of Li and Duan (1989) that when the features X satisfy (C1), the direction of the coefficients of a SIM could be estimated using least squares regression for the response against X. It suggests that 𝓤 can greatly improve the estimation of $\beta_{0}$ under (${𝓒}^{\text{prior}}$) because the phenotype model (${𝓜}_{Y}$) may be rewritten as $\text{logit Pr}(Y=1|W)=\zeta +S\gamma +\rho X^{⊤}\alpha$ for some ρ. Under this model, a simple SS estimator for ζ, γ and β in (${𝓜}_{Y}$) can be obtained as $\hat{\zeta }$, $\hat{\gamma }$ and $\hat{\rho }\hat{\alpha }$, where

$${\left(\hat{\zeta },\hat{\gamma },\hat{\rho }\right)}^{⊤}=\underset{\zeta ,\gamma ,\rho }{\arg\min}\sum_{i=1}^{n}\ell \left(Y_{i},\zeta +\gamma S_{i}+\rho X_{i}^{⊤}\hat{\alpha }\right),\;{\left(\hat{\tau },{\hat{\alpha }}^{⊤}\right)}^{⊤}=\underset{\tau ,\alpha }{\arg\min}\sum_{i=1}^{N}{\left(S_{i}-\tau -X\alpha \right)}^{2}.$$

By doing so, the direction of the high dimensional vector β is estimated based on the entire 𝓛∪𝓤, and only the parameters ${\left(\zeta ,\gamma ,\rho \right)}^{⊤}$ are estimated using the small labeled dataset 𝓛. Hereafter we shall refer to this SS estimator derived under (${𝓒}^{\text{prior}}$) as ${\text{SS}}^{\text{prior}}$.

Nevertheless, ${\text{SS}}^{\text{prior}}$ is only valid when (${𝓒}^{\text{prior}}$) and (C1) holds exactly. Our goal is to develop a more robust SS estimator under (${𝓜}_{Y}$) and (${𝓜}_{S}$) that can efficiently exploit 𝓤 when (${𝓒}^{\text{prior}}$) and (C1) may only hold approximately. In this more general setting, a desirable SS estimator should improve upon the standard supervised estimator when the directions of $\alpha_{0}$ and $\beta_{0}$ are similar in their magnitude and/or support. In addition, it should perform similarly to the supervised estimator when the two directions are not close. We shall now detail our PASS estimation procedure which automatically adapts to different cases as reflected in the observed data.

### 2.3 Prior Adaptive Semi-Supervised (PASS) Estimator

With 𝓛 only, a supervised estimator for β can be obtained via the standard $\ell_{1}$-penalized regression:

(1) $$\overset{˘}{\vartheta }={\left(\overset{˘}{\zeta },\overset{˘}{\gamma },{\overset{˘}{\beta }}^{⊤}\right)}^{⊤}=\underset{\vartheta }{\arg\min}\frac{1}{n}\sum_{i=1}^{n}\ell \left(Y_{i},\vartheta^{⊤}{\vec{W}}_{i}\right)+\lambda ∥\beta {∥}_{1}.$$

With properly chosen λ, the consistency and rate of convergence for $\overset{˘}{\vartheta }$ has been established (van de Geer, 2008). To improve the estimation of β through leveraging 𝓤, we note that when (${𝓒}^{\text{prior}}$) holds approximately, the magnitude of $\beta_{0}-\rho \alpha_{0}$ is small for some ρ, and the support of $\beta_{0}-\rho \alpha_{0}$ is of small size as well.

To incorporate such prior belief on the relationship between $\alpha_{0}$ and $\beta_{0}$, we construct the penalty term

$$\underset{\rho }{\min}\left\{\lambda_{1}\|{{\left(\beta -\rho \alpha_{0}\right)}_{{𝓐}_{0}}\|}_{1}+\lambda_{2}{\left\|{\left(\beta -\rho \alpha_{0}\right)}_{{𝓐}_{0}^{c}}\right\|}_{1}\right\},$$

where ${𝓐}_{0}=\text{supp}\left(\alpha_{0}\right)$, and $\lambda_{1}$, $\lambda_{2}>0$ are tuning parameters. Since ${\left(\alpha_{0}\right)}_{{𝓐}_{0}^{c}}=0$, the penalty term is equivalent to

(2) $$\lambda_{1}\left\{\underset{\rho }{\min}{\left\|{\left(\beta -\rho \alpha_{0}\right)}_{{𝓐}_{0}}\right\|}_{1}\right\}+\lambda_{2}{\left\|\beta_{{𝓐}_{0}^{c}}\right\|}_{1}.$$

The first term in the penalty measures how far β is from the closest vector along the $\alpha_{0}$ direction, and hence encourages smaller magnitude of $\beta -\rho \alpha_{0}$. The second term shrinks $\beta_{{𝓐}_{0}^{c}}$ towards 0, which reflects our prior that predictors irrelevant to S are likely to be irrelevant to Y as well. The tuning parameters $\lambda_{1},\lambda_{2}$ control the strength of the belief imposed. When they are sufficiently large, β will be forced to be a multiple of $\alpha_{0}$ and thus it ends up with the same estimator as in the case where (${𝓒}^{\text{prior}}$) holds.

Since we have N≫p samples to estimate $\alpha_{0}$, we use the adaptive LASSO (ALASSO) penalized least square estimator $\hat{\alpha }$ (Zou, 2006; Zou and Zhang, 2009), where

$$\hat{\tau },\hat{\alpha }=\underset{\tau ,\alpha }{\arg\min}\frac{1}{N}\sum_{i=1}^{N}{\left(S_{i}-\tau -X_{i}^{⊤}\alpha \right)}^{2}+\mu \sum_{j=1}^{p}{\hat{\omega }}_{j}\left|\alpha_{j}\right|,$$

where ${\hat{\omega }}_{j}={\left|{\hat{\alpha }}_{\text{init},j}\right|}^{-\nu }$ for some constant ν>0, ${\hat{\alpha }}_{\text{init}}={\left({\hat{\alpha }}_{\text{init},1},\ldots ,{\hat{\alpha }}_{\text{init},p}\right)}^{⊤}$,

$${\hat{\tau }}_{\text{init}},{\hat{\alpha }}_{\text{init}}=\underset{\tau ,\alpha }{\arg\min}\frac{1}{N}\sum_{i=1}^{N}{\left(S_{i}-\tau -X_{i}^{⊤}\alpha \right)}^{2}+\mu_{\text{init}}∥\alpha {∥}_{1},$$

$\mu_{\text{init}}$ and μ are tuning parameters that can be chosen via the cross-validation or Bayesian information criterion (BIC). Here, $\hat{\alpha }$ is actually an estimator of $\alpha^{*}$, which has the same direction as $\alpha_{0}$ under the conditions in Proposition 1.

Appending the penalty term (2) to the likelihood and replacing $\alpha_{0}$ with its estimate $\hat{\alpha }$, we propose to estimate $\vartheta_{0}={\left(\zeta_{0},\gamma_{0},\beta_{0}^{⊤}\right)}^{⊤}$ by

$$\hat{\vartheta }={\left(\hat{\zeta },\hat{\gamma },{\hat{\beta }}^{⊤}\right)}^{⊤}=\underset{\vartheta }{\arg\min}\frac{1}{n}\sum_{i=1}^{n}\ell \left(Y_{i},\vartheta {\vec{W}}_{i}\right)+\lambda_{1}\left\{\underset{\rho }{\min}{\left\|{\left(\beta -\rho \hat{\alpha }\right)}_{\hat{𝓐}}\right\|}_{1}\right\}+\lambda_{2}{\left\|\beta_{{\hat{𝓐}}^{c}}\right\|}_{1},$$

where $\hat{𝓐}=\text{supp}(\hat{\alpha })$. The estimators can be equivalently obtained as

(3) $$\hat{\rho },\hat{\vartheta }=\underset{\rho ,\vartheta }{\arg\min}\frac{1}{n}\sum_{i=1}^{n}\ell \left(Y_{i},\vartheta {\vec{W}}_{i}\right)+\lambda_{1}{\left\|{\left(\beta -\rho \hat{\alpha }\right)}_{\hat{𝓐}}\right\|}_{1}+\lambda_{2}{\left\|\beta_{{\hat{𝓐}}^{c}}\right\|}_{1}$$

The impact of the tuning parameters $\lambda_{1},\lambda_{2}$ can be understood from a bias-variance tradeoff viewpoint. When $\lambda_{j}$’s are large, $\hat{\beta }$ tends to be a multiple of $\hat{\alpha }$ and thus is an estimator with high bias and low variance. In contrast, when $\lambda_{j}$’s are small, the likelihood term based on the labeled dataset 𝓛 is the dominant part, and hence $\hat{\beta }$ will have low bias and high variance. By varying the values of $\lambda_{j}$’s, we are able to obtain a continuum connecting these two extremes. In practice, $\lambda_{1}$ and $\lambda_{2}$ can be chosen via standard data-driven approaches such as cross-validation.

### 2.4 Computation Details

The minimization in (3) can be solved with standard software for LASSO estimation. Let $\delta =\beta -\rho \hat{\alpha }$. We can re-parametrize the expression above in terms of ρ, ζ, γ, and δ as

$$\hat{\zeta },\hat{\gamma },\hat{\rho },\hat{\delta }=\underset{\zeta ,\gamma ,\rho ,\delta }{\arg\min}\frac{1}{n}\sum_{i=1}^{n}\ell \left(Y_{i},\zeta +S_{i}\gamma +\rho X_{i}^{⊤}\hat{\alpha }+X_{i}^{⊤}\delta \right)+\lambda_{1}\left({\left\|\delta_{\hat{𝓐}}\right\|}_{1}+\kappa {\left\|\delta_{𝓟\setminus \hat{𝓐}}\right\|}_{1}\right),$$

where 𝓟={1,…,p} and $\kappa =\lambda_{2}/\lambda_{1}$. This is a typical LASSO problem with covariates ${\left(1,S_{i},X_{i}^{⊤}\hat{\alpha },X_{i}^{⊤}\right)}^{⊤}$, parameters ${\left(\zeta ,\gamma ,\rho ,\delta \right)}^{⊤}$, and a weighted $\ell_{1}$ penalty on the parameters. Hence it can be solved by essentially any algorithm for ALASSO fitting. In this paper, we use the R package glmnet (Friedman et al., 2010) to compute $\hat{\zeta }$, $\hat{\gamma }$, $\hat{\rho }$, and $\hat{\delta }$, and construct the final estimator for $\vartheta_{0}$ as $\hat{\vartheta }={\left(\hat{\zeta },\hat{\gamma },{\hat{\beta }}^{⊤}\right)}^{⊤}$ with $\hat{\beta }=\hat{\delta }+\hat{\rho }\hat{\alpha }$.

## 3. Theoretical Properties

In this section, we present non-asymptotic risk bounds for the PASS estimator. We also make theoretical comparisons with the supervised LASSO estimator to shed light on when PASS outperforms the LASSO and where such improvement comes from.

### 3.1 Notations

A random variable V is sub-Gaussian $\left(\tau^{2}\right)$ if $E\{\exp(\lambda |V|)\}\leq 2\exp\left(\lambda^{2}\tau^{2}/2\right)$ holds for all λ>0. Throughout, we define

$$U={\left(X^{⊤},1\right)}^{⊤},K=E\left(UU^{⊤}\right),\xi ={\left(\alpha^{⊤},\tau \right)}^{⊤},Z_{\alpha }={\left(X^{⊤},X^{⊤}\alpha ,S,1\right)}^{⊤},G=E\left(Z_{\alpha^{*}}Z_{\alpha^{*}}^{⊤}\right),$$

$$\theta ={\left(\delta^{⊤},\rho ,\gamma ,\zeta \right)}^{⊤},H=E\left[\sigma \left(Z_{\alpha^{*}}^{⊤}\theta_{0}\right)\left\{1-\sigma \left(Z_{\alpha^{*}}^{⊤}\theta_{0}\right)\right\}Z_{\alpha^{*}}Z_{\alpha^{*}}^{⊤}\right],$$

where $\alpha^{*}$ is given by ${\left(\alpha^{*⊤},\tau^{*}\right)}^{⊤}=\xi^{*}={\text{arg min}}_{\xi }E{\left(S-U^{⊤}\xi \right)}^{2}$, and ${\text{Θ}}_{0}=\{\theta :\delta +\rho \alpha^{*}=\beta_{0},\zeta =\zeta_{0},\gamma =\gamma_{0}\}$. Denote by ${𝓑}_{0}=\text{supp}\left(\beta_{0}\right)$, ${𝓐}^{*}=\text{supp}\left(\alpha^{*}\right)$ and $q^{*}=\left|{𝓐}^{*}\right|$. We assume ${\left\|\alpha^{*}\right\|}_{2}=1$ without loss of generality since $\alpha^{*}$ is used to recover only the direction of $\beta_{0}$ in SIM and one can change ρ correspondingly to make any $\beta =\delta +\rho \alpha^{*}$ invariant to ${\left\|\alpha^{*}\right\|}_{2}$. Note that under (${𝓜}_{Y}$), any $\theta_{0}\in {\text{Θ}}_{0}$ minimizes $E\left\{\ell \left(Y,Z_{\alpha^{*}}^{⊤}\theta \right)\right\}$, and due to perfect multicollinearity in $Z_{\alpha^{*}}$, $\theta_{0}$ is not unique. However, any $\theta_{0}\in {\text{Θ}}_{0}$ corresponds to the unique $\beta_{0}=\delta_{0}+\rho_{0}\alpha^{*}$ and thus $Z_{\alpha^{*}}^{⊤}\theta_{0}=\zeta_{0}+S\gamma_{0}+X^{⊤}\beta_{0}=\vartheta_{0}^{⊤}\vec{W}$ is well-defined. Moreover, any quantity depending on $\theta_{0}$ through $Z_{\alpha^{*}}^{⊤}\theta_{0}$ is well-defined. Since the main results in this section depend on $\theta_{0}$ solely through $Z_{\alpha^{*}}^{⊤}\theta_{0}$, we will use $\theta_{0}$ to represent any $\theta \in {\text{Θ}}_{0}$ for simplicity.

For $\theta ={\left(\delta^{⊤},\rho ,\gamma ,\zeta \right)}^{⊤}$, define $\text{Ω}(\theta )=\lambda_{0}(|\rho |+|\gamma |+|\zeta |)+\lambda_{1}{\left\|\delta_{{𝓐}^{*}}\right\|}_{1}+\lambda_{2}{\left\|\delta_{𝓟\setminus {𝓐}^{*}}\right\|}_{1}$, ${\text{Δ}}_{\alpha }=2\mu_{\text{init}}q^{*}/\varphi^{2}$ and Π(θ)=|ρ|, where φ is a constant defined by Assumption (A4) in Section S2.1 of the Supplement Material, and $\lambda_{0}=36B{\left\{\log(6/\epsilon )/n\right\}}^{1/2}$. To introduce the oracle $\theta^{*}$, we define the oracle risk function as:

(4) $$𝓔\left(\theta ,{𝓢}_{+},{𝓢}_{-}\right)=E\ell \left(Y,Z_{\alpha^{*}}^{⊤}\theta \right)-E\ell \left(Y,Z_{\alpha^{*}}^{⊤}\theta_{0}\right)\;\;+256\frac{\kappa {\left({𝓢}_{+}\right)}^{2}\left|{𝓢}_{+}\right|}{\varpi \psi \left({𝓢}_{+}\right)}+8\lambda_{1}{\left\|\theta_{{𝓢}_{-}\cap {𝓐}^{*}}\right\|}_{1}+8\lambda_{2}{\left\|\theta_{{𝓢}_{-}\cap \left(𝓟\setminus {𝓐}^{*}\right)}\right\|}_{1}+8\lambda_{1}{\text{Δ}}_{\alpha }\text{Π}(\theta ),$$

where

$$\psi \left({𝓢}_{+}\right)=\underset{v:\text{Ω}\left(v_{{𝓢}_{-}}\right)\leq 3\text{Ω}\left(v_{{𝓢}_{+}}\right)}{\inf}\frac{v^{⊤}Gv}{v_{{𝓢}_{+}}^{⊤}v_{{𝓢}_{+}}},$$

$$\kappa \left({𝓢}_{+}\right)=\{\begin{array}{ll} \lambda_{0}, & \text{if}\;{𝓢}_{+}\cap {𝓐}^{*}=\text{Ø}\;\text{and}\;{𝓢}_{+}\cap \left(𝓟\setminus {𝓐}^{*}\right)=\text{Ø}\\ \lambda_{2}, & \text{if}\;{𝓢}_{+}\cap {𝓐}^{*}=\text{Ø}\;\text{and}\;{𝓢}_{+}\cap \left(𝓟\setminus {𝓐}^{*}\right)\neq \text{Ø}\\ +\infty , & \;\text{if}\;{𝓢}_{+}\cap {𝓐}^{*}\neq \text{Ø} \end{array}$$

Define $\theta^{*}={\left(\delta^{*⊤},\rho^{*},\gamma^{*},\zeta^{*}\right)}^{⊤}$, ${𝓢}_{+}^{*}$ and ${𝓢}_{-}^{*}$ as the solution to

$$\underset{\left\{\theta ,{𝓢}_{+},{𝓢}_{-}\right\}:{𝓢}_{+}\cap {𝓢}_{-}=\emptyset ,{𝓢}_{+}\cup {𝓢}_{-}=\text{supp}(\theta )\cup \bar{𝓟},{𝓢}_{+}⊇\bar{𝓟},\;\text{and}\;{\left\|G^{1/2}\left(\theta -\theta_{0}\right)\right\|}_{2}\leq \eta ,}{\arg\min}𝓔\left(\theta ,{𝓢}_{+},{𝓢}_{-}\right)$$

where $\bar{𝓟}=\{p+1,p+2,p+3\}$, and η is a constant as defined by Assumption (A3) in Section S2.1 of the Supplement. Let ${𝓢}^{*}={𝓢}_{+}^{*}\cup {𝓢}_{-}^{*}=\text{supp}\left(\theta^{*}\right)\cup \bar{𝓟}$, $\kappa^{*}=\kappa \left({𝓢}_{+}^{*}\right)$, and $\beta^{*}=\delta^{*}+\rho^{*}\alpha^{*}$. Intuitively, one may view ${𝓢}_{+}$ as the union set of unpenalized predictors and the predictors with large coefficients but not recovered by ${𝓐}^{*}$. While ${𝓢}_{-}$ can be viewed as the union set of predictors with small nonzero coefficients and the predictors recovered by ${𝓐}^{*}$. Partitioning the support of θ into ${𝓢}_{+}$ and ${𝓢}_{-}$ is inspired by Bühlmann and Van De Geer (2011, Section 6.2.4), which leads to a refined bound.

### 3.2 Main result

We first establish the risk bounds for the PASS estimator in the following theorem. Its proof can be found in Section S2 of the Supplementary Materials.

### Theorem 1.

*For any* ϵ>0, *if the assumptions (A1)–(A8) (introduced in Section S2.1 of the Supplementary Materials) hold, the following inequalities hold simultaneously with probability at least* 1−10ϵ:

$$\begin{array}{ll} Excess\;risk: & E\ell \left(Y,Z_{\hat{\alpha }}^{⊤}\hat{\theta }\right)-E\ell \left(Y,Z_{\alpha^{*}}^{⊤}\theta_{0}\right)\leq \text{Ξ,} \end{array}$$

$$\begin{array}{ll} Linear\;prediction\;error: & E{\left(Z_{\hat{\alpha }}^{⊤}\hat{\theta }-Z_{\alpha^{*}}^{⊤}\theta_{0}\right)}^{2}\leq \text{Ξ}/\varpi , \end{array}$$

$$\begin{array}{ll} Probability\;prediction\;error: & E{\left\{\sigma \left(Z_{\hat{\alpha }}^{⊤}\hat{\theta }\right)-\sigma \left(Z_{\alpha^{*}}^{⊤}\theta_{0}\right)\right\}}^{2}\leq \text{Ξ}/\varpi , \end{array}$$

*where* ϖ *is a positive constant defined in (A2)*, $\text{Ξ}=64𝓔\left(\theta^{*},{𝓢}_{+}^{*},{𝓢}_{-}^{*}\right)$, *and* 𝓔 *is an oracle risk function as defined in* equation (4).

### Remark 2.

*As detailed in Section S2.1 of the Supplement Material, Assumptions (A1)–(A8) are imposed on tail behaviour of the regression residuals, regularity of the design matrix, minimum signal strength of* $\alpha^{*}$, *sample sizes and rates of the tuning parameters. These assumptions are commonly used conditions in the theoretical literature of LASSO, such as the sub-Gaussian variable condition and the restricted eigenvalue condition; see e.g. van de Geer and Bühlmann (2009); Bickel et al. (2009); Bühlmann and Van De Geer (2011)*.

### Remark 3.

*The last term of the risk bound* $𝓔\left(\theta^{*},{𝓢}_{+}^{*},{𝓢}_{-}^{*}\right)$ *is of order* $O\left(\lambda_{1}{\text{Δ}}_{\alpha }\left|\rho^{*}\right|\right)$, *which reflects the estimation error in* $\hat{\alpha }$. *Following Lemma S8 in the Supplement, one can show that* ${\text{Δ}}_{\alpha }=O_{p}\left(N^{-1/2}\left|{𝓐}^{*}\right|\right)$. *All the other terms in* Ξ *describe the estimation error in* $\hat{\theta }$ *as if* $\hat{\alpha }$ *is replaced with* $\alpha^{*}$. *When* N *is sufficiently large*, $O\left(\lambda_{1}{\text{Δ}}_{\alpha }\left|\rho^{*}\right|\right)$ *is typically negligible relative to other terms. Specifically, if* $N\gg n{\left|{𝓐}^{*}\right|}^{2}\log(p)$, $O\left(\lambda_{1}{\text{Δ}}_{\alpha }\left|\rho^{*}\right|\right)=O\left({\left\{Nn\right\}}^{-1/2}\log{\left(p\right)}^{1/2}\left|{𝓐}^{*}\right|\right)=o\left(n^{-1}\right)$. *In general, as long as* N≫max(n,p) *and* $\alpha^{*}$ *is not much denser than* $\beta_{0}$ *as in the typical EHR application cases*, $O\left(\lambda_{1}{\text{Δ}}_{\alpha }\left|\rho^{*}\right|\right)$ *is dominated by the risk of the supervised LASSO estimator and even the supervised oracle estimator obtained under the knowledge of* supp($\beta_{0}$).

To gain a better understanding of how the key quantity Ξ in Theorem 1 changes with respect to the similarity between the prior information $\alpha^{*}$ and the target $\beta_{0}$, we shall discuss several specific cases in Section 3.3, based on the risk bound derived in Theorem 1.

### 3.3 Specific Cases

**Figure 1:**
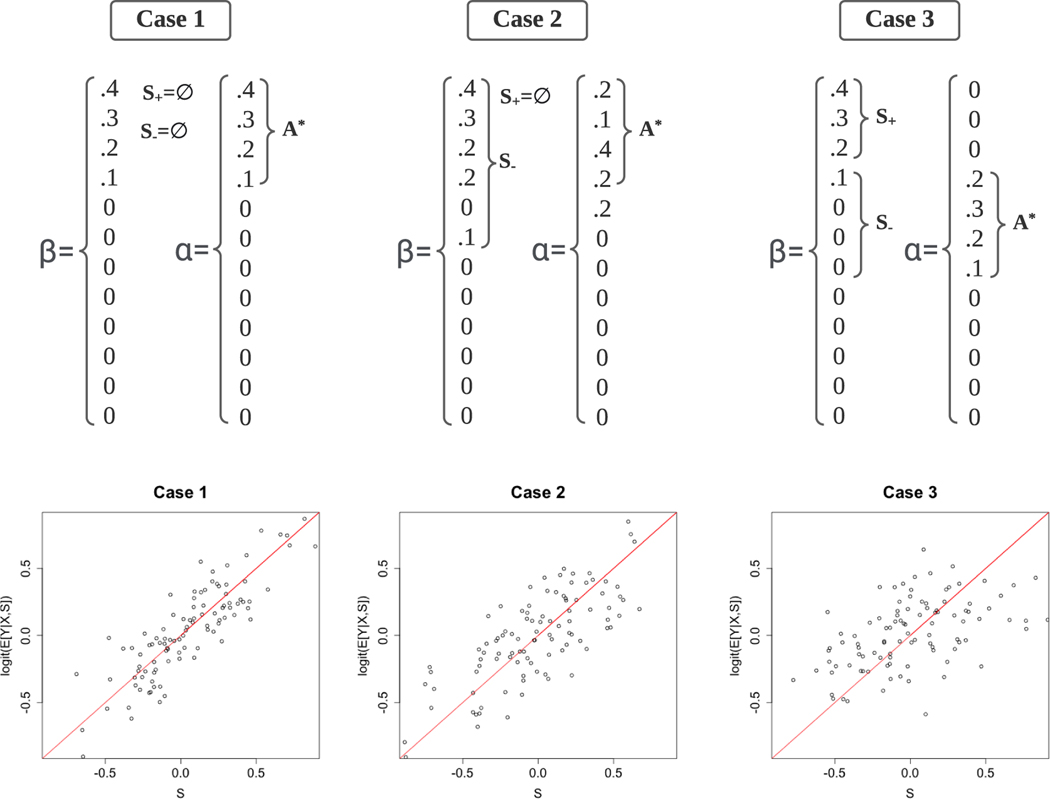
Examples of the coefficients $\beta_{0}$ and $\alpha^{*}$ in the three cases of Section 3.3. Labels ${\text{S}}_{+}$, ${\text{S}}_{-}$, and ${\text{A}}^{*}$ in the diagrams represent ${\bar{𝓢}}_{+}\setminus \bar{𝓟}$, ${𝓢}_{-}$, and ${𝓐}^{*}$ as chosen and defined in Cases 1–3. $\beta_{0}$ and $\alpha^{*}$ are aligned for comparison of their directions and supports. Presented below are scatter plots for $\sigma^{-1}\{\mathrm{Pr}(Y=1|S,X)\}$ against S of the simulated samples generated under Cases 1–3. Case 1 (presented in the left panel): $\alpha^{*}$ recovers both the support and direction of $\beta_{0}.\;\sigma^{-1}\{\mathrm{Pr}(Y=1|S,X)\}$ shows strong collinearity with S. PASS largely outperforms supervised LASSO and has the same convergence rate as the low dimensional regression. Case 2 (middle): $\alpha^{*}$ (nearly) recovers the support but not the direction $\beta_{0}.\;\sigma^{-1}\{\mathrm{Pr}(Y=1|S,X)\}$ shows moderate collinearity with S. PASS still outperforms both supervised LASSO and pLASSO in terms of convergence rate. Case 3 (right): $\alpha^{*}$ fails to recover the support of $\beta_{0}.\;\sigma^{-1}\{\mathrm{Pr}(Y=1|S,X)\}$ shows weak collinearity with S. PASS is of the same convergence rate as supervised LASSO.

Following Remark 3, we focus our discussions on the settings where N is sufficiently large such that the last term of the risk bound is negligible. We consider three different scenarios as illustrated in Figure 1: (Case 1) $\alpha^{*}$ recovers both the support and direction of $\beta_{0}$; (Case 2) $\alpha^{*}$ almost recovers the support of $\beta_{0}$ but has a substantially different direction from $\beta_{0}$; (Case 3) $\alpha^{*}$ fails to recover the support of $\beta_{0}$ (let alone its direction) and provides poor information. These three cases depict perfect, good, and poor qualities of the prior information $\alpha^{*}$ in recovering the support and direction of $\beta_{0}$. Next, we rigorously characterize the three cases by properly specifying the parameters ρ, δ, ${𝓢}_{+}$, and ${𝓢}_{-}$, and derive the convergence rate of Ξ, the risk bound of the PASS estimator, based on Theorem 1.

### Case 1.

*Let* $\bar{\rho }=\min_{\rho }{\left\|\beta_{0}-\rho \alpha^{*}\right\|}_{1}$, $\bar{\delta }=\beta_{0}-\bar{\rho }\alpha^{*}$, $\bar{\theta }={\left({\bar{\delta }}^{⊤},\bar{\rho },\gamma_{0},\zeta_{0}\right)}^{⊤}$, ${\bar{𝓢}}_{+}=\bar{𝓟}$ *and* ${\bar{𝓢}}_{-}=\text{supp}\left(\delta_{0}\right)$. *If* $\alpha^{*}$ *successfully recovers the support and direction of* $\beta_{0}$ *(see the left panel in Figure 1)*, ${\bar{𝓢}}_{-}\approx \text{Ø}$ *and* ${\left\|\delta_{0}\right\|}_{1}\approx 0$. *Since* ${\left\|G^{1/2}\left(\bar{\theta }-\theta_{0}\right)\right\|}_{2}=0$ *and* ${\bar{𝓢}}_{+}\cap {𝓐}^{*}=\text{Ø}$, *we have* $\text{Ξ}=O\left\{𝓔\left(\bar{\theta },{\bar{𝓢}}_{+},{\bar{𝓢}}_{-}\right)\right\}$ *by the definition of* $\theta^{*}$. *Hence by Theorem 1, the excess risk of* $\hat{\theta }$

$$\text{Ξ}=O_{p}\left(\lambda_{0}^{2}+\lambda_{1}{\left\|{\bar{\delta }}_{{\bar{𝓢}}_{-}\cap {𝓐}^{*}}\right\|}_{1}+\lambda_{2}{\left\|{\bar{\delta }}_{{\bar{𝓢}}_{-}\cap {𝓐}^{*c}}\right\|}_{1}\right)\approx O_{p}\left(\lambda_{0}^{2}\right)=O_{p}\left(n^{-1}\right),$$

*recalling that* $\lambda_{0}=O\left(n^{-1/2}\right)$.

As a standard result (Negahban et al., 2009), the rate of the excess risk of the supervised LASSO estimator is either $O_{p}\left\{n^{-1}\log(p)\left|{𝓑}_{0}\right|\right\}$ or $O\left\{n^{-1/2}\log{\left(p\right)}^{1/2}{\left\|\beta_{0}\right\|}_{1}\right\}$. These two rate bounds are established under different sparsity norms of $\beta_{0}$, and generally comparable, e.g. when order of average magnitude of the non-zero entries in $\beta_{0}$ is $n^{-1/2}\log{\left(p\right)}^{1/2}$. In comparison with them, $O_{p}\left(n^{-1}\right)$, the risk rate of PASS in Case 1, is much more refined. Further, $O_{p}\left(n^{-1}\right)$ dis actually the rate of the estimator of a low (fixed) dimensional logistic regression. Thus, if $\beta_{0}$ is very close to a multiple of $\alpha^{*}$, PASS could outperform the vanilla LASSO and be comparable with a low dimensional regression in terms of the convergence rate. This big gain is owing to the use of N unlabeled dataset to obtain the direction of $\beta_{0}$, and thus reduce the high dimensional regression to a low dimensional one where only the intercept and the scalar of $\beta_{0}$ need to be estimated.

### Case 2.

*Consider the same choice of* $\bar{\theta }$, ${\bar{𝓢}}_{+}$ *and* ${\bar{𝓢}}_{-}$ *as in Case 1. If* $\alpha^{*}$ *recovers the support but not the direction of* $\beta_{0}$ *(see the middle of Figure 1), we will only have* ${\left\|{\bar{\delta }}_{{\bar{𝓢}}_{-}\cap {𝓐}^{*c}}\right\|}_{1}\approx 0$ *but not* ${\left\|\delta_{0}\right\|}_{1}\approx 0$. *Then by Theorem 1, the excess risk of PASS is*

$$\text{Ξ}=O_{p}\left(\lambda_{0}^{2}+\lambda_{1}{\left\|{\bar{\delta }}_{{\bar{𝓢}}_{-}\cap {𝓐}^{*}}\right\|}_{1}+\lambda_{2}{\left\|{\bar{\delta }}_{{\bar{𝓢}}_{-}\cap {𝓐}^{*c}}\right\|}_{1}\right)\approx O_{p}\left\{n^{-1/2}\log{\left(q^{*}\right)}^{1/2}{\left\|{\bar{\delta }}_{\bar{𝓢}-\cap {𝓐}^{*}}\right\|}_{1}\right\},$$

*recalling that* $\lambda_{1}=O\left\{n^{-1/2}\log{\left(q^{*}\right)}^{1/2}\right\}$.

In Case 2, the convergence rate of the excess risk of PASS is still better than that of the supervised LASSO estimator when $q^{*}\ll p$:

$$O\left\{n^{-1/2}\log{\left(q^{*}\right)}^{1/2}{\left\|{\bar{\delta }}_{{\bar{𝓢}}_{-}\cap {𝓐}^{*}}\right\|}_{1}\right\}\ll O\left\{n^{-1/2}\log{\left(p\right)}^{1/2}{\left\|\beta_{0}\right\|}_{1}\right\},$$

by ${\left\|{\bar{\delta }}_{{\bar{𝓢}}_{-}\cap {𝓐}^{*}}\right\|}_{1}\leq \min_{\rho }{\left\|\beta_{0}-\rho \alpha^{*}\right\|}_{1}\leq {\left\|\beta_{0}\right\|}_{1}$. Namely, if $\alpha^{*}$ might not recover the direction of $\beta_{0}$ very well but the prior information ${𝓐}^{*}=\text{supp}\left(\alpha^{*}\right)$ is sparse and covers supp ($\beta_{0}$) successfully, which is reflected as ${\bar{𝓢}}_{+}=\bar{𝓟}$, the PASS estimator still benefits from the prior information. This is because recovering the support of $\beta_{0}$ reduces the dimensionality of the empirical errors needed to be controlled from p to $q^{*}=\left|{𝓐}^{*}\right|$. In this case, it is also interesting to compare the proposed PASS estimator with the prior LASSO (pLASSO) procedure of Jiang et al. (2016). When supp ($\alpha^{*}$) and supp ($\beta_{0}$) are close but the directions of $\alpha^{*}$ and $\beta_{0}$ are quite different, the pLASSO procedure is unable to utilize this information and will only result in the same convergence rate as supervised LASSO, as shown to be essentially slower than that of PASS.

### Case 3.

*Let* $\bar{\rho }=0$, $\bar{\delta }=\beta_{0}$, $\bar{\theta }={\left({\bar{\delta }}^{⊤},\bar{\rho },\gamma_{0},\zeta_{0}\right)}^{⊤}$, ${\bar{𝓢}}_{+}=\bar{𝓟}\cup \left({𝓑}_{0}\setminus {𝓐}^{*}\right)$ *and* ${\bar{𝓢}}_{-}={𝓑}_{0}\setminus {\bar{𝓢}}_{+}$. *If* $\alpha^{*}$ *fails to recover the support of* $\beta_{0}$, *i.e.* ${𝓐}^{*}\cap {𝓑}_{0}\approx \text{Ø}$ *and* ${\left\|\beta_{0,{𝓐}^{*}\cap {𝓑}_{0}}\right\|}_{1}\approx 0$, *we have* ${\left\|{\bar{\delta }}_{{\bar{𝓢}}_{-}}\right\|}_{1}\leq {\left\|{\bar{\delta }}_{{𝓐}^{*}}\right\|}_{1}\approx {\left\|\beta_{0,{𝓐}^{*}\cap {𝓑}_{0}}\right\|}_{1}\approx 0$. *Then again using Theorem 1*,

$$\text{Ξ}=O\left(\lambda_{2}^{2}\left|{\bar{𝓢}}_{+}\right|+\lambda_{1}{\left\|{\bar{\delta }}_{{\bar{𝓢}}_{-}\cap {𝓐}^{*}}\right\|}_{1}+\lambda_{2}{\left\|{\bar{\delta }}_{{\bar{𝓢}}_{-}\cap {𝓐}^{*c}}\right\|}_{1}\right)\approx O_{p}\left\{n^{-1}\log(p)\left|{𝓑}_{0}\right|\right\},$$

*recalling that* $\lambda_{2}=O\left\{n^{-1/2}\log{\left(p\right)}^{1/2}\right\}$.

In Case 3, the excess risk of the PASS is of the same order as that of supervised LASSO. Therefore the PASS approach is robust against low-quality prior information that recovers neither the direction nor the support of $\beta_{0}$. This benefit is a result of using a data-adaptive parameter ρ to control the influence of the prior information on the estimator.

## 4. Simulation Studies

### 4.1 Main setups

We conducted extensive simulation studies to examine the finite-sample performance of the PASS estimator and to compare it with existing approaches. We first considered the case where the logistic model for Y∣S,X is correctly specified, S∣X follows an SIM, and X is near elliptical, but the similarity between $\alpha_{0}$ and $\beta_{0}$ varies. Since EHR features are often zero inflated and skewed count variables, we generated $X_{500\times 1}$ from

$$X_{i}=h\left(Z_{i}\right),\;Z_{i}∼N\left(0,{\text{Σ}}_{Z}\right),\;h(t)=\log\left(1+\left[e^{t}\right]\right),$$

where [u] denotes the integer nearest to u, ${\text{Σ}}_{Z}={\left(\sigma_{i,j}\right)}_{i,j=1}^{p}$ and $\sigma_{i,j}=4{\left(0.5\right)}^{\left|i-j\right|}$. Here $\left[e^{Z_{ij}}\right]$ mimics the skewed raw EHR feature, which is typically transformed via t→log(1+t) prior to model fitting. We then generated the surrogate S from a SIM of X:

$$S_{i}=h\left(1+X_{i}^{⊤}\alpha_{0}+\epsilon_{i}\right),\;\text{with}\;\epsilon_{i}∼N\left(0,2^{2}\right).$$

Following the model assumption (${𝓜}_{Y}$), the disease status $Y_{i}$ was generated from

$$\sigma^{-1}\left\{\text{Pr}\left(Y_{i}=1|W_{i}\right)\right\}=-4+0.5S_{i}+X_{i}^{⊤}\beta_{0}.$$

To mimic different qualities of the prior information one could encounter in practice, we design six scenarios with different similarities between the true $\beta_{0}$ and $\alpha_{0}$:

$$\begin{array}{lll} \text{I:} & \alpha_{0}={\left(a_{1}^{⊤},a_{2}^{⊤},0_{p-10}^{⊤}\right)}^{⊤}, & \beta_{0}=1.5{\left(a_{1}^{⊤},a_{2}^{⊤},0_{p-10}^{⊤}\right)}^{⊤}; \end{array}$$

$$\begin{array}{lll} \text{II:} & \alpha_{0}={\left(a_{1}^{⊤},a_{2}^{⊤},0_{p-10}^{⊤}\right)}^{⊤}, & \beta_{0}=1.5{\left(a_{1}^{⊤}+d_{1}^{⊤},a_{2}^{⊤}+d_{2}^{⊤},0_{p-10}^{⊤}\right)}^{⊤}; \end{array}$$

$$\begin{array}{lll} \text{III:} & \alpha_{0}={\left(a_{1}^{⊤},a_{2}^{⊤},a_{2}^{⊤},a_{2}^{⊤},0_{p-20}^{⊤}\right)}^{⊤}, & \beta_{0}=1.5{\left(a_{1}^{⊤}+d_{1}^{⊤},a_{2}^{⊤}+d_{2}^{⊤},0_{p-10}^{⊤}\right)}^{⊤}; \end{array}$$

$$\begin{array}{lll} \text{IV:} & \alpha_{0}={\left(a_{1}^{⊤},0_{p-5}^{⊤}\right)}^{⊤}, & \beta_{0}=1.5{\left(a_{1}^{⊤}+d_{1}^{⊤},a_{2}^{⊤}+d_{2}^{⊤},0_{p-10}^{⊤}\right)}^{⊤}; \end{array}$$

$$\begin{array}{lll} \text{V}: & \alpha_{0}={\left(a_{1}^{⊤},a_{2}^{⊤},0_{p-10}^{⊤}\right)}^{⊤}, & \beta_{0}=1.5{\left(a_{2}^{⊤},a_{1}^{⊤},0_{p-10}^{⊤}\right)}^{⊤}; \end{array}$$

$$\begin{array}{lll} \text{VI}: & \alpha_{0}={\left(a_{1}^{⊤},a_{2}^{⊤},0_{p-10}^{⊤}\right)}^{⊤}, & \beta_{0}=1.5{\left(a_{2}^{⊤},0_{5},a_{1}^{⊤},0_{p-15}^{⊤}\right)}^{⊤}. \end{array}$$

where

$$\begin{array}{ll} a_{1}={\left(0.5,1,-0.8,0.6,0.2\right)}^{⊤}, & d_{1}={\left(-0.05,-0.5,1.4,0.5,-0.6\right)}^{⊤}, \end{array}$$

$$\begin{array}{ll} a_{2}={\left(0.1,-0.2,-0.2,0.2,0.7\right)}^{⊤}, & d_{2}={\left(0.02,0.05,0.02,-0.02,-0.05\right)}^{⊤}, \end{array}$$

**Figure 2:**
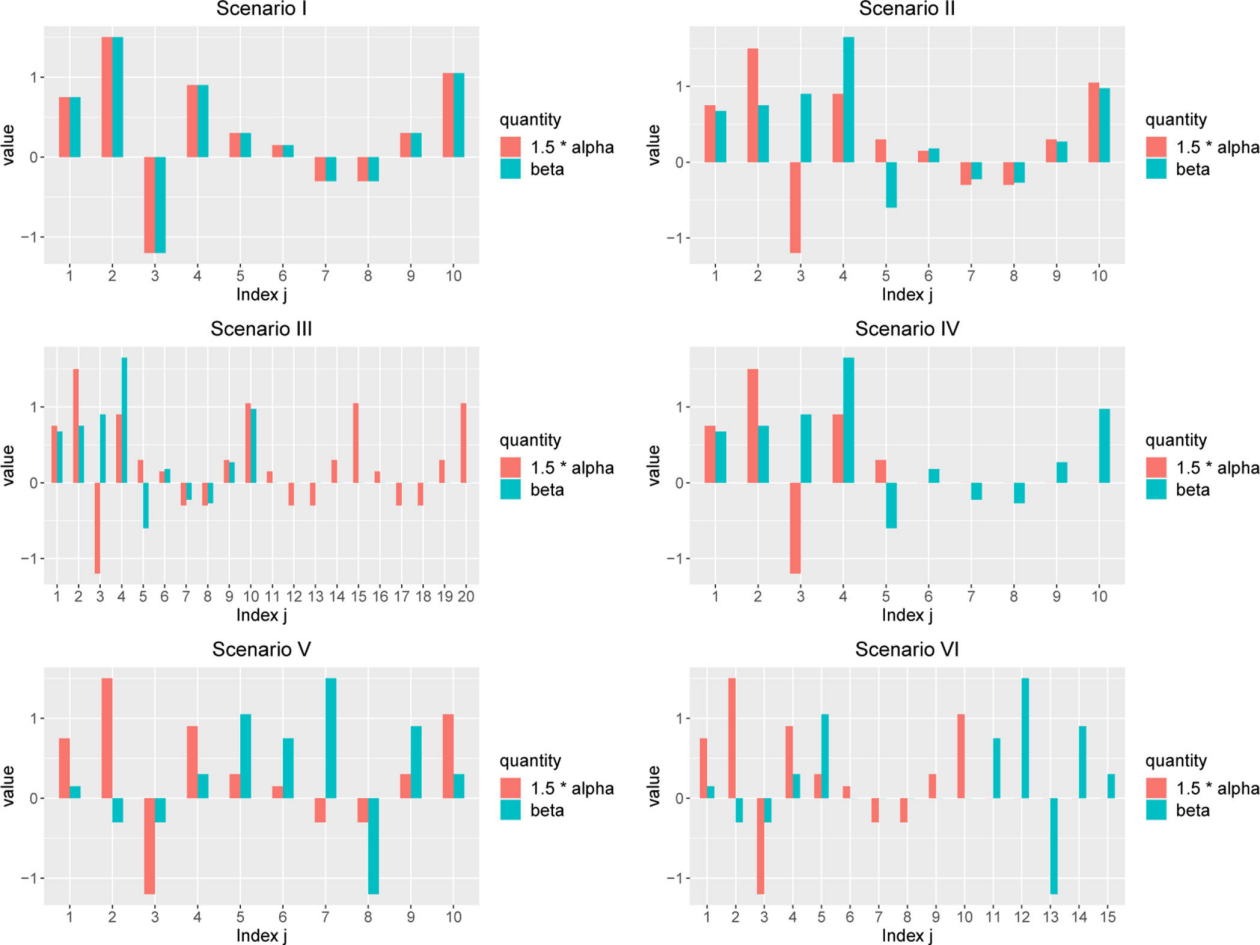
Supports and values of the coefficients $\beta_{0}$ and $1.5\alpha_{0}$ under Scenarios I–VI introduced in Section 4.1. Only those indices j satisfying $\beta_{0,j}\neq 0$ or $\alpha_{0,j}\neq 0$ are shown in the plots.

Our specifications of $\beta_{0}$ and $\alpha_{0}$ are motivated by the three key specific cases introduced in Section 3.3 and illustrated in Figure 1. Scenario I is the ideal case where $\beta_{0}$ and $\alpha_{0}$ have identical direction. In Scenario II, most of the components of $\beta_{0}$ differ slightly from a scalar multiple of $\alpha_{0}$, while a few components differ substantially. Scenarios I and II are designed to examine the performance of PASS estimator when the prior information is highly or somewhat reliable. In Scenario III, $\alpha_{0}$ is denser than $\beta_{0}$ and contains quite a few weak signals. On the contrary, in Scenario IV $\beta_{0}$ is denser than $\alpha_{0}$. In Scenario V, the magnitude of $\alpha_{0}$ and $\beta_{0}$ are quite different, whereas they still share the same support. Scenarios III, IV and V are designed to examine the performance of PASS estimator with respect to different degrees of accuracy of the support information. In Scenario VI, both the magnitude and the support of $\alpha_{0}$ and $\beta_{0}$ differs substantially, which means the unlabeled dataset provides little information. This scenario allows us to see whether the PASS estimator is robust against unreliable prior information. See Figure 2 for a visualization of $\beta_{0}$ and $\rho \alpha_{0}$ across different scenarios.

We compare PASS to following existing methods: (1) supervised LASSO penalized logistic regression with n training samples (${\text{LASSO}}_{n}$); (2) supervised ALASSO penalized logistic regression with *n* training samples, denoted by ${\text{ALASSO}}_{n}$; (3) the ${\text{SS}}^{\text{prior}}$ estimator as described in section 2.2; and (4) two variants of pLASSO estimators as proposed in Jiang et al. (2016). For pLASSO, we fit a penalized logistic model with an LASSO penalty imposed on predictors outside supp $\left(\hat{\alpha }\right)$, as in equation (8) of Jiang et al. (2016), and then use the predicted probability from that model as $Y_{i}^{\text{p}}$ in equation (7) of Jiang et al. (2016), denoted by pLASSO^1^; (2) use the predicted probability given by the ${\text{SS}}^{\text{prior}}$ approach as $Y_{i}^{\text{p}}$ in equation (7) of their paper, denoted by pLASSO^2^.

Throughout, we let N=10000 and let ν=1 in the ALASSO weights. We use Bayesian information criterion (BIC) to select $\mu_{\text{init}}$ and μ in the estimation of α due to large N, and use 10-fold cross-validation to select $\lambda_{1}$, $\lambda_{2}$ for the estimation of β, so that the phenotype model is tuned towards prediction performance. We quantify the average prediction performance of the estimated linear score, ${\tilde{\vartheta }}^{⊤}\vec{W}$, with $\tilde{\vartheta }$ obtained via different methods in an independent test dataset with size 10000. For each choice of ${\tilde{\vartheta }}^{⊤}\vec{\text{W}}$, we consider the area under the receiver operating characteristic curve (AUC) for classifying Y, the excess risk (ER) as defined in Section 3, and the mean squared error of the predicted probabilities (MSE-P) which is the mean squared differences between the predicted probability and the true probability. We summarize results based on 1000 simulated datasets for each configuration.

**Figure 3:**
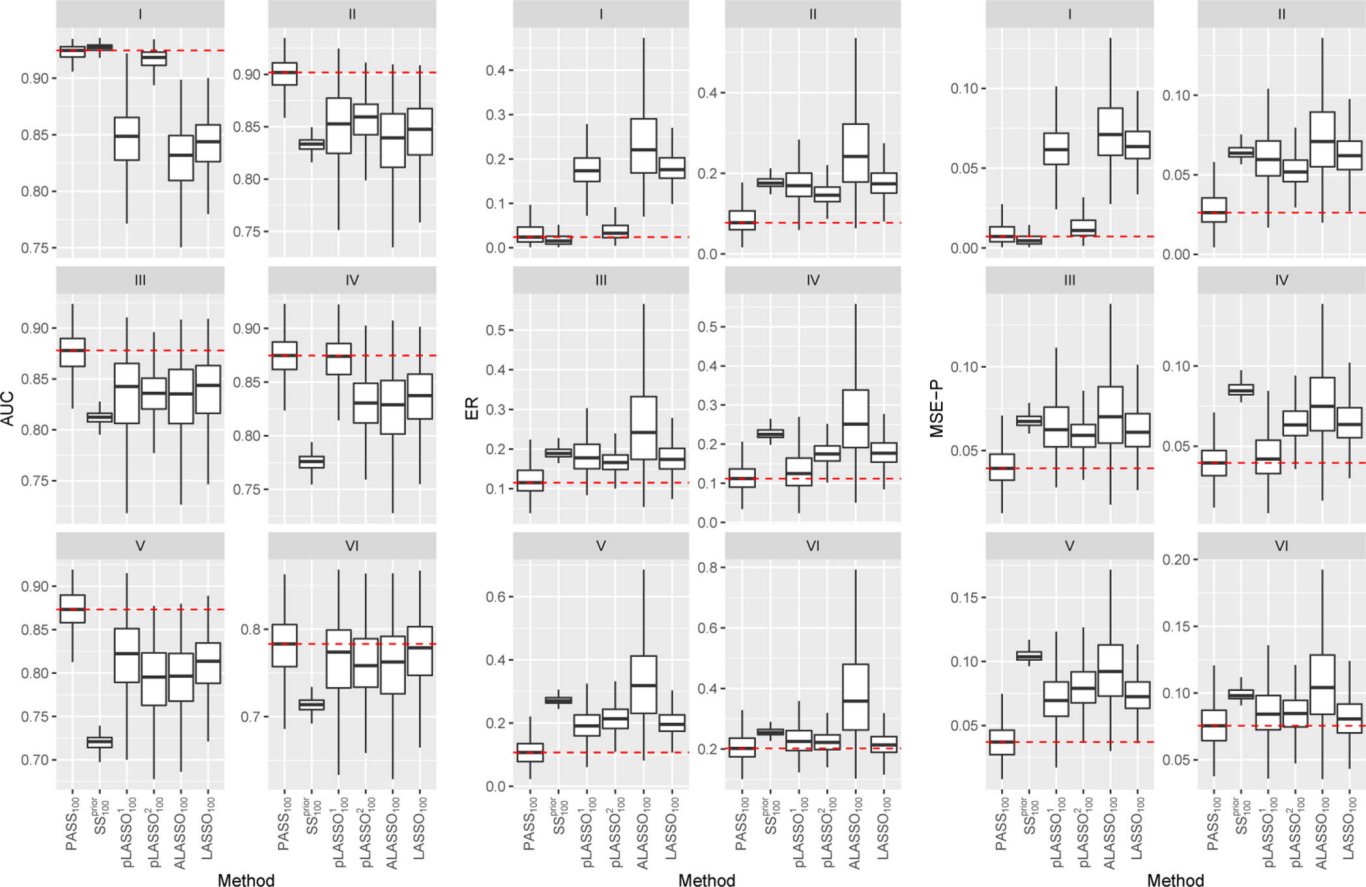
AUC (left), ER (middle) and MSE-P (right) evaluated on the test set for simulation studies under Scenarios I–VI. Outliers are not drawn. Mean performance of the PASS approach are marked using dashed lines for ease of comparison. The size of the labeled dataset is fixed at n=100.

In Figures 3, we compare prediction measures for estimators obtained with n=100. In Scenario I where the directions of $\beta_{0}$ and $\alpha_{0}$ coincide, the ${\text{SS}}^{\text{prior}}$ approach performs the best as expected, yet the proposed PASS method attained very similar accuracy followed by pLASSO^2^ which performed only slightly worse. When the directions of $\beta_{0}$ and $\alpha_{0}$ are somewhat different as in Scenario II, the ${\text{SS}}^{\text{prior}}$ and the pLASSO estimators deteriorated quickly. In contrast, the PASS estimator maintains high accuracy and outperforms all competing estimators substantially. We observe qualitatively similar patterns for Scenarios III and IV under which $\alpha_{0}$ and $\beta_{0}$ have somewhat different support. No matter whether $\alpha_{0}$ is denser than $\beta_{0}$ as in Scenario IV, or $\beta_{0}$ is denser than $\alpha_{0}$ as in Scenario V, the PASS method consistently outperforms the supervised estimators. Additionally, the performances of the ${\text{SS}}^{\text{prior}}$ and pLASSO approaches are not quite satisfactory. In Scenario V, $\beta_{0}$ and $\alpha_{0}$ have the same support but are quite different in terms of magnitude. The proposed method managed to utilize the same-support information, whereas the pLASSO approaches failed to do so. Finally, the goal of Scenario VI is to examine the robustness of the methods when $\beta_{0}$ and $\alpha_{0}$ differs a lot, possibly due to the use of an inappropriate surrogate. The PASS estimator performs similarly to the supervised estimators, indicating that our procedure is indeed adaptive to how well the data supports the prior assumption. Across all scenarios, the ALASSO approach performs slightly worse than LASSO, possibly due to the presence of some small nonzero coefficients in $\beta_{0}$.

**Figure 4:**
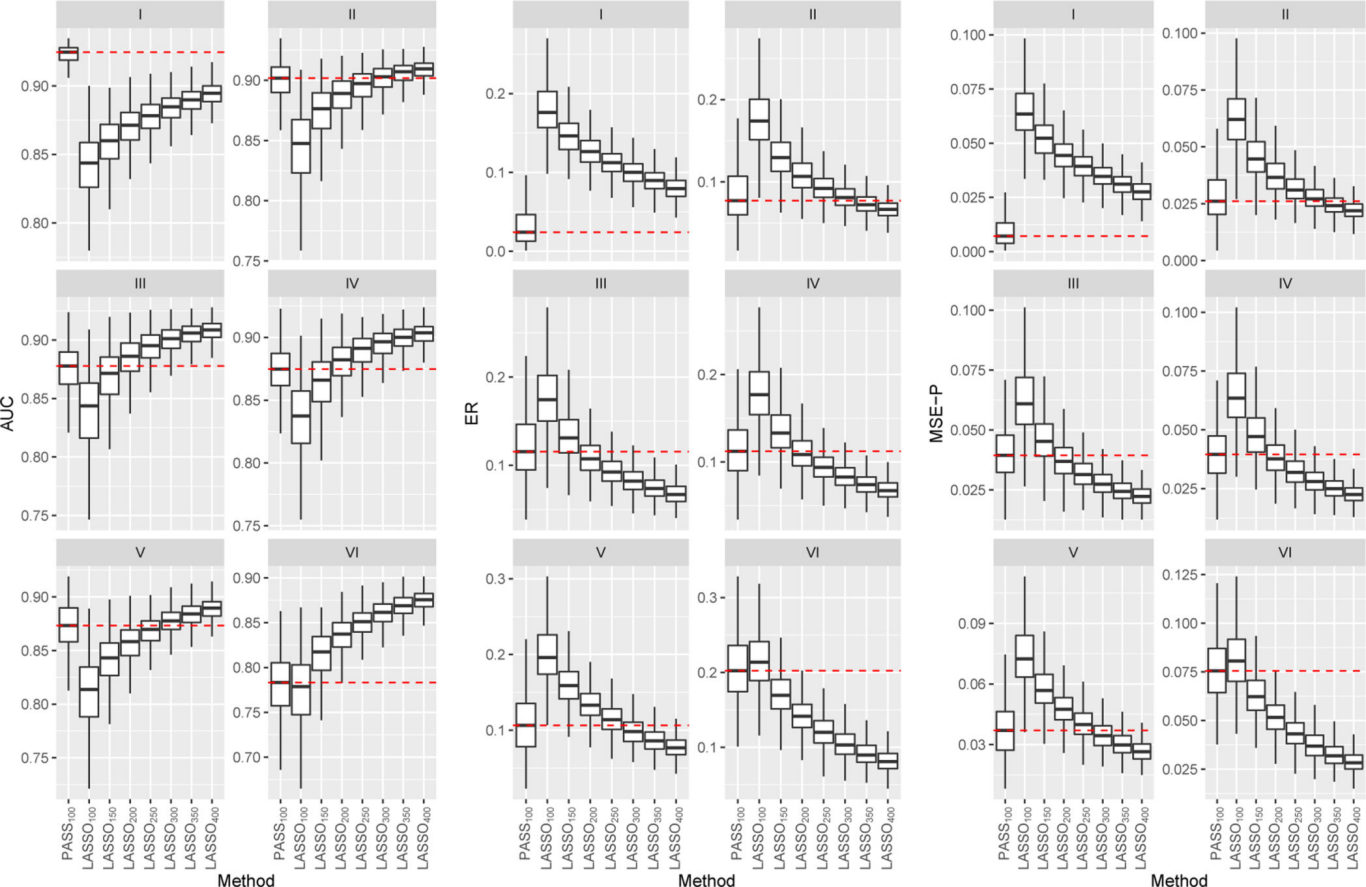
AUC (left), ER (middle) and MSE-P (right) evaluated on the test set for simulation studies under Scenarios I–VI. Outliers are not drawn. Mean performance of the PASS approach are marked using red dash lines for ease of comparison. The size of the labeled dataset is n=100 for PASS, while it varies for LASSO, as indicated in the subscripts.

In Figure 4, we present the AUC, ER and MSE-P of the PASS estimator trained with n=100 and the supervised LASSO estimator with varying label size. In Scenario I where the prior assumption holds exactly, PASS_100_, the PASS approach with 100 labeled samples, even outperforms LASSO_400_, the LASSO approach with 400 labeled samples. When the prior assumption holds approximately as in Scenarios II through V, PASS_100_ consistently outperforms LASSO_150_, and achieves similar performance as LASSO_200_, which requires twice as many labels. Finally, in Scenario VI where the prior information is highly inaccurate, the PASS method maintains comparable performance against LASSO_100_.

### 4.2 Efficiency and Robustness Evaluations under Mis-specifications

We conducted simulation studies under three additional scenarios to further investigate the efficiency and robustness of PASS when the model assumptions and elliptical design assumptions are violated. We again set p=500 and generate $X_{i}=2\text{Φ}\left(Z_{i}\right)-1$, where Φ(⋅) is the cumulative distribution function of the standard normal, $Z_{i}={\left(Z_{i1},\ldots ,Z_{ip}\right)}^{⊤}∼N\left(0,{\text{Σ}}_{Z}^{\prime }\right)$, ${\text{Σ}}_{Z}^{\prime }={\left(\sigma_{i,j}^{\prime }\right)}_{i,j=1}^{p}$, $\sigma_{i,j}^{\prime }={\left(0.5\right)}^{\left|i-j\right|}$. If i=j or both i and j are ≤20 or both i and j are >20 and $\sigma_{i,j}^{\prime }=0$ otherwise. We make ${\text{Σ}}_{Z}^{\prime }$ block-diagonal for the convenience of obtaining the population solution of β and α through the best logistic or least square approximation under model mis-specifications. In real EHR studies, a paradigm of data generation is that the features X, e.g. some genetic variants, precedes the disease status Y, and Y precedes some clinical surrogate S, e.g. the count of ICD codes associated with the disease. To mimic this scenario, we generated $Y_{i}$ and $S_{i}$ from the following models:

$$\begin{array}{ll} Y_{i}=I\left\{\left(0.8,1,-1,0.8,0.4,0_{p-5}^{⊤}\right)X_{i}+\epsilon_{yi}\geq 0\right\}, & \epsilon_{yi}∼N(0,1), \end{array}$$

$$\begin{array}{ll} S_{i}=\mu Y_{i}+\eta_{1}^{⊤}X_{i}+Y_{i}\eta_{2}^{⊤}X_{i}+\epsilon_{si}, & \epsilon_{si}∼N(0,1). \end{array}$$

Assumptions (${𝓒}^{\text{prior}}$) and (${𝓜}_{S}$) hold when $\eta_{1}=\eta_{2}=0$, and would be severely violated when $\eta_{1}$ and $\eta_{2}$ are large. We design three scenarios with $\eta_{1}$ and $\eta_{2}$ representing different degrees of violation on the surrogate assumptions:

- $\mu =1,\;\text{and}\;\eta_{1}=\eta_{2}=0;$
- $\mu =1.5,\eta_{1}={\left(a_{3}^{⊤},0_{p-5}^{⊤}\right)}^{⊤},\;\text{and}\;\eta_{2}={\left(d_{3}^{⊤},0_{p-5}^{⊤}\right)}^{⊤};$
- $\mu =2,\eta_{1}={\left(a_{3}^{⊤},a_{3}^{⊤},a_{3}^{⊤},0_{p-15}^{⊤}\right)}^{⊤},\;\text{and}\;\eta_{2}={\left(d_{3}^{⊤},d_{3}^{⊤},d_{3}^{⊤},0_{p-15}^{⊤}\right)}^{⊤},$

where $a_{3}={\left(0.6,-0.4,0.4,0.5,-0.5\right)}^{⊤}$ and $d_{3}={\left(0.3,0.4,0.6,-0.5,-0.5\right)}^{⊤}$. Here μ depict the marginal effect of $Y_{i}$ on $S_{i}$, and are set to make the AUC of target models at a similar level across the three scenarios. Across all scenarios, $\text{Pr}\left(Y_{i}=1|S_{i},X_{i}\right)$ is no longer a parametric logistic model, i.e. (${𝓜}_{Y}$) is misspecified. Our goal is to estimate the limiting coefficients $\zeta_{0}$, $\gamma_{0}$, $\beta_{0}$ defined as the minimizor of $E\ell \left(Y_{i},\zeta +\gamma S_{i}+X_{i}^{⊤}\beta \right)$. Benchmark methods, and their implementation, tuning, and evaluation procedures are the same as in Section 4.1, except that we implement supervised LASSO with n ranging from 100 to 700.

**Figure 5:**
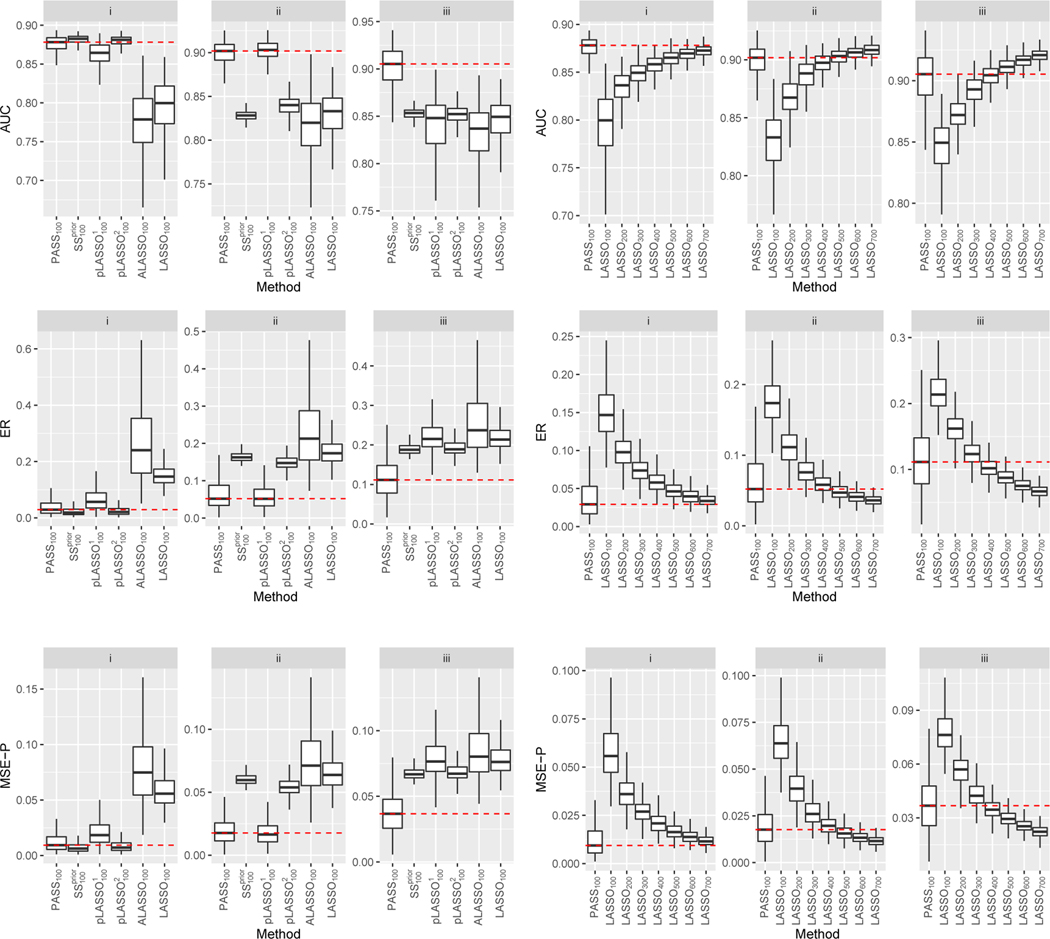
Evaluation metrics on the test set for simulation studies under Scenarios i–iii introduced in Section 4.2. Outliers are not drawn. Mean performance of the PASS approach are marked using red dash lines for ease of comparison. On the left panel, we present the evaluation metrics of all methods for comparison when n=100. On the right panel, we compare the performance of PASS when n=100 with supervised LASSO obtained using labelled samples with various n (from 100 to 700).

In Figure 5, we present AUC, ER and MSE-P of the methods under Scenarios i–iii. In Scenario i, PASS has similar performances as the semi-supervised benchmarks ${\text{SS}}^{\text{prior}}$ and pLASSO, and all the semi-supervised estimators significantly outperform the two supervised estimators since (${𝓒}^{\text{prior}}$) holds and $\alpha^{*}$ basically recovers the direction of $\beta_{0}$ well. Among the semi-supervised estimators, the ${\text{SS}}^{\text{prior}}$ and pLASSO^2^ estimators have a slight advantage with a smaller variation than expected since both heavily rely on the prior information which is of high quality in this setting. In Scenario ii, the key assumption (${𝓒}^{\text{prior}}$) is violated, which drastically impacts the performance of ${\text{SS}}^{\text{prior}}$ and pLASSO^2^. On the other hand, PASS and pLASSO^1^ still effectively leverage the imperfect information from $\alpha^{*}$ to approximately recover the support of $\beta_{0}$, and thus outperform ${\text{SS}}^{\text{prior}}$, pLASSO^2^, and the supervised methods. In Scenario iii, $\eta_{1}$ and $\eta_{2}$ become denser than those in Scenario ii. This can make the recovery of supp ($\beta_{0}$) using supp ($\alpha^{*}$) less accurate, and interestingly, PASS outperforms all methods including pLASSO^1^ that also leverages supp ($\alpha^{*}$). In all the three scenarios, PASS significantly outperforms supervised LASSO using the same number or even 2–3 more times the number of samples, which displays a large gain of using the unlabelled dataset to assist the regression. Finally, the results demonstrate that our method can still efficiently leverage the prior information from S in estimating the target parameters when S∣Y,X highly depends on X so ${𝓒}^{\text{prior}}$ is violated, (${𝓜}_{Y}$) is misspecified, and the design is non-elliptical.

## 5. Application to EHR Phenotying

We examine the performance of PASS along with other approaches in three real world EHR phenotyping studies with the goal of developing classification models for the diseases of interest. All studies are performed at a large tertiary hospital system with EHR spanning over multiple decades. Each study has $n_{0}$ labeled observations for algorithm training and validation. We consider three choices of training size n no more than $n_{0}/2$ in all examples. First, we randomly split the labelled samples into four folds of equal sizes. Then we pick each fold as the validation set, sample n training labels from the other three folds for 20 times, train and validate the algorithms, and finally average the evaluation metrics and their standard errors over the validation results on the four folds. We replicate this procedure 10 times and report the average performance.

### Data Example 1 (CAD Phenotyping).

The goal of this study is to identify patients with coronary artery disease (CAD) based on their EHR features. The study cohort consists of N=4164 patients, out of which a random subset of $n_{0}=181$ patients have their true CAD status annotated via chart review by domain experts. We use the sum of the counts for the CAD ICD code and NLP mention of CAD as the surrogate. There are p=585 additional EHR features consisting of the total count of all ICD codes as a healthcare utilization measure, 10 ICD codes related to CAD, and 574 NLP variables. For the size of training labels, we consider n=50,70,90. This de-identified dataset has been analyzed in previous studies (Zhang et al., 2019, e.g.) and is publicly available online: https://celehs.github.io/PheCAP/articles/example2.html.

### Data Example 2 (RA Phenotyping).

Similar to the CAD phenotyping study, the goal is to identify patients with rheumatoid arthritis (RA) based on their EHR features. There are N=46114 patients in total and out of which, $n_{0}=435$ patients have their RA status annotated. Again, we choose the sum of the ICD code and NLP mention of RA as the surrogate. The p=924 additional EHR features consist of the healthcare utilization and 923 NLP variables potentially predictive of RA. For the size of training labels, we consider n=50,125,200.

### Data Example 3 (Depression Phenotyping).

The goal is to identify patients with depression based on their codified EHR features. There are N=9474 patients in total and $n_{0}=236$ labeled observations. The surrogate is chosen as the counts of depression ICD code. There are p=231 additional EHR features, including the healthcare utilization and 230 codified EHR features on depression related medication prescriptions, laboratory tests and ICD codes. For the size of training labels, we consider 50,85,120.

In the three data examples, N is significantly larger than p with N/max(p,n) being approximately 7 for CAD, 50 for RA, and 41 for Depression. In all the three studies, we apply x→log(1+x) transformation for all count variables. Also, since patients with higher healthcare utilization tend to have higher counts of most features, we orthogonalize all features against the healthcare utilization before regression fitting. Since $\vartheta_{0}$ is unknown in applications, we quantify the performance of an estimator $\tilde{\vartheta }$ based on the AUC and Brier skill score (BSS) of $\sigma \left({\tilde{\vartheta }}^{⊤}W\right)$ for predicting Y, where the BSS is defined as $1-{\hat{E}}_{v}[\{Y-{\sigma \left({\tilde{\vartheta }}^{⊤}W\right)\}}^{2}]/{\hat{E}}_{v}\left[{\left\{Y-{\hat{E}}_{v}(Y)\right\}}^{2}\right]$, and ${\hat{E}}_{v}$ denotes the empirical expectation on the validation sample. The BSS is essentially a binary version of the R-square.

For comparison, we included PASS, ${\text{SS}}^{\text{prior}}$, pLASSO^2^, supervised LASSO and ALASSO on the three data examples to estimate the phenotyping model (${𝓜}_{Y}$). We exclude pLASSO^1^ since it requires fitting of an unpenalized regression on supp ($\hat{\alpha }$), which is infeasible when $|\text{supp}(\hat{\alpha })|>n$. In addition, we compare to the unsupervised LASSO (ULASSO) approach of Chakrabortty et al. (2017), which estimates direction of the logistic coefficients for $Y∼\sigma \left(\beta^{⊤}X\right)$ by regressing $I\left(S>c_{u}\right)$ against X on the subset whose S is either greater than $c_{u}$ or smaller than $c_{l}$, for some pre-specified $c_{u}$ and $c_{l}$ typically chosen such that $\text{Pr}\left(S>c_{u}\right)$ and $\text{Pr}\left(S<c_{l}\right)$ are small. Since the ULASSO approach only provides an estimate $\tilde{\beta }$ to optimize the prediction of $\beta^{⊤}X$ for Y∣X without using S explicitly as an additional predictor, we also derive a semi-supervised variant of ULASSO, denoted by ${\text{SS}}^{\text{ULASSO}}$, by regressing the labeled Y against ${\tilde{\beta }}^{⊤}X$ and S as for ${\text{SS}}^{\text{prior}}$.

**Figure 6:**
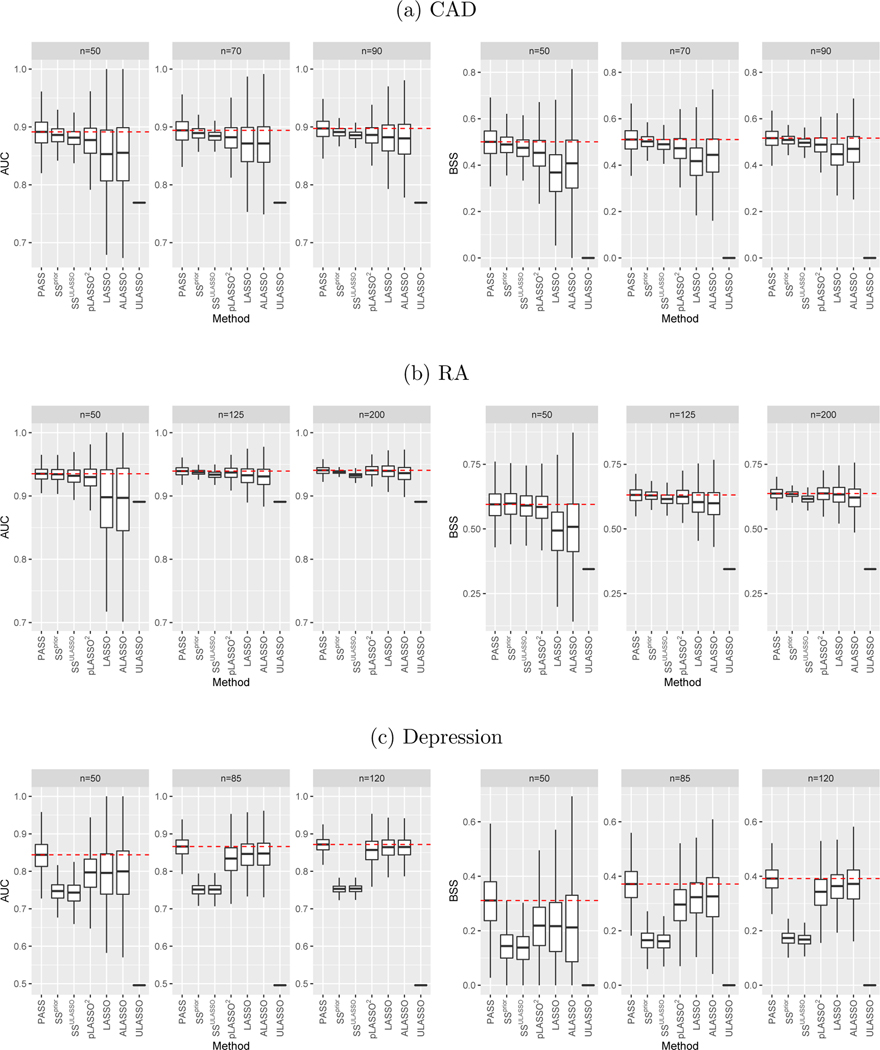
Out of sample AUC and BSS on the data examples 1–3, with various sizes of labelled training samples denoted as n. Median performance of PASS are marked using red dash line for ease of comparison.

As shown in Figure 6, PASS significantly outperforms the supervised LASSO and ALASSO when n=50 in all three examples. As the label size n increases, their performances get closer. Compared with the semi-supervised benchmarks, PASS has slightly or moderately better performance on the CAD and RA studies. For Depression, PASS substantially outperforms them, especially ${\text{SS}}^{\text{prior}}$ and ${\text{SS}}^{\text{ULASSO}}$. For example, when n=50, PASS attained average AUC in classifying depression about 0.1 higher than that of ${\text{SS}}^{\text{prior}}$ and ${\text{SS}}^{\text{ULASSO}}$ and 0.05 higher than pLASSO. The gap becomes smaller when n increases as expected. Interestingly, the supervised estimators outperform pLASSO, ${\text{SS}}^{\text{prior}}$, and ${\text{SS}}^{\text{ULASSO}}$ on the Depression dataset as well but has similar or worse performance than these semi-supervised approaches on the other two examples. This could in part be attributed to the relatively poor quality of the surrogate information, which makes existing semi-supervised approaches fail. In contrast, PASS could utilize such prior information more effectively and robustly, and still preserves better performance than the supervised estimators. Thus, we can conclude that incorporating prior information from the unlabeled dataset could improve and stabilize the prediction performance of phenotyping models in EHR applications, and PASS is more robust and efficient in leveraging the prior information compared with existing semi-supervised methods. In addition, ULASSO shows much worse performance than the other supervised and semi-supervised methods in all examples. This illustrates the importance of collecting labels and including the surrogate in the regression models for EHR phenotyping.

## 6. Discussion

In this paper, we propose PASS, a high dimensional sparse estimator adaptively incorporating the prior knowledge from surrogate under a semi-supervised scenario commonly found in application fields like EHR analysis. Compared to the supervised approaches, the proposed PASS approach can substantially reduce the required number of labeled samples when the model assumptions (${𝓜}_{S}$) and (${𝓒}^{\text{prior}}$) and the elliptical design assumption (C1) hold exactly or approximately, and thus the prior information $\alpha^{*}$ is trustworthy. Compared to existing pLASSO and ${\text{SS}}^{\text{prior}}$ approaches that also incorporates prior information, the PASS approach is robust against unreliable prior information $\alpha^{*}$, which might be the case when the surrogate model assumptions are violated or the design X is highly non-elliptically distributed.

One of the main challenges in our theoretical analysis comes from the colinearity of covariates ${\left(1,S_{i},X_{i}^{⊤}\hat{\alpha },X_{i}^{⊤}\right)}^{⊤}$ due to the enrollment of ρ to leverage the prior information in $\hat{\alpha }$. We overcome this by properly constructing the oracle coefficients $\theta^{*}$ and the restricted eigenvalue assumption (A6). The formulation of our problem falls into the missing data framework with missing completely at random. However, the missing probability approaches 1 as N→∞. This together with the high dimensionality of X makes the theoretical justifications more challenging than those used in the standard missing data literature. Without prior assumptions of $\beta_{0}-\rho \alpha_{0}$ being sparse in certain sense, the unlabeled dataset cannot directly contribute to the estimation of $\beta_{0}$. Our proposed PASS procedure hinges on the sparsity of $\beta_{0}-\rho \alpha_{0}$ to leverage the unlabeled dataset.

We have restricted the discussion to a single surrogate variable for simplicity. However, the proposed method can be easily extended to multiple surrogates. Specifically, consider K surrogates, denoted by $S^{\left[1\right]},\ldots ,S^{\left[\text{K}\right]}$. Let ${\hat{\alpha }}^{\left[\text{k}\right]}$ be the ALASSO estimator regressing $S_{i}^{\left[k\right]}$ against $X_{i}$, $\hat{𝓐}=\cup_{k=1}^{K}\;\text{supp}\left({\hat{\alpha }}_{k}\right)$, $S_{i}={\left(S_{i}^{\left[1\right]},\ldots ,S_{i}^{\left[\text{K}\right]}\right)}^{⊤}$ and $\rho ={\left(\rho^{\left[1\right]},\ldots ,\rho^{\left[\text{K}\right]}\right)}^{⊤}$. We can obtain an estimator for the model parameters as

$$\hat{\zeta },\hat{\gamma },\hat{\rho },\hat{\beta }=\underset{\zeta ,\gamma ,\rho ,\beta }{\arg\min}n^{-1}\sum_{i=1}^{n}\ell \left(Y_{i},\zeta +S_{i}^{⊤}\gamma +X_{i}^{⊤}\beta \right)+\lambda_{1}{\left\|{\left(\beta -\sum_{k}\rho_{k}{\hat{\alpha }}_{k}\right)}_{\hat{𝓐}}\right\|}_{1}+\lambda_{2}{\left\|\beta_{{\hat{𝓐}}^{c}}\right\|}_{1}.$$

Theoretical justification and finite sample performance of $\hat{\beta }$ under this setting warrant further research. In our numerical studies, we only focus on fully simulated datasets and real examples. We are further interested in investigating the performance of our approach through semi-synthetic experiments with various setups for the surrogate variables. In addition, it may be interesting to extend the semi-supervised PASS estimator under a high dimensional sparse parametric regression to semi-parametric settings such as the sparse additive model (Ravikumar et al., 2009) and the sparse varying coefficient model (Noh and Park, 2010). Under semi-parametric models, one could still leverage prior information through shrinking the coefficients to “$\rho \hat{\alpha }$“ with some sparse penalty function, to gain statistical efficiency. Studying the specific forms and theoretical properties of such approaches via a semi-supervised framework warrants future research.

R codes for implementing PASS and the benchmark methods, and replicating the simulation results can be found at https://github.com/moleibobliu/PASS.

## Supplementary Material

Supplement
