# Supplementary material for: Prior Adaptive Semi-supervised Learning with Application to EHR Phenotyping
Source: J Mach Learn Res. Author manuscript; Available in PMC 2023 Nov 15. (PMC10653017)
Supplement: Supplement [file NIHMS1912660-supplement-Supplement.pdf]

Supplement to "Prior Adaptive Semi-supervised Learning with Application to EHR Phenotyping"

June 27, 2023

S1 Proof of Proposition 1

Proof. First, we will show that $S \mid \boldsymbol{X}$ follows a single index model. For any sets $\mathcal{S} \subseteq \mathbb{R}$ and $\mathcal{X} \subseteq \mathbb{R}^p$, we have

$$\Pr(Y = 1 \mid S \in \mathcal{S}, \boldsymbol{X} \in \mathcal{X}) = \frac{\Pr(Y = 1, S \in \mathcal{S}, \boldsymbol{X} \in \mathcal{X})}{\Pr(S \in \mathcal{S}, \boldsymbol{X} \in \mathcal{X})}.$$

By conditional independence,

$$\begin{aligned}
\Pr(S \in \mathcal{S}, \boldsymbol{X} \in \mathcal{X}, Y = y) &= \Pr(S \in \mathcal{S} \mid Y = y) \Pr(\boldsymbol{X} \in \mathcal{X} \mid Y = y) \Pr(Y = y) \\
&= \frac{\Pr(S \in \mathcal{S}, Y = y)}{\Pr(Y = y)} \cdot \frac{\Pr(\boldsymbol{X} \in \mathcal{X}, Y = y)}{\Pr(Y = y)} \cdot \Pr(Y = y) \\
&= \frac{\Pr(S \in \mathcal{S}, Y = y) \Pr(\boldsymbol{X} \in \mathcal{X}, Y = y)}{\Pr(Y = y)}.
\end{aligned}$$

Hence, we have

$$\begin{aligned}
\frac{\Pr(S \in \mathcal{S}, \boldsymbol{X} \in \mathcal{X}, Y = 1)}{\Pr(S \in \mathcal{S}, \boldsymbol{X} \in \mathcal{X}, Y = 0)} &= \frac{\Pr(S \in \mathcal{S}, Y = 1)}{\Pr(S \in \mathcal{S}, Y = 0)} \cdot \frac{\Pr(\boldsymbol{X} \in \mathcal{X}, Y = 1)}{\Pr(\boldsymbol{X} \in \mathcal{X}, Y = 0)} \cdot \frac{\Pr(Y = 0)}{\Pr(Y = 1)} \\
&= \frac{\Pr(Y = 1 \mid S \in \mathcal{S})}{\Pr(Y = 0 \mid S \in \mathcal{S})} \cdot \frac{\Pr(Y = 1 \mid \boldsymbol{X} \in \mathcal{X})}{\Pr(Y = 0 \mid \boldsymbol{X} \in \mathcal{X})} \cdot \frac{\Pr(Y = 0)}{\Pr(Y = 1)}.
\end{aligned}$$

Thus,

$$\begin{aligned}
\operatorname{logit} \Pr(Y = 1 \mid S \in \mathcal{S}, \boldsymbol{X} \in \mathcal{X}) &= \log \frac{\Pr(Y = 1, S \in \mathcal{S}, \boldsymbol{X} \in \mathcal{X})}{\Pr(Y = 0, S \in \mathcal{S}, \boldsymbol{X} \in \mathcal{X})} \\
&= \operatorname{logit} \Pr(Y = 1 \mid S \in \mathcal{S}) + \operatorname{logit} \Pr(Y = 1 \mid \boldsymbol{X} \in \mathcal{X}) \\
&\quad - \operatorname{logit} \Pr(Y = 1).
\end{aligned}$$

Since $\operatorname{logit} \Pr(Y = 1 \mid S \in \mathcal{S}, \boldsymbol{X} \in \mathcal{X})$ depends on $\boldsymbol{X}$ only through $\boldsymbol{X}^\intercal \boldsymbol{\beta}_0$, we conclude that $\operatorname{logit} \Pr(Y = 1 \mid \boldsymbol{X} \in \mathcal{X})$ also depends on $\boldsymbol{X}$ only through $\boldsymbol{X}^\intercal \boldsymbol{\beta}_0$. Namely, $\operatorname{logit} \Pr(Y = 1 \mid \boldsymbol{X} \in \mathcal{X}) = h(\boldsymbol{X}^\intercal \boldsymbol{\beta}_0)$ for some function $h(\cdot)$.

Finally, by conditional independence,

$$
\begin{aligned}
\Pr(S \in \mathcal{S} \mid \boldsymbol{X} \in \mathcal{X}) &= \sum_{y=0,1} \Pr(S \in \mathcal{S}, Y = y \mid \boldsymbol{X} \in \mathcal{X}) \\
&= \sum_{y=0,1} \Pr(S \in \mathcal{S} \mid Y = y, \boldsymbol{X} \in \mathcal{X}) \Pr(Y = y \mid \boldsymbol{X} \in \mathcal{X}) \\
&= \sum_{y=0,1} \Pr(S \in \mathcal{S} \mid Y = y) \Pr(Y = y \mid \boldsymbol{X} \in \mathcal{X}) \\
&= \Pr(S \in \mathcal{S} \mid Y = 1) h(\boldsymbol{X}^\intercal \boldsymbol{\beta}_0) + \Pr(S \in \mathcal{S} \mid Y = 0)\{1 - h(\boldsymbol{X}^\intercal \boldsymbol{\beta}_0)\}.
\end{aligned}
$$

Thus we know that $S \mid \boldsymbol{X}$ follows a single index model $S = \widetilde{f}(\boldsymbol{X}^\intercal \boldsymbol{\beta}_0, \epsilon)$ for some function $\widetilde{f}(\cdot, \cdot)$ and random variable $\epsilon \perp\!\!\!\perp \boldsymbol{X}$. Combining this with $S = f(\boldsymbol{X}^\intercal \boldsymbol{\alpha}_0, \epsilon)$, we conclude that there exists $k_1 \in \mathbb{R}$ such that $\boldsymbol{\alpha}_0 = k_1 \boldsymbol{\beta}_0$.

Next, we will show that least squares estimation recovers the direction of $\boldsymbol{\beta}_0$. This step is of the same nature as that of Li and Duan (1989, Theorem 2.1). Without loss of generality, we assume $\mathbb{E}[\boldsymbol{X}] = \boldsymbol{0}$. Let $\tau^*, \boldsymbol{\alpha}^* = \arg\min_{\tau, \boldsymbol{\alpha}} \mathbb{E}(S - \tau - \boldsymbol{X}^\intercal \boldsymbol{\alpha})^2$. Then

$$
\begin{aligned}
\mathbb{E}(S - \tau^* - \boldsymbol{X}^\intercal \boldsymbol{\alpha}^*)^2 &= \mathbb{E}\{\widetilde{f}(\boldsymbol{X}^\intercal \boldsymbol{\beta}_0, \epsilon) - \tau^* - \boldsymbol{X}^\intercal \boldsymbol{\alpha}^*\}^2 \\
&= \mathbb{E}\left(\mathbb{E}[\{\widetilde{f}(\boldsymbol{X}^\intercal \boldsymbol{\beta}_0, \epsilon) - \tau^* - \boldsymbol{X}^\intercal \boldsymbol{\alpha}^*\}^2 \mid \boldsymbol{X}^\intercal \boldsymbol{\beta}_0, \epsilon]\right) \\
&\geq \mathbb{E}\left\{\widetilde{f}(\boldsymbol{X}^\intercal \boldsymbol{\beta}_0, \epsilon) - \tau^* - \mathbb{E}(\boldsymbol{X}^\intercal \boldsymbol{\alpha}^* \mid \boldsymbol{X}^\intercal \boldsymbol{\beta}_0)\right\}^2
\end{aligned}
$$

using Jensen's inequality and the independence between $\boldsymbol{X}$ and ϵ. By the linearity condition, $\mathbb{E}(\boldsymbol{X}^\intercal \boldsymbol{\alpha}^* \mid \boldsymbol{X}^\intercal \boldsymbol{\beta}_0) = c_1 + k_2 \boldsymbol{X}^\intercal \boldsymbol{\beta}_0$ for some constants c_1, k_2. Since $\mathbb{E}[\boldsymbol{X}] = \boldsymbol{0}$, we have $c_1 = 0$. Hence, we have

$$\mathbb{E}(S - \tau^* - \boldsymbol{X}^\intercal \boldsymbol{\alpha}^*)^2 \geq \mathbb{E}(S - \tau^* - k_2 \boldsymbol{X}^\intercal \boldsymbol{\beta}_0)^2.$$

Also, since $\tau^*, \boldsymbol{\alpha}^* = \arg\min_{\tau, \boldsymbol{\alpha}} \mathbb{E}(S - \tau - \boldsymbol{X}^\intercal \boldsymbol{\alpha})^2$, we have

$$\mathbb{E}(S - \tau^* - \boldsymbol{X}^\intercal \boldsymbol{\alpha}^*)^2 \leq \mathbb{E}(S - \tau^* - k_2 \boldsymbol{X}^\intercal \boldsymbol{\beta}_0)^2.$$

Thus, we have

$$\mathbb{E}(S - \tau^* - \boldsymbol{X}^\intercal \boldsymbol{\alpha}^*)^2 = \mathbb{E}(S - \tau^* - k_2 \boldsymbol{X}^\intercal \boldsymbol{\beta}_0)^2. \tag{S1}$$

Since $\mathbb{E}(\boldsymbol{X}\boldsymbol{X}^\intercal)$ is positive–definite, the square loss function $\mathbb{E}(S - \tau - \boldsymbol{X}^\intercal \boldsymbol{\alpha})^2$ is strongly convex. So its minimizer $(\tau^*, \boldsymbol{\alpha}^*)$ is unique, which combined with (S1) indicates that $\boldsymbol{\alpha}^* = k_2 \boldsymbol{\beta}_0$.

□

S2 Assumptions and Proof of Theorem 1

S2.1 Technical assumptions

We first present the technical assumptions required in Theorem 1. Below each assumption we also provide a comment on its feasibility.

(A1) (Sub-Gaussian predictors and noise) *There exists a constant B such that each predictor U_j and the error term $S - \boldsymbol{U}^\intercal \boldsymbol{\xi}^*$ are sub-Gaussian(B^2).*

Assumption (A1) regularize the tail of predictors and residuals and has been commonly used in the literature (e.g. van de Geer and Bühlmann, 2009; Bickel et al., 2009).

(A2) (Control over extreme probabilities) *There exists a positive constant ϖ such that $\boldsymbol{v}^\intercal \boldsymbol{H} \boldsymbol{v} \geq \varpi \cdot \boldsymbol{v}^\intercal \boldsymbol{G} \boldsymbol{v}$ for all $\boldsymbol{v} \in \mathbb{R}^{p+3}$.*

(A2) implicitly limits the magnitude of $\boldsymbol{\theta}^*$ and ensures that the distribution of $\boldsymbol{Z}_{\boldsymbol{\alpha}^*} \mid Y = 1$ has sufficient overlap with that of $\boldsymbol{Z}_{\boldsymbol{\alpha}^*} \mid Y = 0$. This assumption is weaker than the more common assumption that the probability $\sigma(\boldsymbol{Z}_{\boldsymbol{\alpha}^*}^\intercal \boldsymbol{\theta}_0)$ are bounded away from 0 and 1 almost surely, and is more suitable for EHR applications where extreme probabilities often occur.

(A3) (Nonlinear impact) *Let ϖ be as defined in (A2). There exists a positive constant η such that $15\varpi^{3/2}(\mathbb{E}|\boldsymbol{Z}_{\boldsymbol{\alpha}^*}^\intercal \boldsymbol{v}|^2)^{3/2} \geq \eta \cdot \mathbb{E}|\boldsymbol{Z}_{\boldsymbol{\alpha}^*}^\intercal \boldsymbol{v}|^3$ for all $\boldsymbol{v} \in \mathbb{R}^{p+3}$.*

(A3) assumes the negative log-likelihood can be well approximated by a quadratic function in the neighborhood of $\boldsymbol{\theta}_0$. It was first introduced by Belloni and Chernozhukov (2011). This assumption is not needed if $\sigma(\boldsymbol{Z}_{\boldsymbol{\alpha}^*}^\intercal \boldsymbol{\theta}_0)$ is almost surely bounded away from 0 and 1. Furthermore, it holds if $\boldsymbol{Z}_{\boldsymbol{\alpha}^*}$ are Gaussian or bounded.

(A4) (Restricted eigenvalue and general position for $\boldsymbol{K}$) *There exists a positive constant φ such that $\boldsymbol{v}^\intercal \boldsymbol{K} \boldsymbol{v} \geq \varphi \cdot \boldsymbol{v}_{\mathcal{Q}^*}^\intercal \boldsymbol{v}_{\mathcal{Q}^*}$ for all $\boldsymbol{v} \in \mathscr{C}$, where $\mathscr{C} = \{\boldsymbol{v} \in \mathbb{R}^{p+1} : \|\boldsymbol{v}_{\mathcal{Q}^{*c}}\|_1 \leq 3\|\boldsymbol{v}_{\mathcal{Q}^*}\|_1\}$ and $\mathcal{Q}^* = \mathrm{supp}(\boldsymbol{\alpha}^*) \cup \{p+1\}$. In addition, columns of $\boldsymbol{K}$ are in general position, i.e., for any $\ell < \min\{N, p\}$, there exists no ℓ-dimensional subspace of $\boldsymbol{K}$'s columns containing more than $\ell + 1$ columns belonging to $\boldsymbol{K}$ or $-\boldsymbol{K}$.*

(A4) imposes regularity conditions on the design $\boldsymbol{K}$. The restricted eigenvalue condition ensures that $(\widehat{\boldsymbol{\alpha}} - \boldsymbol{\alpha}^*)^\intercal \boldsymbol{K} (\widehat{\boldsymbol{\alpha}} - \boldsymbol{\alpha}^*)$ is bounded below by $\|\widehat{\boldsymbol{\alpha}} - \boldsymbol{\alpha}^*\|_2^2$ (e.g. van de Geer and Bühlmann, 2009; Bickel et al., 2009). The general position condition is used to ensure the uniqueness of the solution $\widehat{\tau}, \widehat{\boldsymbol{\alpha}}$ (Tibshirani et al., 2013). When $N > p$ and the correlation structure of $\boldsymbol{U}$ is non-degenerate, this condition is satisfied.

(A5) (Minimum signal strength in $\boldsymbol{\alpha}^*$) *Let $\mathcal{A}^* = \mathrm{supp}(\boldsymbol{\alpha}^*)$, $\alpha_{min}^* = \min_{j \in \mathcal{A}^*} |\alpha_j^*|$ and $q^* = |\mathcal{A}^*| + 1$. We have $\alpha_{min}^* \geq 128 B^2 \{\log(p/\epsilon)/N\}^{1/2} q^*/\varphi^2$ where B is introduced in (A1).*

Similar minimum signal strength assumption to (A5) can be found at Zou and Zhang (2009). One may note that (A5) is essentially stronger than those in the LASSO literature like Zhao and Yu (2006). In comparison with Zhao and Yu (2006), we impose essentially weaker restriction on the correlation structure of the design matrix. In many practical settings such as typical EHR applications, (A5) is easily fulfilled as N is very large. Both assumptions (A4) and (A5) are used to establish estimation and variable selection consistency of $\widehat{\boldsymbol{\alpha}}$.

(A6) (Restricted eigenvalue for $\boldsymbol{G}$) *For any $\mathcal{S}_+$ satisfying $\mathcal{S}_+ \cap \mathcal{A}^* = \emptyset$, there exists a constant $\phi > 0$ such that $\boldsymbol{v}^\intercal \boldsymbol{G} \boldsymbol{v} \geq \phi \cdot \boldsymbol{v}_{\mathcal{S}_+^*}^\intercal \boldsymbol{v}_{\mathcal{S}_+^*}$ for all $\boldsymbol{v}$ satisfying $\Omega(\boldsymbol{v}_{\mathcal{P} \setminus \mathcal{S}_+^*}) \leq 3\Omega(\boldsymbol{v}_{\mathcal{S}_+^*})$.*

Similarly to (A4), this assumption regularizes the design matrix $\boldsymbol{Z}_{\boldsymbol{\alpha}^*} = (\boldsymbol{X}^\intercal, \boldsymbol{X}^\intercal \boldsymbol{\alpha}^*, S, 1)^\intercal$. One should note that the constant ϕ in Assumption (A6) is not impacted by the

collinearity between $\boldsymbol{X}^\intercal$ and $\boldsymbol{X}^\intercal\boldsymbol{\alpha}^*$ since the subset of predictors corresponding to $\mathcal{S}_+$ is non-singular and we have ruled that $\mathcal{S}_+ \cap \mathcal{A}^* = \emptyset$.

(A7) (Tuning parameters) *Let q^* be as defined in (A5). The tuning parameters for $\widehat{\boldsymbol{\alpha}}$ satisfy $\mu_{init} \leq \alpha^*_{min}\varphi^2/(16q^*)$, $\mu_{init} \geq 8B^2\{\log(p/\epsilon)/N\}^{1/2}$, $\mu \leq 2^{-\nu-1}(\alpha^*_{min})^\nu\mu_{init}$, and $\mu \geq 8^\nu(q^*/\varphi^2)^\nu\{1 + 8B(q^*/\varphi^2)^{1/2}\}\mu_{init}^{\nu+1}$. The tuning parameters for $\widehat{\boldsymbol{\theta}}$ satisfy $\lambda_1 \geq 36B\{\log(2q^*/\epsilon)/n\}^{1/2}$, and $\lambda_2 \geq 36B\{\log(2p/\epsilon)/n\}^{1/2}$.*

(A7) specifies the ranges for tuning parameters. The order of the tuning parameters are similar to those in the LASSO literature (see, e.g. van de Geer and Bühlmann, 2009; Bickel et al., 2009). Existence of the tuning parameters μ and μ_{init} in (A7) is guaranteed by (A5). Also note that we take $\lambda_1 \asymp \{\log(q^*)/n\}^{1/2} \ll \lambda_2 \asymp \{\log(p)/n\}^{1/2}$ since the fitted support $\widehat{\mathcal{A}}$ recovers $\mathcal{A}^*$ satisfying $q^* = |\mathcal{A}^*| + 1$ and thus λ_1 only needs to control the tail behaviour of q^* empirical errors. This plays an essential role in reducing the excess risk of the PASS estimator when the $\boldsymbol{\alpha}^*$ recovers the direction or support of $\boldsymbol{\beta}_0$.

(A8) (Sample sizes) *The sample size N satisfies $384B^2\{\log(p/\epsilon)/N\}^{1/2}(q^*)/\varphi^2 \leq 1$ and $2\log(p/\epsilon)/N \leq 1$. The oracle loss function satisfies $64\mathscr{E}(\boldsymbol{\theta}^*, \mathcal{S}^*_+, \mathcal{S}^*_-) \leq \eta$.*

(A8) regularizes the growth rate of N, n, p and q^*. The condition $64\mathscr{E}(\boldsymbol{\theta}^*, \mathcal{S}^*_+, \mathcal{S}^*_-) \leq \eta$ holds as long as $n^{-1}\log(p)|\mathcal{B}_0| \to 0$, under the choices of the parameters in Cases 1–3 presented in Section 3.2. This means p can grow exponentially fast compared to n. In the justification of Theorem 1, this condition is used to ensure that $\widehat{\boldsymbol{\theta}}$ falls into a neighborhood of $\boldsymbol{\theta}^*$ and $\boldsymbol{\theta}_0$ where the negative log-likelihood can be well approximated by a quadratic function.

S2.2 Overview

The proof of Theorem 1 is lengthy. Hence we will provide an overview of the steps in the proof before going into the details. In the following we shall fix an arbitrary $\epsilon > 0$.

Step 1 The first step of the proof establishes the convergence rate of $\widehat{\boldsymbol{\alpha}}$. We will give an upper bound on $\|\widehat{\boldsymbol{\alpha}} - \boldsymbol{\alpha}^*\|_1$ and show that the support of $\widehat{\boldsymbol{\alpha}}$ is identical to that of $\boldsymbol{\alpha}^*$ with high probability. This step is necessary because the proposed estimator $\widehat{\boldsymbol{\beta}}$ builds upon $\widehat{\boldsymbol{\alpha}}$.

As in the main text, let $\boldsymbol{U} = (\boldsymbol{X}^\intercal, 1)^\intercal$ and $\boldsymbol{\xi} = (\boldsymbol{\alpha}^\intercal, \tau)^\intercal$. Then we have

$$\widehat{\boldsymbol{\xi}}_{\text{init}} = (\widehat{\boldsymbol{\alpha}}^\intercal_{\text{init}}, \widehat{\tau}_{\text{init}})^\intercal = \arg\min_{\boldsymbol{\xi}} \mathbb{P}_N(S - \boldsymbol{U}^\intercal\boldsymbol{\xi})^2 + \mu_{\text{init}}\|\boldsymbol{\xi}_{\mathcal{P}}\|_1,$$

and

$$\widehat{\boldsymbol{\xi}} = (\widehat{\boldsymbol{\alpha}}^\intercal, \widehat{\tau})^\intercal = \arg\min_{\boldsymbol{\xi}} \mathbb{P}_N(S - \boldsymbol{U}^\intercal\boldsymbol{\xi})^2 + \mu\sum_{j=1}^{p+1}\widehat{\omega}_j|\xi_j|,$$

where $\mathcal{P} = \{1, \ldots, p\}$, $\widehat{\omega}_j = |\widehat{\xi}_{\text{init},j}|^{-\nu} = |\widehat{\alpha}_{\text{init},j}|^{-\nu}$ when $j \in \mathcal{P}$ and $\widehat{\omega}_j = 0$ when $j \notin \mathcal{P}$. Define the limiting quantity as $\boldsymbol{\xi}^* = \arg\min_{\boldsymbol{\xi}} \mathbb{E}(S - \boldsymbol{U}^\intercal\boldsymbol{\xi})^2$. Let $\mathcal{Q}^* = \text{supp}(\boldsymbol{\xi}^*) \cup \{p+1\}$ and $q^* = |\mathcal{Q}^*|$.

In Section 2.2 we prove the risk bounds for $\widehat{\boldsymbol{\xi}}_{\text{init}}$ and $\widehat{\boldsymbol{\xi}}$. Specifically, we deal with $\widehat{\boldsymbol{\xi}}_{\text{init}}$ in Section 2.2.1 and $\widehat{\boldsymbol{\xi}}$ in Section 2.2.2, while the proofs of some auxiliary lemmas are collected in Section 2.2.3. The convergence rate of $\widehat{\boldsymbol{\alpha}}$ follows easily from the risk bound of $\widehat{\boldsymbol{\xi}}$. And we will also show that the support of $\widehat{\boldsymbol{\alpha}}$ is identical to that of $\boldsymbol{\alpha}^*$ with high probability. The properties about $\widehat{\boldsymbol{\alpha}}$ is summarized in Lemma S8.

Step 2 The second step of the proof concerns substituting $\widehat{\boldsymbol{\alpha}}$ by $\boldsymbol{\alpha}^*$ in the construction of $\widehat{\boldsymbol{\beta}}$ and bounding the error due to this substitution. Then we will relate excess risk and prediction error to a quadratic form involving $\boldsymbol{H}$. Recall that $\boldsymbol{H} = \mathbb{E}\left[\sigma(\boldsymbol{Z}_{\boldsymbol{\alpha}^*}^\intercal\boldsymbol{\theta}_0)\{1-\sigma(\boldsymbol{Z}_{\boldsymbol{\alpha}^*}^\intercal\boldsymbol{\theta}_0)\}\boldsymbol{Z}_{\boldsymbol{\alpha}^*}\boldsymbol{Z}_{\boldsymbol{\alpha}^*}^\intercal\right]$ is defined in the main text.

For any $\boldsymbol{\theta} = (\boldsymbol{\delta}^\intercal, \rho, \gamma, \zeta)^\intercal$, define

$$\widehat{\Psi}_+(\boldsymbol{\theta}) = (\boldsymbol{\delta}^\intercal + \rho(\widehat{\boldsymbol{\alpha}} - \boldsymbol{\alpha}^*)^\intercal, \rho, \gamma, \zeta)^\intercal,$$
$$\widehat{\Psi}_-(\boldsymbol{\theta}) = (\boldsymbol{\delta}^\intercal - \rho(\widehat{\boldsymbol{\alpha}} - \boldsymbol{\alpha}^*)^\intercal, \rho, \gamma, \zeta)^\intercal.$$

Then $\boldsymbol{Z}_{\widehat{\boldsymbol{\alpha}}}^\intercal\boldsymbol{\theta} = \boldsymbol{Z}_{\boldsymbol{\alpha}^*}^\intercal\widehat{\Psi}_+(\boldsymbol{\theta})$ and $\boldsymbol{Z}_{\boldsymbol{\alpha}^*}^\intercal\boldsymbol{\theta} = \boldsymbol{Z}_{\widehat{\boldsymbol{\alpha}}}^\intercal\widehat{\Psi}_-(\boldsymbol{\theta})$ for any $\boldsymbol{\theta}$. Moreover, $\widehat{\Psi}_+(\widehat{\Psi}_-(\boldsymbol{\theta})) = \boldsymbol{\theta}$ and $\widehat{\Psi}_-(\widehat{\Psi}_+(\boldsymbol{\theta})) = \boldsymbol{\theta}$ for any $\boldsymbol{\theta}$. Note that both $\widehat{\Psi}_+$ and $\widehat{\Psi}_-$ are linear in $\boldsymbol{\theta}$. The proposed estimator for $\boldsymbol{\theta}_0$ is

$$\widehat{\boldsymbol{\theta}} = \arg\min_{\boldsymbol{\theta}} \mathbb{P}_n\,\ell(Y, \boldsymbol{Z}_{\widehat{\boldsymbol{\alpha}}}^\intercal\boldsymbol{\theta}) + \lambda_1\|\boldsymbol{\theta}_{\widehat{\mathcal{A}}}\|_1 + \lambda_2\|\boldsymbol{\theta}_{\mathcal{P}\setminus\widehat{\mathcal{A}}}\|_1.$$

With high probability, $\widehat{\mathcal{A}} = \mathcal{A}^*$ which is the support of $\boldsymbol{\alpha}^*$. Hence, with high probability

$$\widehat{\boldsymbol{\theta}} = \arg\min_{\boldsymbol{\theta}} \mathbb{P}_n\,\ell(Y, \boldsymbol{Z}_{\boldsymbol{\alpha}^*}^\intercal\widehat{\Psi}_+(\boldsymbol{\theta})) + \lambda_1\|\boldsymbol{\theta}_{\mathcal{A}^*}\|_1 + \lambda_2\|\boldsymbol{\theta}_{\mathcal{P}\setminus\mathcal{A}^*}\|_1.$$

Define $L_n(\boldsymbol{\theta}) = \mathbb{P}_n\,\ell(Y, \boldsymbol{Z}_{\boldsymbol{\alpha}^*}^\intercal\boldsymbol{\theta})$ and $L_0(\boldsymbol{\theta}) = \mathbb{E}\,\ell(Y, \boldsymbol{Z}_{\boldsymbol{\alpha}^*}^\intercal\boldsymbol{\theta})$. With the notations of $\widehat{\Psi}_+(\cdot)$ and $\widehat{\Psi}_-(\cdot)$, we can write the excess risk as

$$\mathbb{E}\,\ell(Y, \boldsymbol{Z}_{\widehat{\boldsymbol{\alpha}}}^\intercal\widehat{\boldsymbol{\theta}}) - \mathbb{E}\,\ell(Y, \boldsymbol{Z}_{\boldsymbol{\alpha}^*}^\intercal\boldsymbol{\theta}_0) = \mathbb{E}\,\ell(Y, \boldsymbol{Z}_{\boldsymbol{\alpha}^*}^\intercal\widehat{\Psi}_+(\widehat{\boldsymbol{\theta}})) - \mathbb{E}\,\ell(Y, \boldsymbol{Z}_{\boldsymbol{\alpha}^*}^\intercal\boldsymbol{\theta}_0) = L_0(\widehat{\Psi}_+(\widehat{\boldsymbol{\theta}})) - L_0(\boldsymbol{\theta}_0)$$

Similarly, the linear prediction error can be written as

$$\mathbb{E}(\boldsymbol{Z}_{\widehat{\boldsymbol{\alpha}}}^\intercal\widehat{\boldsymbol{\theta}} - \boldsymbol{Z}_{\boldsymbol{\alpha}^*}^\intercal\boldsymbol{\theta}_0)^2 = \mathbb{E}(\boldsymbol{Z}_{\boldsymbol{\alpha}^*}^\intercal\widehat{\Psi}_+(\widehat{\boldsymbol{\theta}}) - \boldsymbol{Z}_{\boldsymbol{\alpha}^*}^\intercal\boldsymbol{\theta}_0)^2$$

and the probability prediction error can be written as

$$\mathbb{E}\{\sigma(\boldsymbol{Z}_{\widehat{\boldsymbol{\alpha}}}^\intercal\widehat{\boldsymbol{\theta}}) - \sigma(\boldsymbol{Z}_{\boldsymbol{\alpha}^*}^\intercal\boldsymbol{\theta}_0)\}^2 = \mathbb{E}\{\sigma(\boldsymbol{Z}_{\boldsymbol{\alpha}^*}^\intercal\widehat{\Psi}_+(\widehat{\boldsymbol{\theta}})) - \sigma(\boldsymbol{Z}_{\boldsymbol{\alpha}^*}^\intercal\boldsymbol{\theta}_0)\}^2,$$

where $\sigma(t) = e^t/(1+e^t)$. All the three quantities can be expressed in terms of $\widehat{\Psi}_+(\widehat{\boldsymbol{\theta}})$.

In Section 2.3.1, we show that the expected log-likelihood can be approximated by a quadratic function near $\boldsymbol{\theta}_0$. In Section 2.3.2, we will show that the excess risk, the linear prediction error and the probability prediction error are all related to an important quantity $\{\widehat{\Psi}_+(\widehat{\boldsymbol{\theta}}) - \boldsymbol{\theta}_0\}^T\boldsymbol{H}\{\widehat{\Psi}_+(\widehat{\boldsymbol{\theta}}) - \boldsymbol{\theta}_0\}$.

Step 3 The third step of the proof is about a careful analysis of the quantity $\{\widehat{\Psi}_+(\widehat{\boldsymbol{\theta}}) - \boldsymbol{\theta}_0\}^T \boldsymbol{H}\{\widehat{\Psi}_+(\widehat{\boldsymbol{\theta}}) - \boldsymbol{\theta}_0\} = \|\boldsymbol{H}^{1/2}(\widehat{\Psi}_+(\widehat{\boldsymbol{\theta}}) - \boldsymbol{\theta}_0)\|_2^2$. The key step is to pick an oracle $\boldsymbol{\theta}^*$ and decompose $\widehat{\Psi}_+(\widehat{\boldsymbol{\theta}}) - \boldsymbol{\theta}_0$ as $(\widehat{\Psi}_+(\widehat{\boldsymbol{\theta}}) - \boldsymbol{\theta}^*) + (\boldsymbol{\theta}^* - \boldsymbol{\theta}_0)$. Here we recall the definition of the oracle $\boldsymbol{\theta}^*$. With $\boldsymbol{\theta} = (\boldsymbol{\delta}^\intercal, \rho, \gamma, \zeta)^\intercal$, define $\Omega(\boldsymbol{\theta}) = \lambda_0(|\rho| + |\gamma| + |\zeta|) + \lambda_1\|\boldsymbol{\delta}_{\mathcal{A}^*}\|_1 + \lambda_2\|\boldsymbol{\delta}_{\mathcal{P}\setminus\mathcal{A}^*}\|_1$ and $\Pi(\boldsymbol{\theta}) = |\rho|$, where $\lambda_0 = 36B\{\log(6/\epsilon)/n\}^{1/2}$. Define

$$\begin{aligned}\mathscr{E}(\boldsymbol{\theta}, \mathcal{S}_+, \mathcal{S}_-) =& \,\mathbb{E}\,\ell(Y, \boldsymbol{Z}_{\boldsymbol{\alpha}^*}^\intercal\boldsymbol{\theta}) - \mathbb{E}\,\ell(Y, \boldsymbol{Z}_{\boldsymbol{\alpha}^*}^\intercal\boldsymbol{\theta}_0)\\ &+ 256\frac{\kappa(\mathcal{S}_+)^2|\mathcal{S}_+|}{\varpi\psi(\mathcal{S}_+)} + 8\lambda_1\|\boldsymbol{\theta}_{\mathcal{S}_-\cap\mathcal{A}^*}\|_1 + 8\lambda_2\|\boldsymbol{\theta}_{\mathcal{S}_-\cap(\mathcal{P}\setminus\mathcal{A}^*)}\|_1 + 8\lambda_1\Delta_{\boldsymbol{\alpha}}\Pi(\boldsymbol{\theta}),\end{aligned} \tag{S2}$$

where

$$\psi(\mathcal{S}_+) = \inf_{\boldsymbol{v}:\Omega(\boldsymbol{v}_{\mathcal{S}_-})\le 3\Omega(\boldsymbol{v}_{\mathcal{S}_+})} \frac{\boldsymbol{v}^\intercal\boldsymbol{G}\boldsymbol{v}}{\boldsymbol{v}_{\mathcal{S}_+}^\intercal\boldsymbol{v}_{\mathcal{S}_+}},$$

$$\kappa(\mathcal{S}_+) = \begin{cases}\lambda_0, & \text{if } \mathcal{S}_+\cap\mathcal{A}^* = \emptyset \text{ and } \mathcal{S}_+\cap(\mathcal{P}\setminus\mathcal{A}^*) = \emptyset\\ \lambda_2, & \text{if } \mathcal{S}_+\cap\mathcal{A}^* = \emptyset \text{ and } \mathcal{S}_+\cap(\mathcal{P}\setminus\mathcal{A}^*) \ne \emptyset\\ +\infty, & \text{if } \mathcal{S}_+\cap\mathcal{A}^* \ne \emptyset\end{cases}.$$

Define $\boldsymbol{\theta}^* = (\boldsymbol{\delta}^{*\intercal}, \rho^*, \gamma^*, \zeta^*)^\intercal$, $\mathcal{S}_+^*$ and $\mathcal{S}_-^*$ as the solution to

$$\mathop{\arg\min}_{\{\boldsymbol{\theta},\mathcal{S}_+,\mathcal{S}_-\}:\ \mathcal{S}_+\cap\mathcal{S}_-=\emptyset,\ \mathcal{S}_+\cup\mathcal{S}_-=\mathrm{supp}(\boldsymbol{\theta})\cup\overline{\mathcal{P}},\ \mathcal{S}_+\supseteq\overline{\mathcal{P}},\ \text{and}\ \|\boldsymbol{G}^{1/2}(\boldsymbol{\theta}-\boldsymbol{\theta}_0)\|_2\le\eta} \mathscr{E}(\boldsymbol{\theta}, \mathcal{S}_+, \mathcal{S}_-)$$

where $\overline{\mathcal{P}} = \{p+1, p+2, p+3\}$. Let $\mathcal{S}^* = \mathcal{S}_+^* \cup \mathcal{S}_-^* = \mathrm{supp}(\boldsymbol{\theta}^*)$ and $\kappa^* = \kappa(\mathcal{S}_+^*)$.

The ultimate goal is to show that $\{\widehat{\Psi}_+(\widehat{\boldsymbol{\theta}}) - \boldsymbol{\theta}_0\}^T \boldsymbol{H}\{\widehat{\Psi}_+(\widehat{\boldsymbol{\theta}}) - \boldsymbol{\theta}_0\} \le D^2$, where $D^2 = 64\mathscr{E}(\boldsymbol{\theta}^*, \mathcal{S}_+^*, \mathcal{S}_-^*)$. Details are given in Section 2.4. Specifically, we state a few auxiliary lemmas and prove the aforementioned inequality and Theorem 1 in the main text in Section 2.4.1. The proofs of those auxiliary lemmas are collected in Section S2.6.

S2.3 Details About Step 1

We shall state a few lemmas. The proofs of all technical lemmas are deferred to Section S2.6. Define the mean squared error of the prediction as $\mathscr{E}_1(\boldsymbol{\xi}) = \mathbb{E}(\boldsymbol{U}^\intercal\boldsymbol{\xi} - \boldsymbol{U}^\intercal\boldsymbol{\xi}^*)^2$, where $\boldsymbol{\xi}^* = \arg\min_{\boldsymbol{\xi}} \mathbb{E}(S - \boldsymbol{U}^\intercal\boldsymbol{\xi})^2$. The following lemma gives some basic properties about $\boldsymbol{\xi}^*$ and $\mathscr{E}_1(\boldsymbol{\xi})$.

Lemma S1. *We have* $\mathbb{E}\{U_j(S - \boldsymbol{U}^\intercal\boldsymbol{\xi}^*)\} = 0$ *for all* j. *Also, for any* $\boldsymbol{\xi}$ *it holds that* $\mathscr{E}_1(\boldsymbol{\xi}) = \mathbb{E}(S - \boldsymbol{U}^\intercal\boldsymbol{\xi})^2 - \mathbb{E}(S - \boldsymbol{U}^\intercal\boldsymbol{\xi}^*)^2$.

Define the events

$$\begin{aligned}\mathscr{T}_1 &= \left\{|\mathbb{P}_N U_j(S - \boldsymbol{U}^\intercal\boldsymbol{\xi}^*)| \le 8B^2\Big\{\frac{\log(p/\epsilon)}{N}\Big\}^{1/2} \text{ for all } j\right\},\\ \mathscr{T}_2 &= \left\{\inf_{\boldsymbol{\xi}:\|\boldsymbol{\xi}_{\mathcal{Q}^{*c}}\|_1\le 3\|\boldsymbol{\xi}_{\mathcal{Q}^*}\|_1, \mathbb{E}(\boldsymbol{U}^\intercal\boldsymbol{\xi})^2=1}\{\mathbb{P}_N(\boldsymbol{U}^\intercal\boldsymbol{\xi})^2 - 0.5\,\mathbb{E}(\boldsymbol{U}^\intercal\boldsymbol{\xi})^2\} \ge 0\right\}, \quad \text{and}\\ \mathscr{T}_3 &= \big\{|\mathbb{P}_N U_j^2| \le 16B^2 \text{ for all } j\big\}.\end{aligned}$$

The following lemmas show that, under certain conditions all these events happen with large probability.

Lemma S2. *If the assumption A8 holds, then* $\Pr(\mathscr{T}_1) \geq 1 - \epsilon$.

Lemma S3. *If the assumptions A4 and A8 hold, then* $\Pr(\mathscr{T}_2) \geq 1 - \epsilon$.

Lemma S4. *If the assumption A8 holds, then* $\Pr(\mathscr{T}_3) \geq 1 - \epsilon$.

S2.3.1 Properties of $\widehat{\boldsymbol{\xi}}_{\text{init}}$

Recall that the initial lasso estimator $\widehat{\boldsymbol{\xi}}_{\text{init}}$ satisfies

$$\widehat{\boldsymbol{\xi}}_{\text{init}} = \arg\min_{\boldsymbol{\xi}} \mathbb{P}_N(S - \boldsymbol{U}^\intercal \boldsymbol{\xi})^2 + \mu_{\text{init}} \|\boldsymbol{\xi}_{\mathcal{P}}\|_1.$$

Lemma S5. *Under assumptions A1 – A8, on the event* $\mathscr{T}_1 \cap \mathscr{T}_2 \cap \mathscr{T}_3$*, we have* $\|\widehat{\boldsymbol{\xi}}_{init} - \boldsymbol{\xi}^*\|_1 \leq 8\mu_{init} q^*/\varphi^2$.

S2.3.2 Properties of $\widehat{\boldsymbol{\xi}}$

Recall that $\widehat{\omega}_j = |(\widehat{\xi}_{\text{init}})_j|^{-\nu}$ for $j \in \mathcal{P}$ and $\widehat{\omega}_j = 0$ when $j \notin \mathcal{P}$, where $\mathcal{P} = \{1, \ldots, p\}$. Let $\xi^*_{\min} = \min_{j \in \mathcal{Q}^* \cap \mathcal{P}} |\xi^*_j|$. By definition, we know $\xi^*_{\min} = \alpha^*_{\min}$. Define

$$\widetilde{\boldsymbol{\xi}} = \mathop{\arg\min}_{\boldsymbol{\xi}:\, \boldsymbol{\xi}_{\mathcal{Q}^{*c}} = \boldsymbol{0}} \mathbb{P}_N(S - \boldsymbol{U}^\intercal \boldsymbol{\xi})^2 + \mu \sum_{j \in \mathcal{Q}^*} \widehat{\omega}_j |\xi_j|.$$

Lemma S6. *Under assumptions A1 – A8, on the event* $\mathscr{T}_1 \cap \mathscr{T}_2 \cap \mathscr{T}_3$*, we have*

$$\|\widetilde{\boldsymbol{\xi}} - \boldsymbol{\xi}^*\|_1 \leq 2\mu_{init} q^*/\varphi^2, \quad \mathbb{P}_N(\boldsymbol{U}^\intercal \widetilde{\boldsymbol{\xi}} - \boldsymbol{U}^\intercal \boldsymbol{\xi}^*)^2 \leq 2\mu_{init}^2 q^*/\varphi^2 \ \text{ and } \ \operatorname{supp}(\widetilde{\boldsymbol{\xi}}) = \mathcal{Q}^*.$$

Lemma S7. *Under assumptions A1 – A8, on the event* $\mathscr{T}_1 \cap \mathscr{T}_2 \cap \mathscr{T}_3$*, we have* $\|\widehat{\boldsymbol{\xi}} - \boldsymbol{\xi}^*\|_1 \leq 2\mu_{init} q^*/\varphi^2$ *and* $\operatorname{supp}(\widehat{\boldsymbol{\xi}}) = \mathcal{Q}^*$.

Lemma S8. *Under assumptions A1 – A8, on the event* $\mathscr{T}_1 \cap \mathscr{T}_2 \cap \mathscr{T}_3$*, we have* $\|\widehat{\boldsymbol{\alpha}} - \boldsymbol{\alpha}^*\|_1 \leq \Delta_{\boldsymbol{\alpha}}$ *and* $\operatorname{supp}(\widehat{\boldsymbol{\alpha}}) = \operatorname{supp}(\boldsymbol{\alpha}^*) = \mathcal{A}^*$*, where* $\Delta_{\boldsymbol{\alpha}} = 2\mu_{init} q^*/\varphi^2$.

Proof. The conclusions follow directly from Lemma S7 and the definition of $\boldsymbol{\xi} = (\boldsymbol{\alpha}^\intercal, \tau)^\intercal$. □

S2.3.3 Proof of the Auxiliary Lemmas

Lemma S9. *Under the assumption A4, if* $\mathbb{E}(\boldsymbol{U}^\intercal \boldsymbol{\xi})^2 \leq a^2$ *for some* $a > 0$ *and* $\|\boldsymbol{\xi}_{\mathcal{Q}^{*c}}\|_1 \leq 3\|\boldsymbol{\xi}_{\mathcal{Q}^*}\|_1$*, then* $\|\boldsymbol{\xi}\|_1 \leq 4a(q^*)^{1/2}/\varphi$*, where* $q^* = |\mathcal{Q}^*|$.

Proof. By A4, we have $\|\boldsymbol{\xi}_{\mathcal{Q}^*}\|_2 \leq \{\mathbb{E}(\boldsymbol{U}^\intercal \boldsymbol{\xi})^2/\varphi^2\}^{1/2} \leq a/\varphi$. Hence

$$\|\boldsymbol{\xi}\|_1 = \|\boldsymbol{\xi}_{\mathcal{Q}^*}\|_1 + \|\boldsymbol{\xi}_{\mathcal{Q}^{*c}}\|_1 \leq 4\|\boldsymbol{\xi}_{\mathcal{Q}^*}\|_1 \leq 4(q^*)^{1/2}\|\boldsymbol{\xi}_{\mathcal{Q}^*}\|_2 \leq 4a(q^*)^{1/2}/\varphi.$$

□

S2.4 Details About Step 2

Recall that the proposed estimator for $\boldsymbol{\theta}_0$ is

$$\widehat{\boldsymbol{\theta}} = \arg\min_{\boldsymbol{\theta}} \mathbb{P}_n\, \ell(Y, \boldsymbol{Z}_{\widehat{\boldsymbol{\alpha}}}^{\intercal}\boldsymbol{\theta}) + \lambda_1 \|\boldsymbol{\theta}_{\widehat{\mathcal{A}}}\|_1 + \lambda_2 \|\boldsymbol{\theta}_{\mathcal{P}\setminus\widehat{\mathcal{A}}}\|_1.$$

On the event $\mathscr{T}_1 \cap \mathscr{T}_2 \cap \mathscr{T}_3$, we have $\widehat{\mathcal{A}} = \mathcal{A}^*$ which is the support of $\boldsymbol{\alpha}^*$. Hence, on the event $\mathscr{T}_1 \cap \mathscr{T}_2 \cap \mathscr{T}_3$,

$$\widehat{\boldsymbol{\theta}} = \arg\min_{\boldsymbol{\theta}} \mathbb{P}_n\, \ell(Y, \boldsymbol{Z}_{\boldsymbol{\alpha}^*}^{\intercal}\widehat{\Psi}_+(\boldsymbol{\theta})) + \lambda_1 \|\boldsymbol{\theta}_{\mathcal{A}^*}\|_1 + \lambda_2 \|\boldsymbol{\theta}_{\mathcal{P}\setminus\mathcal{A}^*}\|_1.$$

The excess risk is

$$\mathbb{E}\, \ell(Y, \boldsymbol{Z}_{\widehat{\boldsymbol{\alpha}}}^{\intercal}\widehat{\boldsymbol{\theta}}) - \mathbb{E}\, \ell(Y, \boldsymbol{Z}_{\boldsymbol{\alpha}^*}^{\intercal}\boldsymbol{\theta}_0) = \mathbb{E}\, \ell(Y, \boldsymbol{Z}_{\boldsymbol{\alpha}^*}^{\intercal}\widehat{\Psi}_+(\widehat{\boldsymbol{\theta}})) - \mathbb{E}\, \ell(Y, \boldsymbol{Z}_{\boldsymbol{\alpha}^*}^{\intercal}\boldsymbol{\theta}_0) = L_0(\widehat{\Psi}_+(\widehat{\boldsymbol{\theta}})) - L_0(\boldsymbol{\theta}_0),$$

where $L_0(\boldsymbol{\theta}) = \mathbb{E}\, \ell(Y, \boldsymbol{Z}_{\boldsymbol{\alpha}^*}^{\intercal}\boldsymbol{\theta})$. The behavior of L_0 near $\boldsymbol{\theta}_0$ is examined in Section 2.3.1. The linear prediction error is

$$\mathbb{E}(\boldsymbol{Z}_{\widehat{\boldsymbol{\alpha}}}^{\intercal}\widehat{\boldsymbol{\theta}} - \boldsymbol{Z}_{\boldsymbol{\alpha}^*}^{\intercal}\boldsymbol{\theta}_0)^2 = \mathbb{E}(\boldsymbol{Z}_{\boldsymbol{\alpha}^*}^{\intercal}\widehat{\Psi}_+(\widehat{\boldsymbol{\theta}}) - \boldsymbol{Z}_{\boldsymbol{\alpha}^*}^{\intercal}\boldsymbol{\theta}_0)^2,$$

and the probability prediction error is

$$\mathbb{E}\{\sigma(\boldsymbol{Z}_{\widehat{\boldsymbol{\alpha}}}^{\intercal}\widehat{\boldsymbol{\theta}}) - \sigma(\boldsymbol{Z}_{\boldsymbol{\alpha}^*}^{\intercal}\boldsymbol{\theta}_0)\}^2 = \mathbb{E}\{\sigma(\boldsymbol{Z}_{\boldsymbol{\alpha}^*}^{\intercal}\widehat{\Psi}_+(\widehat{\boldsymbol{\theta}})) - \sigma(\boldsymbol{Z}_{\boldsymbol{\alpha}^*}^{\intercal}\boldsymbol{\theta}_0)\}^2.$$

Both prediction errors are related to excess risk, as seen from Section 2.3.2.

S2.4.1 Quadratic Approximation of the Log-likelihood

Lemma S10. *For any $\boldsymbol{\theta}$ such that $\|\boldsymbol{H}^{1/2}(\boldsymbol{\theta} - \boldsymbol{\theta}_0)\|_2 \le \eta$, we have*

$$\frac{1}{4}(\boldsymbol{\theta} - \boldsymbol{\theta}_0)^{\intercal}\boldsymbol{H}(\boldsymbol{\theta} - \boldsymbol{\theta}_0) \le L_0(\boldsymbol{\theta}) - L_0(\boldsymbol{\theta}_0) \le \frac{3}{4}(\boldsymbol{\theta} - \boldsymbol{\theta}_0)^{\intercal}\boldsymbol{H}(\boldsymbol{\theta} - \boldsymbol{\theta}_0).$$

Note that although $\boldsymbol{\theta}_0$ is not unique due to multicollinearity among $\boldsymbol{Z}_{\boldsymbol{\alpha}^*}$, all the quantities in Lemma S10 relies on $\boldsymbol{\theta}_0$ through $\boldsymbol{Z}_{\boldsymbol{\alpha}^*}^{\intercal}\boldsymbol{\theta}_0$, which is unique.

S2.4.2 Connections Among Excess Risk, Linear Prediction Error and Probability Prediction Error

Lemma S11. *We have*

$$\begin{aligned}
\mathbb{E}\, \ell(Y, \boldsymbol{Z}_{\widehat{\boldsymbol{\alpha}}}^{\intercal}\widehat{\boldsymbol{\theta}}) - \mathbb{E}\, \ell(Y, \boldsymbol{Z}_{\boldsymbol{\alpha}^*}^{\intercal}\boldsymbol{\theta}_0) &\le \|\boldsymbol{H}^{1/2}(\widehat{\Psi}_+(\widehat{\boldsymbol{\theta}}) - \boldsymbol{\theta}_0)\|_2^2, \\
\mathbb{E}(\boldsymbol{Z}_{\widehat{\boldsymbol{\alpha}}}^{\intercal}\widehat{\boldsymbol{\theta}} - \boldsymbol{Z}_{\boldsymbol{\alpha}^*}^{\intercal}\boldsymbol{\theta}_0)^2 &\le \|\boldsymbol{H}^{1/2}(\widehat{\Psi}_+(\widehat{\boldsymbol{\theta}}) - \boldsymbol{\theta}_0)\|_2^2/\varpi, \\
\mathbb{E}\{\sigma(\boldsymbol{Z}_{\widehat{\boldsymbol{\alpha}}}^{\intercal}\widehat{\boldsymbol{\theta}}) - \sigma(\boldsymbol{Z}_{\boldsymbol{\alpha}^*}^{\intercal}\boldsymbol{\theta}_0)\}^2 &\le \|\boldsymbol{H}^{1/2}(\widehat{\Psi}_+(\widehat{\boldsymbol{\theta}}) - \boldsymbol{\theta}_0)\|_2^2/\varpi.
\end{aligned}$$

S2.5 Details About Step 3

Throughout this subsection, define $D^2 = 64\mathscr{E}(\boldsymbol{\theta}^*, \mathcal{S}_+^*, \mathcal{S}_-^*)$ and $\Lambda(n, k, \epsilon) = \{\log(2k/\epsilon)/n\}^{1/2}$. We shall state a few lemmas. The proof of those lemmas are deferred to Section 2.4.2.

Lemma S12. *Let $k \in [0, 1]$ be a constant. Define a random vector $\widetilde{\boldsymbol{\theta}} = k\widehat{\Psi}_+(\widehat{\boldsymbol{\theta}}) + (1-k)\boldsymbol{\theta}^*$. Then $\widetilde{\boldsymbol{\theta}}$ satisfies*

$$L_n(\widetilde{\boldsymbol{\theta}}) + \Omega(\widetilde{\boldsymbol{\theta}}_{\mathcal{P}}) \le L_n(\boldsymbol{\theta}^*) + \Omega(\boldsymbol{\theta}^*_{\mathcal{P}}) + \frac{1}{4}\Omega(\widetilde{\boldsymbol{\theta}}_{\mathcal{S}_+^*} - \boldsymbol{\theta}^*_{\mathcal{S}_+^*}) + 2\lambda_1\Delta_{\boldsymbol{\alpha}}|\rho^*|.$$

Lemma S13. *Let $k \in [0, 1]$ be a constant. For any random vector $\widetilde{\boldsymbol{\theta}}$, we have*

$$\Omega(\boldsymbol{\theta}^*_{\mathcal{P}}) - \Omega(\widetilde{\boldsymbol{\theta}}_{\mathcal{P}}) + k\Omega(\widetilde{\boldsymbol{\theta}} - \boldsymbol{\theta}^*) \le (1+k)\Omega(\widetilde{\boldsymbol{\theta}}_{\mathcal{S}_+^*} - \boldsymbol{\theta}^*_{\mathcal{S}_+^*}) - (1-k)\Omega(\widetilde{\boldsymbol{\theta}}_{\mathcal{S}_+^{*c}} - \boldsymbol{\theta}^*_{\mathcal{S}_+^{*c}}) + 2\Omega(\boldsymbol{\theta}^*_{\mathcal{S}_-^*}).$$

Lemma S14. *Denote by $\mathscr{R} = \{\boldsymbol{v} \in \mathbb{R}^{p+3} : \Omega(\boldsymbol{v}_{\mathcal{P}\setminus\mathcal{S}_+^*}) \le 3\Omega(\boldsymbol{v}_{\mathcal{S}_+^*})\}$. Suppose $\boldsymbol{v} \in \mathscr{R}$. Under assumptions A1 – A8 we have*

$$\|\boldsymbol{v}_{\mathcal{S}_+^*}\|_1 \le (\varpi\phi)^{-1/2}|\mathcal{S}_+^*|^{1/2}\|\boldsymbol{H}^{1/2}\boldsymbol{v}\|_2,$$
$$\Omega(\boldsymbol{v}_{\mathcal{S}_+^*}) \le \kappa^*(\varpi\phi)^{-1/2}|\mathcal{S}_+^*|^{1/2}\|\boldsymbol{H}^{1/2}\boldsymbol{v}\|_2.$$

Lemma S15. *Let $\widetilde{\boldsymbol{\theta}}$ be a random vector that satisfies*

$$L_n(\widetilde{\boldsymbol{\theta}}) + \Omega(\widetilde{\boldsymbol{\theta}}_{\mathcal{P}}) \le L_n(\boldsymbol{\theta}^*) + \Omega(\boldsymbol{\theta}^*_{\mathcal{P}}) + \frac{1}{4}\Omega(\widetilde{\boldsymbol{\theta}}_{\mathcal{S}_+^*} - \boldsymbol{\theta}^*_{\mathcal{S}_+^*}) + 2\lambda_1\Delta_{\boldsymbol{\alpha}}|\rho^*|.$$

For any $\epsilon > 0$, if $\lambda_0 \ge 8B\Lambda(n, 3, \epsilon)$, $\lambda_1 \ge 8B\Lambda(n, q^*, \epsilon)$ and $\lambda_2 \ge 8B\Lambda(n, p, \epsilon)$, then with probability at least $1 - 3\epsilon$, $\widetilde{\boldsymbol{\theta}}$ satisfies

$$\frac{3}{4}\Omega(\widetilde{\boldsymbol{\theta}}_{\mathcal{S}_+^{*c}} - \boldsymbol{\theta}^*_{\mathcal{S}_+^{*c}}) \le \frac{3}{2}\Omega(\widetilde{\boldsymbol{\theta}}_{\mathcal{S}_+^*} - \boldsymbol{\theta}^*_{\mathcal{S}_+^*}) + 2\Omega(\boldsymbol{\theta}^*_{\mathcal{S}_-^*}) + 2\lambda_1\Delta_{\boldsymbol{\alpha}}|\rho^*|.$$

Lemma S16. *Fix $M_0, M_1, M_2 > 0$. Let*

$$\mathscr{M} = \{\boldsymbol{\theta} = (\boldsymbol{\delta}^{\mathsf{T}}, \rho, \gamma, \zeta) : |\rho - \rho^*| + |\gamma - \gamma^*| + |\zeta - \zeta^*| \le M_0,$$
$$\|(\boldsymbol{\delta} - \boldsymbol{\delta}^*)_{\mathcal{A}^*}\|_1 \le M_1, \|(\boldsymbol{\delta} - \boldsymbol{\delta}^*)_{\mathcal{P}\setminus\mathcal{A}^*}\|_1 \le M_2\}.$$

Define

$$\mathbb{G}_n = \sup_{\boldsymbol{\theta}\in\mathscr{M}}|L_n(\boldsymbol{\theta}) - L_n(\boldsymbol{\theta}^*) - L_0(\boldsymbol{\theta}) + L_0(\boldsymbol{\theta}^*)|$$

For any $\epsilon > 0$, if $\lambda_0 \ge 36B\Lambda(n, 3, \epsilon)$, $\lambda_1 \ge 36B\Lambda(n, q^*, \epsilon)$ and $\lambda_2 \ge 36B\Lambda(n, p, \epsilon)$, then with probability at least $1 - 2\epsilon$ we have

$$\mathbb{G}_n \le \frac{1}{4}(\lambda_0 M_0 + \lambda_1 M_1 + \lambda_2 M_2).$$

S2.5.1 Proof of the Main Results

Lemma S17. *Under assumptions A1 – A8, with probability at least* $1-10\epsilon$*, we have*

$$\|\boldsymbol{H}^{1/2}(\boldsymbol{\theta}^*-\boldsymbol{\theta}_0)\|_2 \le \frac{1}{4}D,$$
$$\|\boldsymbol{H}^{1/2}(\widehat{\Psi}_{+}(\widehat{\boldsymbol{\theta}})-\boldsymbol{\theta}^*)\|_2 \le \frac{3}{4}D.$$

Proof. The definitions of $\boldsymbol{\theta}^*$ and D^2 imply that

$$\|\boldsymbol{G}^{1/2}(\boldsymbol{\theta}^*-\boldsymbol{\theta}_0)\|_2 \le \eta,$$

and

$$\begin{aligned}
&D^2/64 \ge \mathbb{E}\,\ell(Y,\boldsymbol{Z}_{\boldsymbol{\alpha}^*}^{\mathsf{T}}\boldsymbol{\theta}^*) - \mathbb{E}\,\ell(Y,\boldsymbol{Z}_{\boldsymbol{\alpha}^*}^{\mathsf{T}}\boldsymbol{\theta}_0) = L(\boldsymbol{\theta}^*) - L(\boldsymbol{\theta}_0),\\
&D^2/64 \ge 256(\kappa^*)^2|\mathcal{S}_{+}^*|/(\varpi\phi),\\
&D^2/64 \ge 8\lambda_1\|\boldsymbol{\theta}^*_{\mathcal{S}_{-}^*\cap\mathcal{A}^*}\|_1 + 8\lambda_2\|\boldsymbol{\theta}^*_{\mathcal{S}_{-}^*\cap(\mathcal{P}\setminus\mathcal{A}^*)}\|_1 + 8\lambda_1\Delta_{\boldsymbol{\alpha}}\Pi(\boldsymbol{\theta}^*) = 8\Omega(\boldsymbol{\theta}^*_{\mathcal{S}_{-}^*}) + 8\lambda_1\Delta_{\boldsymbol{\alpha}}|\rho^*|,
\end{aligned}$$

where $\boldsymbol{\theta}^* = ((\boldsymbol{\delta}^*)^{\mathsf{T}},\rho^*,\gamma^*,\zeta^*)^{\mathsf{T}}$.

For the first inequality, by Lemma S10 and $\|\boldsymbol{G}^{1/2}(\boldsymbol{\theta}^*-\boldsymbol{\theta}_0)\|_2 \le \eta$, we have

$$\|\boldsymbol{H}^{1/2}(\boldsymbol{\theta}^*-\boldsymbol{\theta}_0)\|_2^2 \le 4\{L_0(\boldsymbol{\theta}^*) - L_0(\boldsymbol{\theta}_0)\} \le D^2/16.$$

Thus

$$\|\boldsymbol{H}^{1/2}(\boldsymbol{\theta}^*-\boldsymbol{\theta}_0)\|_2 \le \frac{1}{4}D.$$

For the second inequality, we will prove by contradiction. Suppose the estimator $\widehat{\boldsymbol{\theta}}$ satisfies

$$\|\boldsymbol{H}^{1/2}(\widehat{\Psi}_{+}(\widehat{\boldsymbol{\theta}})-\boldsymbol{\theta}^*)\|_2 > \frac{3}{4}D.$$

Let $\widetilde{\boldsymbol{\theta}} = t\widehat{\Psi}_{+}(\widehat{\boldsymbol{\theta}}) + (1-t)\boldsymbol{\theta}^*$, where

$$t = \frac{3D/4}{\|\boldsymbol{H}^{1/2}(\widehat{\Psi}_{+}(\widehat{\boldsymbol{\theta}})-\boldsymbol{\theta}^*)\|_2} \in [0,1].$$

Then we have

$$\|\boldsymbol{H}^{1/2}(\widetilde{\boldsymbol{\theta}}-\boldsymbol{\theta}^*)\|_2 = t\|\boldsymbol{H}^{1/2}(\widehat{\Psi}_{+}(\widehat{\boldsymbol{\theta}})-\boldsymbol{\theta}^*)\|_2 = \frac{3}{4}D.$$

Besides, by Lemma S12, we have

$$L_n(\widetilde{\boldsymbol{\theta}}) + \Omega(\widetilde{\boldsymbol{\theta}}_{\mathcal{P}}) \le L_n(\boldsymbol{\theta}^*) + \Omega(\boldsymbol{\theta}^*_{\mathcal{P}}) + \frac{1}{4}\Omega(\widetilde{\boldsymbol{\theta}}_{\mathcal{S}_{+}^*} - \boldsymbol{\theta}^*_{\mathcal{S}_{+}^*}) + 2\lambda_1\Delta_{\boldsymbol{\alpha}}|\rho^*|. \tag{S3}$$

Hence we obtain

$$L_0(\widetilde{\boldsymbol{\theta}}) - L_0(\boldsymbol{\theta}_0) \le I_1 + I_2 + I_3, \tag{S4}$$

where

$$I_1 = L_0(\boldsymbol{\theta}^*) - L_0(\boldsymbol{\theta}_0),$$
$$I_2 = \Omega(\boldsymbol{\theta}^*_{\mathcal{P}}) - \Omega(\widetilde{\boldsymbol{\theta}}_{\mathcal{P}}) + \frac{1}{4}\Omega(\widetilde{\boldsymbol{\theta}}_{\mathcal{S}^*_+} - \boldsymbol{\theta}^*_{\mathcal{S}^*_+}) + 2\lambda_1\Delta_{\boldsymbol{\alpha}}|\rho^*|,$$
$$I_3 = L_0(\widetilde{\boldsymbol{\theta}}) - L_0(\boldsymbol{\theta}^*) - L_n(\widetilde{\boldsymbol{\theta}}) + L_n(\boldsymbol{\theta}^*).$$

By the first inequality and the triangle inequality, we know

$$\|\boldsymbol{H}^{1/2}(\widetilde{\boldsymbol{\theta}} - \boldsymbol{\theta}_0)\|_2 \geq \|\boldsymbol{H}^{1/2}(\widetilde{\boldsymbol{\theta}} - \boldsymbol{\theta}^*)\|_2 - \|\boldsymbol{H}^{1/2}(\boldsymbol{\theta}^* - \boldsymbol{\theta}_0)\|_2 \geq \frac{3}{4}D - \frac{1}{4}D = \frac{1}{2}D.$$

By Lemma S10, we have

$$L_0(\widetilde{\boldsymbol{\theta}}) - L_0(\boldsymbol{\theta}_0) \geq \frac{1}{4}\|\boldsymbol{H}^{1/2}(\widetilde{\boldsymbol{\theta}} - \boldsymbol{\theta}_0)\|_2^2 \geq \frac{1}{16}D^2.$$

Hence equation (S4) implies

$$D^2/16 \leq I_1 + I_2 + I_3. \tag{S5}$$

By the definition of D^2, we have

$$I_1 \leq D^2/64. \tag{S6}$$

By Lemma S13 with $k = 0$, we have

$$I_2 \leq \frac{5}{4}\Omega(\widetilde{\boldsymbol{\theta}}_{\mathcal{S}^*_+} - \boldsymbol{\theta}^*_{\mathcal{S}^*_+}) + 2\Omega(\boldsymbol{\theta}^*_{\mathcal{S}^*_-}) + 2\lambda_1\Delta_{\boldsymbol{\alpha}}|\rho^*| \tag{S7}$$

Moreover, from equation (S3) and Lemma S15, with probability at least $1 - 3\epsilon$,

$$\frac{3}{4}\Omega(\widetilde{\boldsymbol{\theta}}_{\mathcal{S}^{*c}_+} - \boldsymbol{\theta}^*_{\mathcal{S}^{*c}_+}) \leq \frac{3}{2}\Omega(\widetilde{\boldsymbol{\theta}}_{\mathcal{S}^*_+} - \boldsymbol{\theta}^*_{\mathcal{S}^*_+}) + \big\{2\Omega(\boldsymbol{\theta}^*_{\mathcal{S}^*_-}) + 2\lambda_1\Delta_{\boldsymbol{\alpha}}|\rho^*|\big\}. \tag{S8}$$

We shall proceed the proof depending on whether

$$\frac{3}{4}\Omega(\widetilde{\boldsymbol{\theta}}_{\mathcal{S}^*_+} - \boldsymbol{\theta}^*_{\mathcal{S}^*_+}) \leq 2\Omega(\boldsymbol{\theta}^*_{\mathcal{S}^*_-}) + 2\lambda_1\Delta_{\boldsymbol{\alpha}}|\rho^*|$$

or

$$\frac{3}{4}\Omega(\widetilde{\boldsymbol{\theta}}_{\mathcal{S}^*_+} - \boldsymbol{\theta}^*_{\mathcal{S}^*_+}) > 2\Omega(\boldsymbol{\theta}^*_{\mathcal{S}^*_-}) + 2\lambda_1\Delta_{\boldsymbol{\alpha}}|\rho^*|.$$

Case 1 Suppose

$$\frac{3}{4}\Omega(\widetilde{\boldsymbol{\theta}}_{\mathcal{S}^*_+} - \boldsymbol{\theta}^*_{\mathcal{S}^*_+}) \leq 2\Omega(\boldsymbol{\theta}^*_{\mathcal{S}^*_-}) + 2\lambda_1\Delta_{\boldsymbol{\alpha}}|\rho^*|.$$

Then, by equation (S8), we have

$$\Omega(\widetilde{\boldsymbol{\theta}}_{\mathcal{S}^*_+} - \boldsymbol{\theta}^*_{\mathcal{S}^*_+}) \leq \frac{8}{3}\{\Omega(\boldsymbol{\theta}^*_{\mathcal{S}^*_-}) + \lambda_1\Delta_{\boldsymbol{\alpha}}|\rho^*|\},$$
$$\Omega(\widetilde{\boldsymbol{\theta}}_{\mathcal{S}^{*c}_+} - \boldsymbol{\theta}^*_{\mathcal{S}^{*c}_+}) \leq 8\{\Omega(\boldsymbol{\theta}^*_{\mathcal{S}^*_-}) + \lambda_1\Delta_{\boldsymbol{\alpha}}|\rho^*|\}.$$

Hence

$$\Omega(\widetilde{\boldsymbol{\theta}} - \boldsymbol{\theta}^*) \leq \Omega(\widetilde{\boldsymbol{\theta}}_{\mathcal{S}_+^*} - \boldsymbol{\theta}^*_{\mathcal{S}_+^*}) + \Omega(\widetilde{\boldsymbol{\theta}}_{\mathcal{S}_+^{*c}} - \boldsymbol{\theta}^*_{\mathcal{S}_+^{*c}}) \leq \frac{32}{3}\{\Omega(\boldsymbol{\theta}^*_{\mathcal{S}_-^*}) + \lambda_1 \Delta_{\boldsymbol{\alpha}} |\rho^*|\}.$$

By the definitions of Ω and D^2, we know $D^2/64 \geq 8\Omega(\boldsymbol{\theta}^*_{\mathcal{S}_-^*}) + 8\lambda_1 \Delta_{\boldsymbol{\alpha}}|\rho^*|$. Thus, $\Omega(\widetilde{\boldsymbol{\theta}} - \boldsymbol{\theta}^*) \leq D^2/48$. Thus by Lemma S16 with $M_k = D^2/(48\lambda_k)$, $k = 0, 1, 2$, with probability at least $1 - 2\epsilon$,

$$I_3 \leq \sup_{\boldsymbol{\theta}:\Omega(\boldsymbol{\theta}-\boldsymbol{\theta}^*)\leq D^2/48} |L_n(\boldsymbol{\theta}) - L_n(\boldsymbol{\theta}^*) - L_0(\boldsymbol{\theta}) + L_0(\boldsymbol{\theta}^*)| \leq D^2/64. \tag{S9}$$

Equation (S6) says $I_1 \leq D^2/64$. In addition, by equation (S7), we have

$$I_2 \leq \frac{16}{3}\{\Omega(\boldsymbol{\theta}^*_{\mathcal{S}_-^*}) + \lambda_1 \Delta_{\boldsymbol{\alpha}}|\rho^*|\} \leq D^2/96.$$

Hence we conclude that $I_1 + I_2 + I_3 \leq 3D^2/64$, which contradicts with equation (S5).

Case 2 Suppose

$$\frac{3}{4}\Omega(\widetilde{\boldsymbol{\theta}}_{\mathcal{S}_+^*} - \boldsymbol{\theta}^*_{\mathcal{S}_+^*}) > 2\Omega(\boldsymbol{\theta}^*_{\mathcal{S}_-^*}) + 2\lambda_1 \Delta_{\boldsymbol{\alpha}}|\rho^*|.$$

Then by equation (S8) we have

$$\Omega(\widetilde{\boldsymbol{\theta}}_{\mathcal{S}_+^{*c}} - \boldsymbol{\theta}^*_{\mathcal{S}_+^{*c}}) \leq 3\Omega(\widetilde{\boldsymbol{\theta}}_{\mathcal{S}_+^*} - \boldsymbol{\theta}^*_{\mathcal{S}_+^*}),$$

and thus $\widetilde{\boldsymbol{\theta}} - \boldsymbol{\theta}^* \in \mathscr{R}$. In addition,

$$\Omega(\widetilde{\boldsymbol{\theta}} - \boldsymbol{\theta}^*) = \Omega(\widetilde{\boldsymbol{\theta}}_{\mathcal{S}_+^*} - \boldsymbol{\theta}^*_{\mathcal{S}_+^*}) + \Omega(\widetilde{\boldsymbol{\theta}}_{\mathcal{S}_+^{*c}} - \boldsymbol{\theta}^*_{\mathcal{S}_+^{*c}}) \leq 4\Omega(\widetilde{\boldsymbol{\theta}}_{\mathcal{S}_+^*} - \boldsymbol{\theta}^*_{\mathcal{S}_+^*}).$$

By Lemma S14 and $\|\boldsymbol{H}^{1/2}(\widetilde{\boldsymbol{\theta}} - \boldsymbol{\theta}^*)\|_2 = 3D/4$, we have

$$\Omega(\widetilde{\boldsymbol{\theta}}_{\mathcal{S}_+^*} - \boldsymbol{\theta}^*_{\mathcal{S}_+^*}) \leq \kappa^*(\varpi\phi)^{-1/2}|\mathcal{S}_+^*|^{1/2}\|\boldsymbol{H}^{1/2}(\widetilde{\boldsymbol{\theta}} - \boldsymbol{\theta}^*)\|_2 = \frac{3}{4}\kappa^*(\varpi\phi)^{-1/2}|\mathcal{S}_+^*|^{1/2}D.$$

So $\Omega(\widetilde{\boldsymbol{\theta}} - \boldsymbol{\theta}^*) \leq 3\kappa^*(\varpi\phi)^{-1/2}|\mathcal{S}_+^*|^{1/2}D$. By Lemma S16 with $M_k = 3\kappa^*(\varpi\phi)^{-1/2}|\mathcal{S}_+^*|^{1/2}D/\lambda_k$, $k = 0, 1, 2$, with probability at least $1 - 2\epsilon$,

$$\begin{aligned} I_3 \leq & \sup_{\boldsymbol{\theta}:\Omega(\boldsymbol{\theta}-\boldsymbol{\theta}^*)\leq 3\kappa^*(\varpi\phi)^{-1/2}|\mathcal{S}_+^*|^{1/2}D} |L_n(\boldsymbol{\theta}) - L_n(\boldsymbol{\theta}^*) - L_0(\boldsymbol{\theta}) + L_0(\boldsymbol{\theta}^*)| \\ & \leq \frac{9}{4}\kappa^*(\varpi\phi)^{-1/2}|\mathcal{S}_+^*|^{1/2}D. \end{aligned} \tag{S10}$$

Equation (S6) says $I_1 \leq D^2/64$. In addition, by equation (S7), we have

$$I_2 \leq 2\Omega(\widetilde{\boldsymbol{\theta}}_{\mathcal{S}_+^*} - \boldsymbol{\theta}^*_{\mathcal{S}_+^*}) \leq \frac{3}{2}\kappa^*(\varpi\phi)^{-1/2}|\mathcal{S}_+^*|^{1/2}D.$$

Hence we conclude that

$$I_1 + I_2 + I_3 \leq D^2/64 + 4\kappa^*(\varpi\phi)^{-1/2}|\mathcal{S}_+^*|^{1/2}D.$$

Since $4ab \le 256a^2 + b^2/64$ and $D^2/64 \ge 256(\kappa^*)^2(\varpi\phi)^{-1}|\mathcal{S}_+^*|$, we have

$$I_1 + I_2 + I_3 \le D^2/64 + 256(\kappa^*)^2(\varpi\phi)^{-1}|\mathcal{S}_+^*| + D^2/64 \le 3D^2/64,$$

which contradicts with equation (S5). Therefore, no matter under case 1 or case 2, equation (S5) fails to hold. Hence we conclude that it is impossible that $\|\boldsymbol{H}^{1/2}(\widehat{\Psi}_+(\widehat{\boldsymbol{\theta}}) - \boldsymbol{\theta}^*)\|_2 > 3D/4$. Consequently, the second inequality holds.

Finally, we observe that all these bounds are valid on the intersection of the following events:

$\mathscr{T}_1$ occurs, which happens with probability at least $1 - \epsilon$,
$\mathscr{T}_2$ occurs, which happens with probability at least $1 - \epsilon$,
$\mathscr{T}_3$ occurs, which happens with probability at least $1 - \epsilon$,
equation (S8) holds, which happens with probability at least $1 - 3\epsilon$,
equation (S9) holds, which happens with probability at least $1 - 2\epsilon$,
equation (S10) holds, which happens with probability at least $1 - 2\epsilon$.

Hence, these bounds are valid with probability at least $1 - 10\epsilon$. □

Theorem 1 in the main text is a direct consequence of Lemma S17, as shown below.

Proof of Theorem 1 in the main text. By Lemma S17 we have, with probability at least $1 - 10\epsilon$,

$$\|\boldsymbol{H}^{1/2}(\boldsymbol{\theta}^* - \boldsymbol{\theta}_0)\|_2 \le \frac{1}{4}D, \quad \|\boldsymbol{H}^{1/2}(\widehat{\Psi}_+(\widehat{\boldsymbol{\theta}}) - \boldsymbol{\theta}^*)\|_2 \le \frac{3}{4}D.$$

Hence

$$\|\boldsymbol{H}^{1/2}(\widehat{\Psi}_+(\widehat{\boldsymbol{\theta}}) - \boldsymbol{\theta}_0)\|_2 \le \|\boldsymbol{H}^{1/2}(\widehat{\Psi}_+(\widehat{\boldsymbol{\theta}}) - \boldsymbol{\theta}^*)\|_2 + \|\boldsymbol{H}^{1/2}(\widehat{\Psi}_+(\boldsymbol{\theta}^*) - \boldsymbol{\theta}_0)\|_2 \le D$$

with probability at least $1 - 10\epsilon$. Then, by Lemma S11, we have, with probability at least $1 - 10\epsilon$, the following inequalities hold simultaneously:

$$\begin{aligned}
\mathbb{E}\,\ell(Y, \boldsymbol{Z}_{\widehat{\boldsymbol{\alpha}}}^{\mathsf{T}}\widehat{\boldsymbol{\theta}}) - \mathbb{E}\,\ell(Y, \boldsymbol{Z}_{\boldsymbol{\alpha}^*}^{\mathsf{T}}\boldsymbol{\theta}_0) &\le D^2,\\
\mathbb{E}(\boldsymbol{Z}_{\widehat{\boldsymbol{\alpha}}}^{\mathsf{T}}\widehat{\boldsymbol{\theta}} - \boldsymbol{Z}_{\boldsymbol{\alpha}^*}^{\mathsf{T}}\boldsymbol{\theta}_0)^2 &\le D^2/\varpi,\\
\mathbb{E}\{\sigma(\boldsymbol{Z}_{\widehat{\boldsymbol{\alpha}}}^{\mathsf{T}}\widehat{\boldsymbol{\theta}}) - \sigma(\boldsymbol{Z}_{\boldsymbol{\alpha}^*}^{\mathsf{T}}\boldsymbol{\theta}_0)\}^2 &\le D^2/\varpi,
\end{aligned}$$

where $D^2 = 64\mathscr{E}(\boldsymbol{\theta}^*, \mathcal{S}_+^*, \mathcal{S}_-^*)$. □

S2.6 Proof of the Auxiliary Lemmas

Proof of Lemma S1. Since $\boldsymbol{\xi}^*$ is the minimizer of $\mathbb{E}(S - \boldsymbol{U}^{\mathsf{T}}\boldsymbol{\xi})^2$, by taking the derivative under expectation, we obtain that

$$\mathbb{E}\{U_j(S - \boldsymbol{U}^{\mathsf{T}}\boldsymbol{\xi}^*)\} = 0$$

for all j. Since $\mathbb{E}\{(\boldsymbol{\xi} - \boldsymbol{\xi}^*)^{\mathsf{T}}\boldsymbol{U}(S - \boldsymbol{U}^{\mathsf{T}}\boldsymbol{\xi}^*)\} = (\boldsymbol{\xi} - \boldsymbol{\xi}^*)^{\mathsf{T}}\,\mathbb{E}\{\boldsymbol{U}(S - \boldsymbol{U}^{\mathsf{T}}\boldsymbol{\xi}^*)\} = 0$, we have

$$\begin{aligned}
\mathbb{E}(S - \boldsymbol{U}^{\mathsf{T}}\boldsymbol{\xi})^2 - \mathbb{E}(S - \boldsymbol{U}^{\mathsf{T}}\boldsymbol{\xi}^*)^2 &= \mathbb{E}\{(S - \boldsymbol{U}^{\mathsf{T}}\boldsymbol{\xi}^*) + (\boldsymbol{U}^{\mathsf{T}}\boldsymbol{\xi}^* - \boldsymbol{U}^{\mathsf{T}}\boldsymbol{\xi})\}^2 - \mathbb{E}(S - \boldsymbol{U}^{\mathsf{T}}\boldsymbol{\xi}^*)^2\\
&= \mathbb{E}(\boldsymbol{U}^{\mathsf{T}}\boldsymbol{\xi} - \boldsymbol{U}^{\mathsf{T}}\boldsymbol{\xi}^*)^2 = \mathscr{E}_1(\boldsymbol{\xi}).
\end{aligned}$$

□

Proof of Lemma S2. Fix j. Since U_j and $S - \boldsymbol{U}^\intercal \boldsymbol{\xi}^*$ are sub-Gaussian(B^2), we have

$$\mathbb{E}(|U_j|^{2m}) \leq 2\Gamma(m+1)(2B^2)^m \text{ and } \mathbb{E}(|S - \boldsymbol{U}^\intercal \boldsymbol{\xi}^*|^m) \leq 2\Gamma(m+1)(2B^2)^m,$$

where $\Gamma(\cdot)$ denotes the Gamma function. Thus for any $m \geq 2$,

$$\mathbb{E}|U_j(S - \boldsymbol{U}^\intercal \boldsymbol{\xi}^*)|^m \leq (\mathbb{E}|U_j|^{2m})^{1/2}(\mathbb{E}|S - \boldsymbol{U}^\intercal \boldsymbol{\xi}^*|^{2m})^{1/2} \leq \frac{m!}{2}(2B^2)^{m-2}(4B^2)^2.$$

In addition, we know that $\mathbb{E}\{U_j(S - \boldsymbol{U}^\intercal \boldsymbol{\xi}^*)\} = 0$. Hence by Bernstein's inequality (van de Geer, 2016, Theorem 9.2), we have

$$\Pr\left\{\left|\mathbb{P}_N U_j(S - \boldsymbol{U}^\intercal \boldsymbol{\xi}^*)\right| \geq 4B^2\left(\frac{2t}{N}\right)^{1/2} + 2B^2\frac{t}{N}\right\} \leq e^{-t}.$$

We then apply the union bound and let $t = \log(p/\epsilon)$. Assumption A8 implies $\log(p/\epsilon) \leq N$, which further implies $\log(p/\epsilon)/N \leq \{\log(p/\epsilon)/N\}^{1/2}$. Thus,

$$\Pr\left[\left|\mathbb{P}_N U_j(S - \boldsymbol{U}^\intercal \boldsymbol{\xi}^*)\right| \geq 8B^2\left\{\frac{\log(p/\epsilon)}{N}\right\}^{1/2} \text{ for some } j\right] \leq \epsilon.$$

□

Proof of Lemma S3. For any $\boldsymbol{\xi}$ such that $\mathbb{E}(\boldsymbol{U}^\intercal \boldsymbol{\xi})^2 = 1$ and $\|\boldsymbol{\xi}_{\mathcal{Q}^{*c}}\|_1 \leq 3\|\boldsymbol{\xi}_{\mathcal{Q}^*}\|_1$, by Lemma S9, $\|\boldsymbol{\xi}\|_1 \leq 4(q^*)^{1/2}/\varphi$. Similar to the proof of Lemma S2, we have that for all $j, k \in \{1, \ldots, p+1\}$,

$$\Pr\left\{\left|\mathbb{P}_N U_j U_k - \mathbb{E}(U_j U_k)\right| \geq 4B^2\left(\frac{2t}{N}\right)^{1/2} + 2B^2\frac{t}{N}\right\} \leq e^{-t}.$$

We then apply the union bound and let $t = \log(p^2/\epsilon) \leq 2\log(p/\epsilon)$. Assumption A8 implies $2\log(p/\epsilon) \leq N$, which further implies $2\log(p/\epsilon)/N \leq \{2\log(p/\epsilon)/N\}^{1/2}$. Thus,

$$\Pr\left[\left|\mathbb{P}_N U_j U_k - \mathbb{E}(U_j U_k)\right| \geq 12B^2\left\{\frac{\log(p/\epsilon)}{N}\right\}^{1/2} \text{ for some } j, k \in \{1, \ldots, p+1\}\right] \leq \epsilon.$$

For any $\boldsymbol{\xi}$, we observe that

$$\left|\mathbb{P}_N(\boldsymbol{U}^\intercal \boldsymbol{\xi})^2 - \mathbb{E}(\boldsymbol{U}^\intercal \boldsymbol{\xi})^2\right| = \left|\boldsymbol{\xi}^\intercal\left(\mathbb{P}_N \boldsymbol{U}\boldsymbol{U}^\intercal - \mathbb{E}\,\boldsymbol{U}\boldsymbol{U}^\intercal\right)\boldsymbol{\xi}\right| \leq \max_{j,k}\left|\mathbb{P}_N U_j U_k - \mathbb{E}(U_j U_k)\right|\|\boldsymbol{\xi}\|_1^2.$$

Thus, with probability at least $1 - \epsilon$, we have

$$\sup_{\boldsymbol{\xi}:\|\boldsymbol{\xi}_{\mathcal{Q}^{*c}}\|_1 \leq 3\|\boldsymbol{\xi}_{\mathcal{Q}^*}\|_1, \mathbb{E}(\boldsymbol{U}^\intercal \boldsymbol{\xi})^2 = 1}\left|\mathbb{P}_N(\boldsymbol{U}^\intercal \boldsymbol{\xi})^2 - \mathbb{E}(\boldsymbol{U}^\intercal \boldsymbol{\xi})^2\right| \leq 12B^2\left\{\frac{\log(p/\epsilon)}{N}\right\}^{1/2} \cdot 16(q^*)/\varphi^2$$

By assumption A8, the right-hand side is at most $1/2$. Hence, with probability at least $1-\epsilon$, we have

$$\begin{aligned}
&\inf_{\boldsymbol{\xi}:\|\boldsymbol{\xi}_{\mathcal{Q}^{*c}},\|_1 \leq 3\|\boldsymbol{\xi}_{\mathcal{Q}^*}\|_1, \mathbb{E}(\boldsymbol{U}^\intercal \boldsymbol{\xi})^2 = 1}\left\{\mathbb{P}_N(\boldsymbol{U}^\intercal \boldsymbol{\xi})^2 - \mathbb{E}(\boldsymbol{U}^\intercal \boldsymbol{\xi})^2\right\} \\
&\geq -\sup_{\boldsymbol{\xi}:\|\boldsymbol{\xi}_{\mathcal{Q}^{*c}}\|_1 \leq 3\|\boldsymbol{\xi}_{\mathcal{Q}^*}\|_1, \mathbb{E}(\boldsymbol{U}^\intercal \boldsymbol{\xi})^2 = 1}\left|\mathbb{P}_N(\boldsymbol{U}^\intercal \boldsymbol{\xi})^2 - \mathbb{E}(\boldsymbol{U}^\intercal \boldsymbol{\xi})^2\right| \geq -\frac{1}{2},
\end{aligned}$$

which implies that with probability at least $1-\epsilon$,

$$\inf_{\boldsymbol{\xi}:\|\boldsymbol{\xi}_{\mathcal{Q}^{*c}},\|_1\le 3\|\boldsymbol{\xi}_{\mathcal{Q}^*}\|_1,\mathbb{E}(\boldsymbol{U}^\intercal\boldsymbol{\xi})^2=1}\left\{\mathbb{P}_N(\boldsymbol{U}^\intercal\boldsymbol{\xi})^2-0.5\,\mathbb{E}(\boldsymbol{U}^\intercal\boldsymbol{\xi})^2\right\}\ge 0$$

□

Proof of Lemma S4. Similar to the proof of Lemma S2, we have

$$\Pr\left\{\left|\mathbb{P}_N(U_j^2)-\mathbb{E}(U_j^2)\right|\ge 4B^2\Big(\frac{2t}{N}\Big)^{1/2}+2B^2\frac{t}{N}\right\}\le e^{-t}.$$

Note that $\mathbb{E}\,U_j^2\le 4B^2$. Let $t=\log(p/\epsilon)$. Since $\log(p/\epsilon)\le N$, we have

$$\Pr\left[\left|\mathbb{P}_N(U_j^2)\right|\ge 16B^2 \text{ for some } j\right]\le\epsilon.$$

□

Proof of Lemma S5. In this proof, the expectation operator $\mathbb{E}$ is understood as the expectation with respect to the data $S,\boldsymbol{U}$ while treating $\widehat{\boldsymbol{\xi}}_{\text{init}}$ as a fixed quantity. All the following derivations are based on the event $\mathscr{T}_1\cap\mathscr{T}_2\cap\mathscr{T}_3$. Let

$$\tau_1=8B^2\Big\{\frac{\log(p/\epsilon)}{N}\Big\}^{1/2}.$$

We observe that

$$\begin{aligned}&\mathbb{P}_N(S-\boldsymbol{U}^\intercal\widehat{\boldsymbol{\xi}}_{\text{init}})^2-\mathbb{P}_N(S-\boldsymbol{U}^\intercal\boldsymbol{\xi}^*)^2\\&\quad=\mathbb{P}_N\{(S-\boldsymbol{U}^\intercal\boldsymbol{\xi}^*)+(\boldsymbol{U}^\intercal\boldsymbol{\xi}^*-\boldsymbol{U}^\intercal\widehat{\boldsymbol{\xi}}_{\text{init}})\}^2-\mathbb{P}_N(S-\boldsymbol{U}^\intercal\boldsymbol{\xi}^*)^2\\&\quad=\mathbb{P}_N(\boldsymbol{U}^\intercal\widehat{\boldsymbol{\xi}}_{\text{init}}-\boldsymbol{U}^\intercal\boldsymbol{\xi}^*)^2+\mathbb{P}_N(\widehat{\boldsymbol{\xi}}_{\text{init}}-\boldsymbol{\xi}^*)^\intercal\boldsymbol{U}(S-\boldsymbol{U}^\intercal\boldsymbol{\xi}^*).\end{aligned}$$

Since $\widehat{\boldsymbol{\xi}}_{\text{init}}$ is a minimizer, we have

$$\mathbb{P}_N(S-\boldsymbol{U}^\intercal\widehat{\boldsymbol{\xi}}_{\text{init}})^2+\mu_{\text{init}}\|\widehat{\boldsymbol{\xi}}_{\text{init},\mathcal{P}}\|_1\le\mathbb{P}_N(S-\boldsymbol{U}^\intercal\boldsymbol{\xi}^*)^2+\mu_{\text{init}}\|\boldsymbol{\xi}^*_{\mathcal{P}}\|_1.$$

Note that $\boldsymbol{\xi}^*_{\mathcal{Q}^*}=\boldsymbol{\xi}^*$ and $\boldsymbol{\xi}^*_{\mathcal{Q}^{*c}}=\boldsymbol{0}$. Thus we have,

$$\begin{aligned}&\mathbb{P}_N(\boldsymbol{U}^\intercal\widehat{\boldsymbol{\xi}}_{\text{init}}-\boldsymbol{U}^\intercal\boldsymbol{\xi}^*)^2\\&\quad\le-\mathbb{P}_N(\widehat{\boldsymbol{\xi}}_{\text{init}}-\boldsymbol{\xi}^*)^\intercal\boldsymbol{U}(S-\boldsymbol{U}^\intercal\boldsymbol{\xi}^*)+\mu_{\text{init}}\|\boldsymbol{\xi}^*_{\mathcal{P}}\|_1-\mu_{\text{init}}\|\widehat{\boldsymbol{\xi}}_{\text{init},\mathcal{P}}\|_1\\&\quad\le\tau_1\|\widehat{\boldsymbol{\xi}}_{\text{init}}-\boldsymbol{\xi}^*\|_1+\mu_{\text{init}}\|\boldsymbol{\xi}^*_{\mathcal{P}}\|_1-\mu_{\text{init}}\|\widehat{\boldsymbol{\xi}}_{\text{init},\mathcal{P}}\|_1\\&\quad=\tau_1\|\widehat{\boldsymbol{\xi}}_{\text{init},\mathcal{Q}^*}-\boldsymbol{\xi}^*\|_1+\tau_1\|\widehat{\boldsymbol{\xi}}_{\text{init},\mathcal{Q}^{*c}}\|_1+\mu_{\text{init}}\|\boldsymbol{\xi}^*_{\mathcal{P}}\|_1-\mu_{\text{init}}\|\widehat{\boldsymbol{\xi}}_{\text{init},\mathcal{P}\cap\mathcal{Q}^*}\|_1-\mu_{\text{init}}\|\widehat{\boldsymbol{\xi}}_{\text{init},\mathcal{P}\cap\mathcal{Q}^{*c}}\|_1\\&\quad\le\tau_1\|\widehat{\boldsymbol{\xi}}_{\text{init},\mathcal{Q}^*}-\boldsymbol{\xi}^*\|_1+\mu_{\text{init}}\|\widehat{\boldsymbol{\xi}}_{\text{init},\mathcal{P}\cap\mathcal{Q}^*}-\boldsymbol{\xi}^*_{\mathcal{P}}\|_1+\tau_1\|\widehat{\boldsymbol{\xi}}_{\text{init},\mathcal{Q}^{*c}}\|_1-\mu_{\text{init}}\|\widehat{\boldsymbol{\xi}}_{\text{init},\mathcal{P}\cap\mathcal{Q}^{*c}}\|_1\\&\quad\le(\mu_{\text{init}}+\tau_1)\|\widehat{\boldsymbol{\xi}}_{\text{init},\mathcal{Q}^*}-\boldsymbol{\xi}^*\|_1-(\mu_{\text{init}}-\tau_1)\|\widehat{\boldsymbol{\xi}}_{\text{init},\mathcal{Q}^{*c}}\|_1,\end{aligned}$$

where $\tau_1=8B^2\{\log(Np)/N\}^{1/2}$, in the second inequality we apply Lemma S2, and in the last inequality we use the relationship $\{p+1\}\subseteq\mathcal{Q}^*$. Since $\mathbb{P}_N(\boldsymbol{U}^\intercal\widehat{\boldsymbol{\xi}}_{\text{init}}-\boldsymbol{U}^\intercal\boldsymbol{\xi}^*)^2\ge 0$ and $\mu_{\text{init}}\ge 2\tau_1$, we know that $\widehat{\boldsymbol{\xi}}_{\text{init}}-\boldsymbol{\xi}^*\in\mathscr{C}$, where $\mathscr{C}=\{\boldsymbol{\xi}\in\mathbb{R}^{p+1}:\|\boldsymbol{\xi}_{\mathcal{Q}^{*c}}\|_1\le 3\|\boldsymbol{\xi}_{\mathcal{Q}^*}\|_1\}$.

The last chain of inequalities imply that

$$\mathbb{P}_N(\boldsymbol{U}^\intercal\widehat{\boldsymbol{\xi}}_{\text{init}} - \boldsymbol{U}^\intercal\boldsymbol{\xi}^*)^2 + (\mu_{\text{init}}/2)\|\widehat{\boldsymbol{\xi}}_{\text{init},\mathcal{Q}^{*c}}\|_1 \le (3\mu_{\text{init}}/2)\|\widehat{\boldsymbol{\xi}}_{\text{init},\mathcal{Q}^*} - \boldsymbol{\xi}^*\|_1,$$

and thus

$$\mathbb{P}_N(\boldsymbol{U}^\intercal\widehat{\boldsymbol{\xi}}_{\text{init}} - \boldsymbol{U}^\intercal\boldsymbol{\xi}^*)^2 + (\mu_{\text{init}}/2)\|\widehat{\boldsymbol{\xi}}_{\text{init}} - \boldsymbol{\xi}^*\|_1 \le 2\mu_{\text{init}}\|\widehat{\boldsymbol{\xi}}_{\text{init},\mathcal{Q}^*} - \boldsymbol{\xi}^*\|_1.$$

Note that $\widehat{\boldsymbol{\xi}}_{\text{init}} - \boldsymbol{\xi}^* \in \mathscr{C}$. Thus, by Lemma S3, we have

$$\mathbb{P}_N(\boldsymbol{U}^\intercal\widehat{\boldsymbol{\xi}}_{\text{init}} - \boldsymbol{U}^\intercal\boldsymbol{\xi}^*)^2 \ge (1/2)\,\mathbb{E}(\boldsymbol{U}^\intercal\widehat{\boldsymbol{\xi}}_{\text{init}} - \boldsymbol{U}^\intercal\boldsymbol{\xi}^*)^2.$$

By Cauchy-Schwartz inequality and assumption A4, we have

$$\|\widehat{\boldsymbol{\xi}}_{\text{init},\mathcal{Q}^*} - \boldsymbol{\xi}^*\|_1 \le (q^*)^{1/2}\|\widehat{\boldsymbol{\xi}}_{\text{init},\mathcal{Q}^*} - \boldsymbol{\xi}^*\|_2 \le \big\{q^*\,\mathbb{E}(\boldsymbol{U}^\intercal\widehat{\boldsymbol{\xi}}_{\text{init}} - \boldsymbol{U}^\intercal\boldsymbol{\xi}^*)^2/\varphi^2\big\}^{1/2}.$$

Combining these inequalities, we obtain

$$\begin{aligned}\mathbb{E}(\boldsymbol{U}^\intercal\widehat{\boldsymbol{\xi}}_{\text{init}} - \boldsymbol{U}^\intercal\boldsymbol{\xi}^*)^2 + \mu_{\text{init}}\|\widehat{\boldsymbol{\xi}}_{\text{init}} - \boldsymbol{\xi}^*\|_1 &\le 4\mu_{\text{init}}\|\widehat{\boldsymbol{\xi}}_{\text{init},\mathcal{Q}^*} - \boldsymbol{\xi}^*\|_1\\ &\le 4\mu_{\text{init}}\big\{q^*\,\mathbb{E}(\boldsymbol{U}^\intercal\widehat{\boldsymbol{\xi}}_{\text{init}} - \boldsymbol{U}^\intercal\boldsymbol{\xi}^*)^2/\varphi^2\big\}^{1/2}.\end{aligned}$$

By inequality $a^2/2 + b^2/2 \ge ab$, we have

$$\mathbb{E}(\boldsymbol{U}^\intercal\widehat{\boldsymbol{\xi}}_{\text{init}} - \boldsymbol{U}^\intercal\boldsymbol{\xi}^*)^2 + \mu_{\text{init}}\|\widehat{\boldsymbol{\xi}}_{\text{init}} - \boldsymbol{\xi}^*\|_1 \le \mathbb{E}(\boldsymbol{U}^\intercal\widehat{\boldsymbol{\xi}}_{\text{init}} - \boldsymbol{U}^\intercal\boldsymbol{\xi}^*)^2/2 + 8\mu_{\text{init}}^2 q^*/\varphi^2.$$

Consequently, we have

$$\mathbb{E}(\boldsymbol{U}^\intercal\widehat{\boldsymbol{\xi}}_{\text{init}} - \boldsymbol{U}^\intercal\boldsymbol{\xi}^*)^2 + 2\mu_{\text{init}}\|\widehat{\boldsymbol{\xi}}_{\text{init}} - \boldsymbol{\xi}^*\|_1 \le 16\mu_{\text{init}}^2 q^*/\varphi^2.$$

Therefore, we conclude

$$\|\widehat{\boldsymbol{\xi}}_{\text{init}} - \boldsymbol{\xi}^*\|_1 \le 8\mu_{\text{init}} q^*/\varphi^2.$$

□

Proof of Lemma S6. Let $\overline{w} = \max_{j\in\mathcal{Q}^*}\widehat{\omega}_j$. By the definition of weights,

$$\overline{w} = \max_{j\in\mathcal{Q}^*}\widehat{\omega}_j \le \{\min_{j\in\mathcal{Q}^*\cap\mathcal{P}}|(\widehat{\xi}_{\text{init}})_j|\}^{-\nu}.$$

On the event $\mathscr{T}_1\cap\mathscr{T}_2\cap\mathscr{T}_3$, we have $\|\widehat{\boldsymbol{\xi}}_{\text{init}} - \boldsymbol{\xi}^*\|_1 \le 8\mu_{\text{init}}q^*/\varphi^2$. By assumption A5, $\xi^*_{\min} \ge 16\mu_{\text{init}}q^*/\varphi^2$, we have that for any $j \in \mathcal{Q}^*\cap\mathcal{P}$, $|(\widehat{\xi}_{\text{init}})_j| \ge |\xi^*_j| - |(\widehat{\xi}_{\text{init}})_j - \xi^*_j| \ge \xi^*_{\min}/2$. Thus $\overline{w} \le (\xi^*_{\min}/2)^{-\nu}$.

The following arguments are in the same manner of those in Lemma S5. Let $\tau_1 = 8B^2\{\log(p/\epsilon)/N\}^{1/2}$. By assumption A7, we know $\mu\overline{w} \le \mu_{\text{init}}/2$ and $\tau_1 \le \mu_{\text{init}}/2$. Thus, on the event $\mathscr{T}_1\cap\mathscr{T}_2\cap\mathscr{T}_3$, we have

$$\begin{aligned}(1/2)\,\mathbb{E}(\boldsymbol{U}^\intercal\widetilde{\boldsymbol{\xi}} - \boldsymbol{U}^\intercal\boldsymbol{\xi}^*)^2 &\le \mathbb{P}_N(\boldsymbol{U}^\intercal\widetilde{\boldsymbol{\xi}} - \boldsymbol{U}^\intercal\boldsymbol{\xi}^*)^2 \le (\mu\overline{w} + \tau_1)\|\widetilde{\boldsymbol{\xi}} - \boldsymbol{\xi}^*\|_1\\ &\le \mu_{\text{init}}(q^*)^{1/2}\|\widetilde{\boldsymbol{\xi}} - \boldsymbol{\xi}^*\|_2 \le \mu_{\text{init}}(q^*)^{1/2}\{\mathbb{E}\|\boldsymbol{U}^\intercal\widetilde{\boldsymbol{\xi}} - \boldsymbol{U}^\intercal\boldsymbol{\xi}^*\|_2^2\}^{1/2}/\varphi\\ &\le (1/4)\,\mathbb{E}\|\boldsymbol{U}^\intercal\widetilde{\boldsymbol{\xi}} - \boldsymbol{U}^\intercal\boldsymbol{\xi}^*\|_2^2 + \mu_{\text{init}}^2 q^*/\varphi^2.\end{aligned}$$

Thus $\mathbb{E}(\boldsymbol{U}^\intercal\widetilde{\boldsymbol{\xi}} - \boldsymbol{U}^\intercal\boldsymbol{\xi}^*)^2 \le 4\mu_{\text{init}}^2 q^*/\varphi^2$. By assumption A4 and the relationship between L_1 and L_2 norms, we have

$$\|\widetilde{\boldsymbol{\xi}} - \boldsymbol{\xi}^*\|_1 \le (q^*)^{1/2}\|\widetilde{\boldsymbol{\xi}} - \boldsymbol{\xi}^*\|_2 \le 2\mu_{\text{init}} q^*/\varphi^2.$$

In addition, from the displayed inequalities above,

$$\mathbb{P}_N(\boldsymbol{U}^\intercal\widetilde{\boldsymbol{\xi}} - \boldsymbol{U}^\intercal\boldsymbol{\xi}^*)^2 \le (\mu\overline{w} + \tau_1)\|\widetilde{\boldsymbol{\xi}} - \boldsymbol{\xi}^*\|_1 \le 2\mu_{\text{init}}^2 q^*/\varphi^2.$$

And again by A5, for any $j \in \mathcal{Q}^* \cap \mathcal{P}$, we have

$$|\widetilde{\xi}_j| \ge |\xi_j^*| - |\widetilde{\xi} - \xi_j^*| \ge 16\mu_{\text{init}} q^*/\varphi^2 - \|\widetilde{\boldsymbol{\xi}} - \boldsymbol{\xi}^*\|_1 \ge 14\mu_{\text{init}} q^*/\varphi^2,$$

which implies that $\text{supp}(\widetilde{\boldsymbol{\xi}}) = \mathcal{Q}^*$.

□

Proof of Lemma S7. We will show that, on the event $\mathscr{T}_1 \cap \mathscr{T}_2 \cap \mathscr{T}_3$, we have $\widehat{\boldsymbol{\xi}} = \widetilde{\boldsymbol{\xi}}$. Then the L_1 error bound of $\widehat{\boldsymbol{\xi}}$ follows from that of $\widetilde{\boldsymbol{\xi}}$, and the support of $\widehat{\boldsymbol{\xi}}$ is the same as that of $\widetilde{\boldsymbol{\xi}}$ which is $\mathcal{Q}^*$.

Using Lemma 3 of Tibshirani et al. (2013), by Assumption (A4) (general position for $\boldsymbol{K}$), $\widehat{\boldsymbol{\xi}}$ is the unique solution to its (adaptive) lasso problem. Then by Karush-Kuhn-Tucker conditions, it suffices to show

$$|2\,\mathbb{P}_N U_j(S - \boldsymbol{U}^\intercal\widetilde{\boldsymbol{\xi}})| \le \mu\widehat{\omega}_j$$

for all j. When $j \in \mathcal{Q}^*$, this inequality automatically holds by the definition of $\widetilde{\boldsymbol{\xi}}$. When $j \in \mathcal{Q}^{*c}$, note that

$$|\mathbb{P}_N U_j(S - \boldsymbol{U}^\intercal\widetilde{\boldsymbol{\xi}})| \le |\mathbb{P}_N U_j(S - \boldsymbol{U}^\intercal\boldsymbol{\xi}^*)| + |\mathbb{P}_N U_j(\boldsymbol{U}^\intercal\widetilde{\boldsymbol{\xi}} - \boldsymbol{U}^\intercal\boldsymbol{\xi}^*)|.$$

For the first term, by Lemma S2, $|\mathbb{P}_N U_j(S - \boldsymbol{U}^\intercal\boldsymbol{\xi}^*)| \le \tau_1 \le \mu_{\text{init}}/2$ for all j, where $\tau_1 = 8B^2\{\log(Np)/N\}^{1/2}$. For the second term, by Cauchy-Schwarz inequality, we have

$$|\mathbb{P}_N U_j(\boldsymbol{U}^\intercal\widetilde{\boldsymbol{\xi}} - \boldsymbol{U}^\intercal\boldsymbol{\xi}^*)| \le (\mathbb{P}_N U_j^2)^{1/2}\{\mathbb{P}_N(\boldsymbol{U}^\intercal\widetilde{\boldsymbol{\xi}} - \boldsymbol{U}^\intercal\boldsymbol{\xi}^*)^2\}^{1/2} \le 8B\mu_{\text{init}}(q^*/\varphi^2)^{1/2}.$$

Thus, when $j \in \mathcal{Q}^{*c}$, we have

$$|\mathbb{P}_N U_j(S - \boldsymbol{U}^\intercal\widetilde{\boldsymbol{\xi}})| \le \mu_{\text{init}}\{1 + 8B(q^*/\varphi^2)^{1/2}\}.$$

By the definition of $\widehat{\omega}$, we have

$$\max_{j\in\mathcal{Q}^{*c}} |(\widehat{\xi}_{\text{init}})_j| \le \|\widehat{\boldsymbol{\xi}}_{\text{init}} - \boldsymbol{\xi}^*\|_1 \le 8\mu_{\text{init}} q^*/\varphi^2.$$

Hence by assumption A7, we know $|2\,\mathbb{P}_N U_j(S - \boldsymbol{U}^\intercal\widetilde{\boldsymbol{\xi}})| \le \mu\widehat{\omega}_j$ for every $j \in \mathcal{Q}^{*c}$. □

Proof of Lemma S10. Throughout this proof, for notation simplicity we shall write $\boldsymbol{Z} = \boldsymbol{Z}_{\boldsymbol{\alpha}^*}$. Let $\boldsymbol{v} = \boldsymbol{\theta} - \boldsymbol{\theta}_0$. Define random variable

$$V(t) = -tY\boldsymbol{Z}^\intercal\boldsymbol{v} + \log\{1+\exp(\boldsymbol{Z}^\intercal\boldsymbol{\theta}_0 + t\boldsymbol{Z}^\intercal\boldsymbol{v})\} - \log\{1+\exp(\boldsymbol{Z}^\intercal\boldsymbol{\theta}_0)\}.$$

By definition, we have $V(0) = 0$ and

$$L_0(\boldsymbol{\theta}_0 + \boldsymbol{v}) - L_0(\boldsymbol{\theta}_0) = \mathbb{E}\{V(1)\}.$$

Let $g(x) = e^x/(1+e^x)$. Direct calculation gives

$$\begin{aligned} V'(t) &= -Y\boldsymbol{Z}^\intercal\boldsymbol{v} + g(\boldsymbol{Z}^\intercal\boldsymbol{\theta}_0 + t\boldsymbol{Z}^\intercal\boldsymbol{v})(\boldsymbol{Z}^\intercal\boldsymbol{v}), \\ V''(t) &= g(\boldsymbol{Z}^\intercal\boldsymbol{\theta}_0 + t\boldsymbol{Z}^\intercal\boldsymbol{v})\{1 - g(\boldsymbol{Z}^\intercal\boldsymbol{\theta}_0 + t\boldsymbol{Z}^\intercal\boldsymbol{v})\}(\boldsymbol{Z}^\intercal\boldsymbol{v})^2, \\ V^{(3)}(t) &= g(\boldsymbol{Z}^\intercal\boldsymbol{\theta}_0 + t\boldsymbol{Z}^\intercal\boldsymbol{v})\{1 - g(\boldsymbol{Z}^\intercal\boldsymbol{\theta}_0 + t\boldsymbol{Z}^\intercal\boldsymbol{v})\}\{1 - 2g(\boldsymbol{Z}^\intercal\boldsymbol{\theta}_0 + t\boldsymbol{Z}^\intercal\boldsymbol{v})\}(\boldsymbol{Z}^\intercal\boldsymbol{v})^3. \end{aligned}$$

Since $\mathbb{E}(Y \mid \boldsymbol{Z}) = g(\boldsymbol{Z}^\intercal\boldsymbol{\theta}_0)$, we have $\mathbb{E}\{V'(0)\} = 0$. By definition of $\boldsymbol{H}$, we have $\mathbb{E}\{V''(0)\} = \boldsymbol{v}^\intercal\boldsymbol{H}\boldsymbol{v}$. Moreover, for any $t \in \mathbb{R}$ we have $\mathbb{E}|V^{(3)}(t)| \le \{\sup_{x\in[0,1]} x(1-x)(1-2x)\}\,\mathbb{E}|\boldsymbol{Z}^\intercal\boldsymbol{v}|^3 \le \mathbb{E}|\boldsymbol{Z}^\intercal\boldsymbol{v}|^3/10$.

By Taylor expansion, we have

$$V(t) - V(0) = V'(0)t + \frac{1}{2}V''(0)t^2 + \frac{1}{6}V^{(3)}(\xi)t^3.$$

for some random variable $\xi \in [0, t]$. Plugging in $t = 1$ and taking expectation on both side, we obtain

$$\mathbb{E}\{V(1)\} \ge \frac{1}{2}\boldsymbol{v}^\intercal\boldsymbol{H}\boldsymbol{v} - \frac{1}{60}\,\mathbb{E}|\boldsymbol{Z}^\intercal\boldsymbol{v}|^3.$$

Applying assumption A4 to obtain

$$\mathbb{E}\{V(1)\} \ge \frac{1}{4}\boldsymbol{v}^\intercal\boldsymbol{H}\boldsymbol{v} + \frac{\varpi}{4}\,\mathbb{E}(\boldsymbol{Z}^\intercal\boldsymbol{v})^2 - \frac{1}{60}\,\mathbb{E}|\boldsymbol{Z}^\intercal\boldsymbol{v}|^3.$$

Since

$$\{\mathbb{E}(\boldsymbol{Z}^\intercal\boldsymbol{v})^2\}^{1/2} = \|\boldsymbol{G}^{1/2}\boldsymbol{v}\|_2 \le \varpi^{-1/2}\|\boldsymbol{H}^{1/2}\boldsymbol{v}\|_2 \le \varpi^{-1/2}\eta$$

and by A3

$$\varpi^{-1/2}\eta \le \frac{15\varpi\{\mathbb{E}(\boldsymbol{Z}^\intercal\boldsymbol{v})^2\}^{3/2}}{\mathbb{E}|\boldsymbol{Z}^\intercal\boldsymbol{v}|^3},$$

we have

$$\frac{\varpi}{4}\,\mathbb{E}(\boldsymbol{Z}^\intercal\boldsymbol{v})^2 - \frac{1}{60}\,\mathbb{E}|\boldsymbol{Z}^\intercal\boldsymbol{v}|^3 \ge 0.$$

Therefore

$$L_0(\boldsymbol{\theta}_0 + \boldsymbol{v}) - L_0(\boldsymbol{\theta}_0) = \mathbb{E}\{V(1)\} \ge \frac{1}{4}\boldsymbol{v}^\intercal\boldsymbol{H}\boldsymbol{v}.$$

Similarly,

$$\mathbb{E}\{V(1)\} \le \frac{1}{2}\boldsymbol{v}^\intercal\boldsymbol{H}\boldsymbol{v} + \frac{1}{60}\,\mathbb{E}|\boldsymbol{Z}^\intercal\boldsymbol{v}|^3 \le \frac{3}{4}\boldsymbol{v}^\intercal\boldsymbol{H}\boldsymbol{v} - \frac{\varpi}{4}\,\mathbb{E}(\boldsymbol{Z}^\intercal\boldsymbol{v})^2 + \frac{1}{60}\,\mathbb{E}|\boldsymbol{Z}^\intercal\boldsymbol{v}|^3.$$

Therefore

$$L_0(\boldsymbol{\theta}_0 + \boldsymbol{v}) - L_0(\boldsymbol{\theta}_0) = \mathbb{E}\{V(1)\} \le \frac{3}{4}\boldsymbol{v}^\intercal\boldsymbol{H}\boldsymbol{v}.$$

□

Proof of Lemma S11. By Lemma S10, we have

$$\begin{aligned}\mathbb{E}\,\ell(Y, \boldsymbol{Z}_{\widehat{\boldsymbol{\alpha}}}^{\intercal}\widehat{\boldsymbol{\theta}}) - \mathbb{E}\,\ell(Y, \boldsymbol{Z}_{\boldsymbol{\alpha}^*}^{\intercal}\boldsymbol{\theta}_0) &= L_0(\widehat{\Psi}_{+}(\widehat{\boldsymbol{\theta}})) - L_0(\boldsymbol{\theta}_0)\\ &\le \|\boldsymbol{H}^{1/2}(\widehat{\Psi}_{+}(\widehat{\boldsymbol{\theta}}) - \boldsymbol{\theta}_0)\|_2^2.\end{aligned}$$

By assumption A4, we have

$$\begin{aligned}\mathbb{E}(\boldsymbol{Z}_{\widehat{\boldsymbol{\alpha}}}^{\intercal}\widehat{\boldsymbol{\theta}} - \boldsymbol{Z}_{\boldsymbol{\alpha}^*}^{\intercal}\boldsymbol{\theta}_0)^2 &= \|\boldsymbol{G}^{1/2}(\widehat{\Psi}_{+}(\widehat{\boldsymbol{\theta}}) - \boldsymbol{\theta}_0)\|_2^2\\ &\le \|\boldsymbol{H}^{1/2}(\widehat{\Psi}_{+}(\widehat{\boldsymbol{\theta}}) - \boldsymbol{\theta}_0)\|_2^2/\varpi.\end{aligned}$$

Since $\sigma(t) = e^t/(1+e^t)$, $\sigma(t)$ is 1-Lipschitz. Hence

$$\begin{aligned}\mathbb{E}\{\sigma(\boldsymbol{Z}_{\widehat{\boldsymbol{\alpha}}}^{\intercal}\widehat{\boldsymbol{\theta}}) - \sigma(\boldsymbol{Z}_{\boldsymbol{\alpha}^*}^{\intercal}\boldsymbol{\theta}_0)\}^2 &= \mathbb{E}\{\sigma(\boldsymbol{Z}_{\widehat{\boldsymbol{\alpha}}}^{\intercal}\widehat{\boldsymbol{\theta}}) - \sigma(\boldsymbol{Z}_{\boldsymbol{\alpha}^*}^{\intercal}\boldsymbol{\theta}_0)\}^2\\ &\le \mathbb{E}(\boldsymbol{Z}_{\widehat{\boldsymbol{\alpha}}}^{\intercal}\widehat{\boldsymbol{\theta}} - \boldsymbol{Z}_{\boldsymbol{\alpha}^*}^{\intercal}\boldsymbol{\theta}_0)^2 \le \|\boldsymbol{H}^{1/2}(\widehat{\Psi}_{+}(\widehat{\boldsymbol{\theta}}) - \boldsymbol{\theta}_0)\|_2^2/\varpi.\end{aligned}$$

□

Proof of Lemma S12. Denote by $F_n(\boldsymbol{\theta}) = \mathbb{P}_n\,\ell(Y, \boldsymbol{Z}_{\widehat{\boldsymbol{\alpha}}}^{\intercal}\boldsymbol{\theta}) + \Omega(\boldsymbol{\theta}_{\mathcal{P}})$. Since $\widehat{\boldsymbol{\theta}}$ is a minimizer of $F_n(\boldsymbol{\theta})$, then $\breve{\boldsymbol{\theta}} = \widehat{\Psi}_{+}(\widehat{\boldsymbol{\theta}})$ is a minimizer of $L_n(\boldsymbol{\theta}) + \Omega(\widehat{\Psi}_{-}(\boldsymbol{\theta})_{\mathcal{P}})$. Thus we have

$$L_n(\breve{\boldsymbol{\theta}}) + \Omega(\widehat{\Psi}_{-}(\breve{\boldsymbol{\theta}})_{\mathcal{P}}) \le L_n(\boldsymbol{\theta}^*) + \Omega(\widehat{\Psi}_{-}(\boldsymbol{\theta}^*)_{\mathcal{P}}).$$

Since $L_n(\boldsymbol{\theta}) + \Omega(\widehat{\Psi}_{-}(\boldsymbol{\theta})_{\mathcal{P}})$ is a convex function of $\boldsymbol{\theta}$ and $\widetilde{\boldsymbol{\theta}} = k\breve{\boldsymbol{\theta}} + (1-k)\boldsymbol{\theta}^*$, we have

$$\begin{aligned}L_n(\widetilde{\boldsymbol{\theta}}) + \Omega(\widehat{\Psi}_{-}(\widetilde{\boldsymbol{\theta}})_{\mathcal{P}}) &\le k\big\{L_n(\breve{\boldsymbol{\theta}}) + \Omega(\widehat{\Psi}_{-}(\breve{\boldsymbol{\theta}})_{\mathcal{P}})\big\} + (1-k)\big\{L_n(\boldsymbol{\theta}^*) + \Omega(\widehat{\Psi}_{-}(\boldsymbol{\theta}^*)_{\mathcal{P}})\big\}\\ &\le L_n(\boldsymbol{\theta}^*) + \Omega(\widehat{\Psi}_{-}(\boldsymbol{\theta}^*)_{\mathcal{P}}).\end{aligned}$$

Note that for any $\boldsymbol{\theta}$,

$$|\Omega(\widehat{\Psi}_{-}(\boldsymbol{\theta})_{\mathcal{P}}) - \Omega(\boldsymbol{\theta}_{\mathcal{P}})| \le \Omega(\widehat{\Psi}_{-}(\boldsymbol{\theta})_{\mathcal{P}} - \boldsymbol{\theta}_{\mathcal{P}}) = \lambda_1|\rho|\|\widehat{\boldsymbol{\alpha}} - \boldsymbol{\alpha}^*\|_1 \le \lambda_1\Delta_{\boldsymbol{\alpha}}|\rho|.$$

So we have

$$\begin{aligned}\Omega(\widehat{\Psi}_{-}(\widetilde{\boldsymbol{\theta}})_{\mathcal{P}}) &\ge \Omega(\widetilde{\boldsymbol{\theta}}_{\mathcal{P}}) - \lambda_1\Delta_{\boldsymbol{\alpha}}|\widetilde{\rho}|,\\ \Omega(\widehat{\Psi}_{-}(\boldsymbol{\theta}^*)_{\mathcal{P}}) &\le \Omega(\boldsymbol{\theta}^*_{\mathcal{P}}) + \lambda_1\Delta_{\boldsymbol{\alpha}}|\rho^*|.\end{aligned}$$

Hence we get

$$L_n(\widetilde{\boldsymbol{\theta}}) + \Omega(\widetilde{\boldsymbol{\theta}}_{\mathcal{P}}) - \lambda_1\Delta_{\boldsymbol{\alpha}}|\widetilde{\rho}| \le L_n(\boldsymbol{\theta}^*) + \Omega(\boldsymbol{\theta}^*_{\mathcal{P}}) + \lambda_1\Delta_{\boldsymbol{\alpha}}|\rho^*|.$$

Since $|\widetilde{\rho}| \le |\rho^*| + |\widetilde{\rho} - \rho^*|$,

$$L_n(\widetilde{\boldsymbol{\theta}}) + \Omega(\widetilde{\boldsymbol{\theta}}_{\mathcal{P}}) \le L_n(\boldsymbol{\theta}^*) + \Omega(\boldsymbol{\theta}^*_{\mathcal{P}}) + 2\lambda_1\Delta_{\boldsymbol{\alpha}}|\rho^*| + \lambda_1\Delta_{\boldsymbol{\alpha}}|\widetilde{\rho} - \rho^*|.$$

Finally, under A7 and the range of λ_0, λ_1,we know $\lambda_1\Delta_{\boldsymbol{\alpha}} \le \lambda_0/4$. Hence we have

$$\lambda_1\Delta_{\boldsymbol{\alpha}}|\widetilde{\rho} - \rho^*| \le \frac{1}{4}\lambda_0|\widetilde{\rho} - \rho^*| \le \frac{1}{4}\Omega(\widetilde{\boldsymbol{\theta}}_{\mathcal{S}_{+}^*} - \boldsymbol{\theta}^*_{\mathcal{S}_{+}^*}).$$

□

Proof of Lemma S13. Note that $\mathcal{S}^*_- \subseteq \mathcal{P}$. We have

$$\begin{aligned}
&\Omega(\boldsymbol{\theta}^*_{\mathcal{P}}) - \Omega(\widetilde{\boldsymbol{\theta}}_{\mathcal{P}}) + k\Omega(\widetilde{\boldsymbol{\theta}} - \boldsymbol{\theta}^*) \\
&\quad = \Omega(\boldsymbol{\theta}^*_{\mathcal{P}\cap\mathcal{S}^*_+}) + \Omega(\boldsymbol{\theta}^*_{\mathcal{S}^*_-}) - \Omega(\widetilde{\boldsymbol{\theta}}_{\mathcal{P}\cap\mathcal{S}^*_+}) - \Omega(\widetilde{\boldsymbol{\theta}}_{\mathcal{S}^*_-}) - \Omega(\widetilde{\boldsymbol{\theta}}_{\mathcal{S}^{*c}}) \\
&\qquad + k\Omega(\widetilde{\boldsymbol{\theta}}_{\mathcal{S}^*_+} - \boldsymbol{\theta}^*_{\mathcal{S}^*_+}) + k\Omega(\widetilde{\boldsymbol{\theta}}_{\mathcal{S}^*_-} - \boldsymbol{\theta}^*_{\mathcal{S}^*_-}) + k\Omega(\widetilde{\boldsymbol{\theta}}_{\mathcal{S}^{*c}}).
\end{aligned}$$

Since

$$\begin{aligned}
\Omega(\boldsymbol{\theta}^*_{\mathcal{P}\cap\mathcal{S}^*_+}) - \Omega(\widetilde{\boldsymbol{\theta}}_{\mathcal{P}\cap\mathcal{S}^*_+}) &\le \Omega(\widetilde{\boldsymbol{\theta}}_{\mathcal{P}\cap\mathcal{S}^*_+} - \boldsymbol{\theta}^*_{\mathcal{P}\cap\mathcal{S}^*_+}) \le \Omega(\widetilde{\boldsymbol{\theta}}_{\mathcal{S}^*_+} - \boldsymbol{\theta}^*_{\mathcal{S}^*_+}), \\
\Omega(\boldsymbol{\theta}^*_{\mathcal{S}^*_-}) - \Omega(\widetilde{\boldsymbol{\theta}}_{\mathcal{S}^*_-}) &\le -\Omega(\widetilde{\boldsymbol{\theta}}_{\mathcal{S}^*_-} - \boldsymbol{\theta}^*_{\mathcal{S}^*_-}) + 2\Omega(\boldsymbol{\theta}^*_{\mathcal{S}^*_-}),
\end{aligned}$$

we obtain

$$\begin{aligned}
&\Omega(\boldsymbol{\theta}^*_{\mathcal{P}}) - \Omega(\widetilde{\boldsymbol{\theta}}_{\mathcal{P}}) + k\Omega(\widetilde{\boldsymbol{\theta}} - \boldsymbol{\theta}^*) \\
&\qquad \le (1+k)\Omega(\widetilde{\boldsymbol{\theta}}_{\mathcal{S}^*_+} - \boldsymbol{\theta}^*_{\mathcal{S}^*_+}) - (1-k)\Omega(\widetilde{\boldsymbol{\theta}}_{\mathcal{S}^*_-} - \boldsymbol{\theta}^*_{\mathcal{S}^*_-}) - (1-k)\Omega(\widetilde{\boldsymbol{\theta}}_{\mathcal{S}^{*c}}) + 2\Omega(\boldsymbol{\theta}^*_{\mathcal{S}^*_-}).
\end{aligned}$$

The presented inequality follows from $\Omega(\widetilde{\boldsymbol{\theta}}_{\mathcal{S}^{*c}}) = \Omega(\widetilde{\boldsymbol{\theta}}_{\mathcal{S}^{*c}} - \boldsymbol{\theta}^*_{\mathcal{S}^{*c}})$. □

Proof of Lemma S14. By the properties of ℓ_1-norm and ℓ_2-norm, we have $\|\boldsymbol{v}_{\mathcal{S}^*_+}\|_1 \le |\mathcal{S}^*_+|^{1/2}\|\boldsymbol{v}_{\mathcal{S}^*_+}\|_2$. By assumption (A6), $\|\boldsymbol{v}_{\mathcal{S}^*_+}\|_2 \le \phi^{-1/2}\|\boldsymbol{G}^{1/2}\boldsymbol{v}\|_2$. By assumption (A2), $\|\boldsymbol{G}^{1/2}\boldsymbol{v}\|_2 \le \varpi^{-1/2}\|\boldsymbol{H}^{1/2}\boldsymbol{v}\|_2$. Hence the first inequality holds. We observe that $\Omega(\boldsymbol{v}_{\mathcal{S}^*_+}) \le \kappa^*\|\boldsymbol{v}_{\mathcal{S}^*_+}\|_1$. So the second inequality holds.

□

Proof of Lemma S15. By the convexity of L_n,

$$L_n(\widetilde{\boldsymbol{\theta}}) - L_n(\boldsymbol{\theta}^*) \ge \left\{\frac{\partial L_n(\boldsymbol{\theta}^*)}{\partial \boldsymbol{\theta}}\right\}^{\mathsf{T}} (\widetilde{\boldsymbol{\theta}} - \boldsymbol{\theta}^*),$$

where

$$\frac{\partial L_n(\boldsymbol{\theta}^*)}{\partial \boldsymbol{\theta}} = \mathbb{P}_n G\boldsymbol{Z}_{\boldsymbol{\alpha}^*}, \quad G = -Y + \sigma(\boldsymbol{Z}^{\mathsf{T}}_{\boldsymbol{\alpha}^*}\boldsymbol{\theta}^*), \quad \sigma(t) = e^t/(1+e^t).$$

We observe that

$$G\boldsymbol{Z}^{\mathsf{T}}_{\boldsymbol{\alpha}^*}(\widetilde{\boldsymbol{\theta}} - \boldsymbol{\theta}^*) = G\boldsymbol{X}^{\mathsf{T}}(\widetilde{\boldsymbol{\delta}} - \boldsymbol{\delta}^*) + G\boldsymbol{X}^{\mathsf{T}}\boldsymbol{\alpha}^*(\widetilde{\rho} - \rho^*) + GS(\widetilde{\gamma} - \gamma^*) + G(\widetilde{\zeta} - \zeta^*).$$

For any $j \in \mathcal{P}$, GX_j is sub-Gaussian(B^2). Besides, $G\boldsymbol{X}^{\mathsf{T}}\boldsymbol{\alpha}^*$, GS, and G are also sub-Gaussian(B^2). By the property of sub-Gaussian random variables, with probability $1 - 3\varepsilon$, the three inequalities below hold simultaneously:

$$\begin{aligned}
\|\mathbb{P}_n G\boldsymbol{X}_{\mathcal{A}^*}\|_\infty &\le 2B\Lambda(n, q^*, \epsilon) \le \lambda_1/4, \\
\|\mathbb{P}_n G\boldsymbol{X}\|_\infty &\le 2B\Lambda(n, p, \epsilon) \le \lambda_2/4, \\
\max\{|\mathbb{P}_n G\boldsymbol{X}^{\mathsf{T}}\boldsymbol{\alpha}^*|, |\mathbb{P}_n GS|, |\mathbb{P}_n G|\} &\le 2B\Lambda(n, 3, \epsilon) \le \lambda_0/4.
\end{aligned}$$

Therefore we have

$$|G\boldsymbol{Z}_{\boldsymbol{\alpha}^*}^\intercal(\widetilde{\boldsymbol{\theta}}-\boldsymbol{\theta}^*)| \le \frac{\lambda_1}{4}\|(\widetilde{\boldsymbol{\delta}}-\boldsymbol{\delta}^*)_{\mathcal{A}^*}\|_1 + \frac{\lambda_2}{4}\|(\widetilde{\boldsymbol{\delta}}-\boldsymbol{\delta}^*)_{\mathcal{P}\setminus\mathcal{A}^*}\|_1 + \frac{\lambda_0}{4}(|\widetilde{\rho}-\rho^*|+|\widetilde{\gamma}-\gamma^*|+|\widetilde{\zeta}-\zeta^*|) \le \frac{1}{4}\Omega(\widetilde{\boldsymbol{\theta}}-\boldsymbol{\theta}^*)$$

by the definition of Ω. By the condition of $\widetilde{\boldsymbol{\theta}}$, we know

$$L_n(\widetilde{\boldsymbol{\theta}}) - L_n(\boldsymbol{\theta}^*) \le \Omega(\boldsymbol{\theta}^*_{\mathcal{P}}) - \Omega(\widetilde{\boldsymbol{\theta}}_{\mathcal{P}}) + \frac{1}{4}\Omega(\widetilde{\boldsymbol{\theta}}_{\mathcal{S}^*_+} - \boldsymbol{\theta}^*_{\mathcal{S}^*_+}) + 2\lambda_1\Delta_{\boldsymbol{\alpha}}|\rho^*|.$$

Since

$$L_n(\widetilde{\boldsymbol{\theta}}) - L_n(\boldsymbol{\theta}^*) \ge \mathbb{P}_n G\boldsymbol{Z}_{\boldsymbol{\alpha}^*}^\intercal(\widetilde{\boldsymbol{\theta}}-\boldsymbol{\theta}_0) \ge -\frac{1}{4}\Omega(\widetilde{\boldsymbol{\theta}}-\boldsymbol{\theta}^*),$$

we have

$$0 \le \Omega(\boldsymbol{\theta}^*_{\mathcal{P}}) - \Omega(\widetilde{\boldsymbol{\theta}}_{\mathcal{P}}) + \frac{1}{4}\Omega(\widetilde{\boldsymbol{\theta}}-\boldsymbol{\theta}^*) + \frac{1}{4}\Omega(\widetilde{\boldsymbol{\theta}}_{\mathcal{S}^*_+} - \boldsymbol{\theta}^*_{\mathcal{S}^*_+}) + 2\lambda_1\Delta_{\boldsymbol{\alpha}}|\rho^*|.$$

By Lemma S13 with $k = 1/4$, we have

$$\begin{aligned} 0 &\le \frac{5}{4}\Omega(\widetilde{\boldsymbol{\theta}}_{\mathcal{S}^*_+} - \boldsymbol{\theta}^*_{\mathcal{S}^*_+}) - \frac{3}{4}\Omega(\widetilde{\boldsymbol{\theta}}_{\mathcal{S}^{*c}_+} - \boldsymbol{\theta}^*_{\mathcal{S}^{*c}_+}) + 2\Omega(\boldsymbol{\theta}^*_{\mathcal{S}^*_-}) + \frac{1}{4}\Omega(\widetilde{\boldsymbol{\theta}}_{\mathcal{S}^*_+} - \boldsymbol{\theta}^*_{\mathcal{S}^*_+}) + 2\lambda_1\Delta_{\boldsymbol{\alpha}}|\rho^*| \\ &= \frac{3}{2}\Omega(\widetilde{\boldsymbol{\theta}}_{\mathcal{S}^*_+} - \boldsymbol{\theta}^*_{\mathcal{S}^*_+}) - \frac{3}{4}\Omega(\widetilde{\boldsymbol{\theta}}_{\mathcal{S}^{*c}_+} - \boldsymbol{\theta}^*_{\mathcal{S}^{*c}_+}) + 2\Omega(\boldsymbol{\theta}^*_{\mathcal{S}^*_-}) + 2\lambda_1\Delta_{\boldsymbol{\alpha}}|\rho^*|. \end{aligned}$$

□

Before proving Lemma S16, we need to first establish a lemma that establishes an upper bound for the exponential of the maximum of p sub-Gaussian random variables, the proof of which is given at the end of this section.

Lemma S18. *Let $X_1, \ldots, X_p$ be sub-Gaussian(B^2) and $O = \max_{1\le j\le p}|X_j|$. Then*

$$\mathbb{E}(e^O) \le 2\exp\left[\{2B^2\log(2p)\}^{1/2} + B^2\right].$$

Proof of Lemma S16. Let

$$V_{\boldsymbol{\theta}} = \ell(Y, \boldsymbol{Z}_{\boldsymbol{\alpha}^*}^\intercal\boldsymbol{\theta}) - \ell(Y, \boldsymbol{Z}_{\boldsymbol{\alpha}^*}^\intercal\boldsymbol{\theta}^*), \qquad U_{\boldsymbol{\theta}} = \boldsymbol{Z}_{\boldsymbol{\alpha}^*}^\intercal\boldsymbol{\theta} - \boldsymbol{Z}_{\boldsymbol{\alpha}^*}^\intercal\boldsymbol{\theta}^*.$$

Then $\mathbb{G}_n = \sup_{\boldsymbol{\theta}\in\mathscr{M}}|\mathbb{P}_n V_{\boldsymbol{\theta}} - \mathbb{E}\, V_{\boldsymbol{\theta}}|$.

Fix $t > 0$. We want to compute $\mathbb{E}\{\exp(t\mathbb{G}_n)\} = \mathbb{E}\{\exp(t\sup_{\boldsymbol{\theta}\in\mathscr{M}}|\mathbb{P}_n V_{\boldsymbol{\theta}} - \mathbb{E}\, V_{\boldsymbol{\theta}}|\}$. Observing that $h(x) = e^{tx}$ is an convex and increasing function of x, by symmetrization inequality (Koltchinskii, 2011, Theorem 2.1), we have

$$\mathbb{E}\{\exp(t\sup_{\boldsymbol{\theta}\in\mathscr{M}}|\mathbb{P}_n V_{\boldsymbol{\theta}} - \mathbb{E}\, V_{\boldsymbol{\theta}}|)\} \le \mathbb{E}\{\exp(2t\sup_{\boldsymbol{\theta}\in\mathscr{M}}|\mathbb{P}_n \varepsilon V_{\boldsymbol{\theta}}|)\},$$

where $\varepsilon_1, \ldots, \varepsilon_n$ are i.i.d. Rademancher random variables and $\varepsilon_1, \ldots, \varepsilon_n$ are independent of all the $V_{\boldsymbol{\theta}}$'s. Then, by the Lipschitz property of ℓ and the comparison inequality for Rademancher sums (Koltchinskii, 2011, Theorem 2.2), we have

$$\mathbb{E}\{\exp(2t\sup_{\boldsymbol{\theta}\in\mathscr{M}}|\mathbb{P}_n \varepsilon V_{\boldsymbol{\theta}}|)\} \le \mathbb{E}\{\exp(4t\sup_{\boldsymbol{\theta}\in\mathscr{M}}|\mathbb{P}_n \varepsilon U_{\boldsymbol{\theta}}|)\}.$$

Recall that $U_{\boldsymbol{\theta}} = \boldsymbol{Z}_{\boldsymbol{\alpha}^*}^\top(\boldsymbol{\theta} - \boldsymbol{\theta}^*)$. Let

$$I_1 = \mathbb{P}_n \varepsilon \boldsymbol{X}_{\mathcal{A}^*}^\top(\boldsymbol{\delta} - \boldsymbol{\delta}^*)_{\mathcal{A}^*},$$
$$I_2 = \mathbb{P}_n \varepsilon \boldsymbol{X}_{\mathcal{P}\setminus\mathcal{A}^*}^\top(\boldsymbol{\delta} - \boldsymbol{\delta}^*)_{\mathcal{P}\setminus\mathcal{A}^*},$$
$$I_3 = \mathbb{P}_n\{\varepsilon \boldsymbol{X}^\top \boldsymbol{\alpha}^*(\rho - \rho^*) + \varepsilon S(\gamma - \gamma^*) + \varepsilon(\zeta - \zeta^*)\}.$$

By the definition of $\boldsymbol{Z}_{\boldsymbol{\alpha}^*}$, we observe that

$$\mathbb{P}_n \varepsilon \boldsymbol{Z}_{\boldsymbol{\alpha}^*}^\top(\boldsymbol{\theta} - \boldsymbol{\theta}^*) = I_1 + I_2 + I_3.$$

Moreover, we see that $|I_k| \le J_k$ for all $k = 1, 2, 3$, where

$$J_1 = \|\mathbb{P}_n \varepsilon \boldsymbol{X}_{\mathcal{A}^*}\|_\infty M_1,$$
$$J_2 = \|\mathbb{P}_n \varepsilon \boldsymbol{X}_{\mathcal{P}\setminus\mathcal{A}^*}\|_\infty M_2,$$
$$J_3 = \max\{|\mathbb{P}_n \varepsilon \boldsymbol{X}^\top \boldsymbol{\alpha}^*|, |\mathbb{P}_n \varepsilon S|, |\mathbb{P}_n \varepsilon|\} M_0.$$

By Jensen's inequality, we have

$$\mathbb{E}\{\sup_{\boldsymbol{\theta}} \exp(4t \sup_{\boldsymbol{\theta}\in\mathscr{M}} |\mathbb{P}_n \varepsilon U_{\boldsymbol{\theta}}|)\} \le \mathbb{E}\{\exp(4t \sum_{k=1}^3 J_k)\} \le \frac{1}{3}\sum_{k=1}^3 \mathbb{E}\{\exp(12tJ_k)\}.$$

We know that for any $j \in \mathcal{P}$, $\mathbb{P}_n \varepsilon X_j$ is sub-Gaussian(B^2/n). Besides, $\mathbb{P}_n \varepsilon \boldsymbol{X}^\top \boldsymbol{\alpha}^*$, $\mathbb{P}_n \varepsilon S$ and $\mathbb{P}_n \varepsilon$ are also sub-Gaussian(B^2/n). Note that $|\mathcal{A}^*| = q^*$ and $|\mathcal{P}| = p$. Hence we have, by Lemma S18,

$$\mathbb{E}\{\exp(12tJ_1)\} \le 2\exp\left[\{288t^2B^2M_1^2\log(2q^*)/n\}^{1/2} + 144t^2B^2M_1^2/n\right],$$
$$\mathbb{E}\{\exp(12tJ_2)\} \le 2\exp\left[\{288t^2B^2M_2^2\log(2p)/n\}^{1/2} + 144t^2B^2M_2^2/n\right],$$
$$\mathbb{E}\{\exp(12tJ_3)\} \le 2\exp\left[\{288t^2B^2M_0^2\log(6)/n\}^{1/2} + 144t^2B^2M_0^2/n\right].$$

Denote the three terms on the right hand side of each inequality as $2\exp(L_k)$, $k = 1, 2, 3$. By the monotonicity of the exponential function, we have

$$\frac{1}{3}\sum_{k=1}^3 \mathbb{E}\{\exp(12tJ_k)\} \le \frac{1}{3}\sum_{k=1}^3 2\exp(L_k) \le \max_k 2\exp(L_k) \le 2\exp(\sum_k L_k).$$

Therefore, for any $t > 0$ and $g > 0$ we have

$$\Pr(\mathbb{G}_n > g) = \Pr\{\exp(t\mathbb{G}_n) > \exp(tg)\} \le 2\exp(-tg + \sum_{k=1}^3 L_k) = 2\exp(-gt + at^2 + bt),$$

where

$$a = 144B^2(M_1^2/n + M_2^2/n + M_0^2/n),$$
$$b = 12B\big[\{2M_1^2\log(2q^*)/n\}^{1/2} + \{2M_2^2\log(2p)/n\}^{1/2} + \{2M_0^2\log(6)/n\}^{1/2}.$$

Let $t = (g - b)/(2a)$. Then we have $\Pr(\mathbb{G}_n > g) \le 2\exp\{-(g-b)^2/(4a)\}$. Let $g = b + \{4a\log(1/\epsilon)\}^{1/2}$. Then we have $\Pr(\mathbb{G}_n > g) \le 2\epsilon$. Finally, we observe that

$$b + (4a\log(1/\epsilon))^{1/2} \le 36B\big\{M_1\Lambda(n, q^*, \epsilon) + M_2\Lambda(n, p, \epsilon) + M_0\Lambda(n, 3, \epsilon)\big\}.$$

□

Proof of Lemma S18. Using the union bound, for any $t > 0$,

$$\Pr(O > t) \le 2p \exp\{-t^2/(2B^2)\}.$$

Combined with the trivial bound $\Pr(O > t) \le 1$, we have

$$\begin{aligned} &\Pr(O > t) \le 1, \text{ if } t \le \{2B^2 \log(2p)\}^{1/2}, \\ &\Pr(O > t) \le 2p \exp\{-t^2/(2B^2)\}, \text{ if } t > \{2B^2 \log(2p)\}^{1/2}. \end{aligned}$$

Hence,

$$\begin{aligned} \mathbb{E}(e^O) &= \int_0^\infty \Pr(e^O > t)dt = \int_{-\infty}^\infty \Pr\{O > s\} e^s ds \\ &\le \int_{-\infty}^{\{2B^2 \log(2p)\}^{1/2}} e^s ds + \int_{\{2B^2 \log(2p)\}^{1/2}}^\infty 2p \exp\{-s^2/(2B^2) + s\} ds \\ &\le \exp\left[\{2B^2 \log(2p)\}^{1/2}\right] \cdot \left[1 + \int_0^\infty \exp\{-u^2/(2B^2) + u\} du\right] \\ &\le \exp\left[\{2B^2 \log(2p)\}^{1/2}\right] \cdot \left\{1 + (2\pi B^2)^{1/2} \exp(B^2/2)\right\}. \end{aligned}$$

Since $1 + (2\pi B^2)^{1/2} \exp(B^2/2) \le 2 \exp(B^2)$, we have

$$\mathbb{E}(e^O) \le 2 \exp\left[\{2B^2 \log(2p)\}^{1/2} + B^2\right].$$

□

S3 Additional simulation results

Table S1: Mean values of the evaluation metrics (AUC, ER, MSE-P) under Scenarios I–VI in Section 4.1 with the corresponding standard deviations presented in the parentheses.

Scenario	Method	AUC	ER	MSE-P
I	PASS_{100}	0.922 (0.009)	0.040 (0.048)	0.010 (0.009)
I	SS^{prior}	0.927 (0.004)	0.021 (0.024)	0.006 (0.004)
I	pLASSO^{1}_{100}	0.843 (0.039)	0.179 (0.057)	0.063 (0.020)
I	pLASSO^{2}_{100}	0.914 (0.016)	0.044 (0.039)	0.015 (0.012)
I	ALASSO_{100}	0.826 (0.034)	0.241 (0.102)	0.075 (0.024)
I	LASSO_{100}	0.840 (0.027)	0.184 (0.041)	0.066 (0.014)
I	LASSO_{150}	0.858 (0.019)	0.150 (0.032)	0.053 (0.010)
I	LASSO_{200}	0.869 (0.015)	0.129 (0.023)	0.045 (0.008)
I	LASSO_{250}	0.877 (0.013)	0.113 (0.019)	0.040 (0.007)
I	LASSO_{300}	0.884 (0.011)	0.100 (0.018)	0.035 (0.006)
I	LASSO_{350}	0.889 (0.010)	0.090 (0.016)	0.031 (0.005)
I	LASSO_{400}	0.894 (0.009)	0.080 (0.015)	0.028 (0.005)
II	PASS_{100}	0.899 (0.017)	0.092 (0.053)	0.029 (0.013)
II	SS^{prior}	0.832 (0.008)	0.179 (0.017)	0.065 (0.005)
II	pLASSO^{1}_{100}	0.845 (0.046)	0.176 (0.062)	0.061 (0.021)
II	pLASSO^{2}_{100}	0.855 (0.024)	0.154 (0.040)	0.054 (0.012)
II	ALASSO_{100}	0.834 (0.038)	0.261 (0.116)	0.074 (0.026)
II	LASSO_{100}	0.843 (0.035)	0.180 (0.047)	0.063 (0.016)
II	LASSO_{150}	0.873 (0.023)	0.134 (0.033)	0.046 (0.011)
II	LASSO_{200}	0.887 (0.017)	0.110 (0.024)	0.038 (0.009)
II	LASSO_{250}	0.895 (0.014)	0.094 (0.020)	0.032 (0.007)
II	LASSO_{300}	0.901 (0.012)	0.083 (0.016)	0.028 (0.006)
II	LASSO_{350}	0.905 (0.010)	0.074 (0.014)	0.025 (0.005)
II	LASSO_{400}	0.908 (0.009)	0.067 (0.012)	0.022 (0.004)
III	PASS_{100}	0.874 (0.022)	0.130 (0.058)	0.041 (0.013)

Scenario	Method	AUC	ER	MSE-P
III	SS^{prior}	0.811 (0.010)	0.192 (0.017)	0.068 (0.005)
III	$pLASSO^{1}_{100}$	0.829 (0.052)	0.188 (0.055)	0.066 (0.019)
III	$pLASSO^{2}_{100}$	0.835 (0.027)	0.171 (0.037)	0.060 (0.012)
III	$ALASSO_{100}$	0.828 (0.045)	0.265 (0.125)	0.073 (0.026)
III	$LASSO_{100}$	0.836 (0.040)	0.181 (0.047)	0.063 (0.016)
III	$LASSO_{150}$	0.869 (0.024)	0.136 (0.033)	0.047 (0.011)
III	$LASSO_{200}$	0.884 (0.018)	0.110 (0.024)	0.038 (0.008)
III	$LASSO_{250}$	0.894 (0.014)	0.094 (0.019)	0.032 (0.007)
III	$LASSO_{300}$	0.900 (0.012)	0.084 (0.017)	0.028 (0.006)
III	$LASSO_{350}$	0.904 (0.010)	0.075 (0.014)	0.025 (0.005)
III	$LASSO_{400}$	0.908 (0.009)	0.068 (0.012)	0.023 (0.004)
IV	$PASS_{100}$	0.873 (0.020)	0.120 (0.051)	0.041 (0.013)
IV	SS^{prior}	0.774 (0.010)	0.229 (0.017)	0.086 (0.005)
IV	$pLASSO^{1}_{100}$	0.868 (0.031)	0.134 (0.056)	0.045 (0.018)
IV	$pLASSO^{2}_{100}$	0.829 (0.028)	0.180 (0.039)	0.065 (0.013)
IV	$ALASSO_{100}$	0.824 (0.038)	0.271 (0.113)	0.077 (0.025)
IV	$LASSO_{100}$	0.834 (0.032)	0.183 (0.047)	0.065 (0.015)
IV	$LASSO_{150}$	0.864 (0.023)	0.137 (0.032)	0.048 (0.011)
IV	$LASSO_{200}$	0.880 (0.017)	0.111 (0.025)	0.039 (0.009)
IV	$LASSO_{250}$	0.889 (0.015)	0.096 (0.021)	0.033 (0.007)
IV	$LASSO_{300}$	0.895 (0.012)	0.084 (0.016)	0.029 (0.006)
IV	$LASSO_{350}$	0.899 (0.010)	0.075 (0.014)	0.026 (0.005)
IV	$LASSO_{400}$	0.902 (0.009)	0.068 (0.012)	0.023 (0.004)
V	$PASS_{100}$	0.871 (0.025)	0.116 (0.062)	0.039 (0.016)

Scenario	Method	AUC	ER	MSE-P
V	SS^{prior}	0.719 (0.012)	0.274 (0.016)	0.105 (0.005)
V	$pLASSO^1_{100}$	0.816 (0.050)	0.194 (0.064)	0.070 (0.024)
V	$pLASSO^2_{100}$	0.792 (0.040)	0.217 (0.049)	0.080 (0.017)
V	$ALASSO_{100}$	0.793 (0.042)	0.335 (0.139)	0.094 (0.029)
V	$LASSO_{100}$	0.809 (0.036)	0.205 (0.049)	0.075 (0.016)
V	$LASSO_{150}$	0.840 (0.024)	0.162 (0.034)	0.058 (0.012)
V	$LASSO_{200}$	0.857 (0.017)	0.135 (0.024)	0.048 (0.009)
V	$LASSO_{250}$	0.868 (0.015)	0.115 (0.022)	0.041 (0.008)
V	$LASSO_{300}$	0.877 (0.013)	0.099 (0.019)	0.035 (0.007)
V	$LASSO_{350}$	0.883 (0.011)	0.087 (0.017)	0.030 (0.006)
V	$LASSO_{400}$	0.889 (0.010)	0.078 (0.014)	0.027 (0.005)
VI	$PASS_{100}$	0.780 (0.034)	0.211 (0.055)	0.077 (0.016)
VI	SS^{prior}	0.712 (0.012)	0.258 (0.016)	0.100 (0.006)
VI	$pLASSO^1_{100}$	0.762 (0.054)	0.232 (0.054)	0.086 (0.019)
VI	$pLASSO^2_{100}$	0.761 (0.038)	0.226 (0.042)	0.085 (0.015)
VI	$ALASSO_{100}$	0.755 (0.054)	0.385 (0.165)	0.107 (0.032)
VI	$LASSO_{100}$	0.770 (0.051)	0.221 (0.052)	0.083 (0.018)
VI	$LASSO_{150}$	0.814 (0.029)	0.173 (0.034)	0.064 (0.013)
VI	$LASSO_{200}$	0.835 (0.021)	0.144 (0.029)	0.052 (0.010)
VI	$LASSO_{250}$	0.850 (0.017)	0.121 (0.023)	0.044 (0.009)
VI	$LASSO_{300}$	0.860 (0.015)	0.104 (0.020)	0.037 (0.007)
VI	$LASSO_{350}$	0.868 (0.013)	0.092 (0.017)	0.032 (0.006)
VI	$LASSO_{400}$	0.874 (0.012)	0.082 (0.016)	0.029 (0.006)

Table S2: Mean values of the evaluation metrics (AUC, ER, MSE-P) under Scenarios i–iii introduced in Section 4.2 with the corresponding standard deviations presented in the parentheses.

Scenario	Method	AUC	ER	MSE-P
i	$PASS_{100}$	0.875 (0.012)	0.045 (0.049)	0.013 (0.010)
i	SS^{prior}	0.881 (0.006)	0.024 (0.023)	0.007 (0.005)
i	$pLASSO^{1}_{100}$	0.862 (0.017)	0.068 (0.046)	0.021 (0.012)
i	$pLASSO^{2}_{100}$	0.877 (0.018)	0.030 (0.035)	0.010 (0.011)
i	$ALASSO_{100}$	0.774 (0.046)	0.269 (0.142)	0.078 (0.030)
i	$LASSO_{100}$	0.794 (0.041)	0.154 (0.045)	0.059 (0.016)
i	$LASSO_{200}$	0.834 (0.018)	0.099 (0.022)	0.037 (0.008)
i	$LASSO_{300}$	0.848 (0.012)	0.075 (0.017)	0.027 (0.006)
i	$LASSO_{400}$	0.858 (0.010)	0.058 (0.013)	0.021 (0.005)
i	$LASSO_{500}$	0.865 (0.008)	0.048 (0.011)	0.017 (0.004)
i	$LASSO_{600}$	0.869 (0.007)	0.040 (0.009)	0.014 (0.003)
i	$LASSO_{700}$	0.872 (0.006)	0.034 (0.008)	0.012 (0.003)
ii	$PASS_{100}$	0.898 (0.017)	0.065 (0.044)	0.020 (0.013)
ii	SS^{prior}	0.827 (0.008)	0.165 (0.016)	0.061 (0.005)
ii	$pLASSO^{1}_{100}$	0.901 (0.014)	0.061 (0.039)	0.018 (0.011)
ii	$pLASSO^{2}_{100}$	0.837 (0.017)	0.154 (0.032)	0.056 (0.010)
ii	$ALASSO_{100}$	0.815 (0.038)	0.237 (0.108)	0.075 (0.026)
ii	$LASSO_{100}$	0.828 (0.028)	0.180 (0.042)	0.066 (0.014)
ii	$LASSO_{200}$	0.867 (0.017)	0.112 (0.026)	0.040 (0.010)
ii	$LASSO_{300}$	0.887 (0.012)	0.078 (0.018)	0.027 (0.007)
ii	$LASSO_{400}$	0.897 (0.009)	0.059 (0.014)	0.020 (0.005)
ii	$LASSO_{500}$	0.903 (0.007)	0.049 (0.011)	0.016 (0.004)
ii	$LASSO_{600}$	0.906 (0.006)	0.041 (0.009)	0.013 (0.003)
ii	$LASSO_{700}$	0.908 (0.005)	0.036 (0.007)	0.012 (0.003)
iii	$PASS_{100}$	0.902 (0.022)	0.120 (0.055)	0.038 (0.016)
iii	SS^{prior}	0.852 (0.007)	0.192 (0.019)	0.068 (0.005)
iii	$pLASSO^{1}_{100}$	0.838 (0.035)	0.226 (0.054)	0.080 (0.018)
iii	$pLASSO^{2}_{100}$	0.849 (0.015)	0.198 (0.037)	0.070 (0.010)
iii	$ALASSO_{100}$	0.830 (0.032)	0.262 (0.089)	0.086 (0.023)
iii	$LASSO_{100}$	0.844 (0.025)	0.220 (0.039)	0.078 (0.013)
iii	$LASSO_{200}$	0.872 (0.014)	0.162 (0.023)	0.057 (0.008)
iii	$LASSO_{300}$	0.892 (0.011)	0.125 (0.019)	0.043 (0.007)
iii	$LASSO_{400}$	0.903 (0.009)	0.103 (0.016)	0.035 (0.006)
iii	$LASSO_{500}$	0.911 (0.008)	0.088 (0.013)	0.030 (0.005)
iii	$LASSO_{600}$	0.916 (0.006)	0.076 (0.011)	0.026 (0.004)
iii	$LASSO_{700}$	0.921 (0.005)	0.067 (0.009)	0.022 (0.003)

S4 Result Table for Real Data Analysis

Table S3: Median of the metrics AUC and standardized Brier Skill Score (BSS) on the three real EHR datasets, with their average standard error indicated in the subscripts.

(a) CAD

		PASS	SS^{prior}	SS^{ULASSO}	$pLASSO^2$	ULASSO	LASSO	ALASSO
AUC	$n = 50$	$0.891_{0.021}$	$0.886_{0.013}$	$0.881_{0.013}$	$0.877_{0.025}$	0.769_{0}	$0.853_{0.05}$	$0.855_{0.052}$
	$n = 70$	$0.894_{0.018}$	$0.889_{0.009}$	$0.884_{0.008}$	$0.882_{0.02}$	0.769_{0}	$0.871_{0.034}$	$0.871_{0.035}$
	$n = 90$	$0.897_{0.015}$	$0.891_{0.007}$	$0.886_{0.006}$	$0.886_{0.015}$	0.769_{0}	$0.882_{0.026}$	$0.88_{0.03}$

		PASS	SS^{prior}	SS^{ULASSO}	$pLASSO^2$	ULASSO	LASSO	ALASSO
BSS	$n = 50$	$0.5_{0.057}$	$0.489_{0.04}$	$0.475_{0.042}$	$0.453_{0.065}$	0_{0}	$0.368_{0.094}$	$0.408_{0.122}$
	$n = 70$	$0.51_{0.046}$	$0.502_{0.025}$	$0.49_{0.025}$	$0.473_{0.05}$	0_{0}	$0.417_{0.07}$	$0.444_{0.084}$
	$n = 90$	$0.516_{0.035}$	$0.508_{0.019}$	$0.497_{0.019}$	$0.489_{0.036}$	0_{0}	$0.447_{0.053}$	$0.47_{0.065}$

(b) RA

		PASS	SS^{prior}	SS^{ULASSO}	$pLASSO^2$	ULASSO	LASSO	ALASSO
AUC	$n = 50$	$0.935_{0.009}$	$0.934_{0.009}$	$0.932_{0.011}$	$0.93_{0.015}$	0.891_{0}	$0.898_{0.05}$	$0.897_{0.053}$
	$n = 125$	$0.939_{0.006}$	$0.938_{0.003}$	$0.934_{0.005}$	$0.937_{0.008}$	0.891_{0}	$0.933_{0.012}$	$0.931_{0.014}$
	$n = 200$	$0.941_{0.005}$	$0.938_{0.002}$	$0.932_{0.003}$	$0.94_{0.007}$	0.891_{0}	$0.94_{0.01}$	$0.936_{0.011}$

		PASS	SS^{prior}	SS^{ULASSO}	$pLASSO^2$	ULASSO	LASSO	ALASSO
BSS	$n = 50$	$0.595_{0.049}$	$0.598_{0.047}$	$0.591_{0.046}$	$0.585_{0.05}$	0.345_{0}	$0.494_{0.088}$	$0.508_{0.109}$
	$n = 125$	$0.631_{0.024}$	$0.629_{0.017}$	$0.615_{0.019}$	$0.625_{0.03}$	0.345_{0}	$0.604_{0.044}$	$0.599_{0.05}$
	$n = 200$	$0.637_{0.019}$	$0.635_{0.01}$	$0.616_{0.013}$	$0.637_{0.027}$	0.345_{0}	$0.633_{0.034}$	$0.622_{0.04}$

(c) Depression

		PASS	SS^{prior}	SS^{ULASSO}	$pLASSO^2$	ULASSO	LASSO	ALASSO
AUC	$n = 50$	$0.844_{0.034}$	$0.747_{0.02}$	$0.743_{0.024}$	$0.797_{0.043}$	0.496_{0}	$0.796_{0.062}$	$0.8_{0.066}$
	$n = 85$	$0.866_{0.021}$	$0.751_{0.013}$	$0.751_{0.013}$	$0.834_{0.035}$	0.496_{0}	$0.846_{0.033}$	$0.847_{0.034}$
	$n = 120$	$0.872_{0.016}$	$0.753_{0.009}$	$0.754_{0.009}$	$0.857_{0.028}$	0.496_{0}	$0.864_{0.023}$	$0.865_{0.023}$

		PASS	SS^{prior}	SS^{ULASSO}	$pLASSO^2$	ULASSO	LASSO	ALASSO
BSS	$n = 50$	$0.311_{0.084}$	$0.144_{0.05}$	$0.138_{0.049}$	$0.219_{0.082}$	0_{0}	$0.217_{0.102}$	$0.212_{0.13}$
	$n = 85$	$0.371_{0.056}$	$0.165_{0.032}$	$0.161_{0.028}$	$0.296_{0.067}$	0_{0}	$0.323_{0.065}$	$0.326_{0.085}$
	$n = 120$	$0.392_{0.039}$	$0.173_{0.021}$	$0.168_{0.018}$	$0.343_{0.056}$	0_{0}	$0.364_{0.051}$	$0.372_{0.063}$

References

Belloni, A. and Chernozhukov, V. (2011). ℓ_1-penalized quantile regression in high-dimensional sparse models. *The Annals of Statistics*, 39(1):82–130.

Bickel, P. J., Ritov, Y., and Tsybakov, A. B. (2009). Simultaneous analysis of lasso and dantzig selector. *The Annals of Statistics*, 37(4):1705–1732.

Koltchinskii, V. (2011). *Oracle Inequalities in Empirical Risk Minimization and Sparse Recovery Problems*. Springer.

Li, K.-C. and Duan, N. (1989). Regression analysis under link violation. *The Annals of Statistics*, 17(3):1009–1052.

Tibshirani, R. J. et al. (2013). The lasso problem and uniqueness. *Electronic Journal of statistics*, 7:1456–1490.

van de Geer, S. A. (2016). *Estimation and testing under sparsity*. Springer.

van de Geer, S. A. and Bühlmann, P. (2009). On the conditions used to prove oracle results for the lasso. *Electronic Journal of Statistics*, 3:1360–1392.

Zhao, P. and Yu, B. (2006). On model selection consistency of lasso. *Journal of Machine learning research*, 7(Nov):2541–2563.

Zou, H. and Zhang, H. H. (2009). On the adaptive elastic-net with a diverging number of parameters. *The Annals of Statistics*, 37(4):1733–1751.
